# Computational study on the binding of Mango-II RNA aptamer and fluorogen using the polarizable force field AMOEBA

**DOI:** 10.3389/fmolb.2022.946708

**Published:** 2022-09-02

**Authors:** Xudong Yang, Chengwen Liu, Yu-An Kuo, Hsin-Chih Yeh, Pengyu Ren

**Affiliations:** ^1^ Department of Biomedical Engineering, The University of Texas at Austin, Austin, TX, United States; ^2^ Texas Materials Institute, University of Texas at Austin, Austin, TX, United States; ^3^ Oden Institute for Computational Engineering and Science, Austin, TX, United States; ^4^ Interdisciplinary Life Science Graduate Programs, Austin, TX, United States

**Keywords:** fluorescent light-up aptamers (FLAPs), aptamer-fluorogen binding, AMOEBA potential, force field (FF), free energy calculation

## Abstract

Fluorescent light-up aptamers (FLAPs) are well-performed biosensors for cellular imaging and the detection of different targets of interest, including RNA, non-nucleic acid molecules, metal ions, and so on. They could be easily designed and emit a strong fluorescence signal once bound to specified fluorogens. Recently, one unique aptamer called Mango-II has been discovered to possess a strong affinity and excellent fluorescent properties with fluorogens TO1-Biotin and TO3-Biotin. To explore the binding mechanisms, computational simulations have been performed to obtain structural and thermodynamic information about FLAPs at atomic resolution. AMOEBA polarizable force field, with the capability of handling the highly charged and flexible RNA system, was utilized for the simulation of Mango-II with TO1-Biotin and TO3-Biotin in this work. The calculated binding free energy using published crystal structures is in excellent agreement with the experimental values. Given the challenges in modeling complex RNA dynamics, our work demonstrates that MD simulation with a polarizable force field is valuable for understanding aptamer-fluorogen binding and potentially designing new aptamers or fluorogens with better performance.

## Introduction

Ribonucleic acid (RNA) is a ubiquitous macromolecule playing an important role in all domains of life, possessing diverse functions in protein synthesis (Merrick and Pavitt, 2018) and regulation of many cellular pathways and gene expression (Derrien et al., 2012; Neueder et al., 2018). Therefore, imaging RNA of interest (ROI) has become a crucial strategy to understand many cellular pathways and physiological processes. Green fluorescent protein (GFP) can generate high background fluorescence when bound to ROI (Rackham and Brown, 2004; Daigle and Ellenberg, 2007; Ozawa et al., 2007), while it falls short due to the large size of the fused complex. The huge complex causes a long transfer time, interrupts the biological function of ROI, and limits the scale of application (Tyagi, 2009). All these drawbacks of GFP have led to the development of RNA labeling systems with similar photophysical properties. As a result, the RNA sequences called fluorescent light-up aptamers (FLAPs) (Zhou and Zhang, 2021) have been discovered and developed to study the RNA function both *in vitro* and *in vivo* (Huang et al., 2017; Okuda et al., 2017).

FLAPs can be genetically fused to ROI and bind their cognate fluorogens with high affinity. Such an interaction switches fluorogens to a highly fluorescent state, while the unbound fluorogens in solution manifest minimal signal (Zhou and Zhang, 2021). Meanwhile, the small size of FLAPs is beneficial for fast movement in the cell, easy programming, and minor influence on cell function. Recently, a specific fluorogen TO1-biotin (Trachman et al., 2018) has its fluorescence enhanced by more than 1,500 fold upon binding to Mango-II aptamer (Autour et al., 2018) with high affinity, which becomes a remarkable candidate for efficient ROI tracking. In addition to ROI, FLAPs are also capable of targeting many non-nucleic acid molecules including proteins (Song et al., 2013; Strack et al., 2014), metabolites (Paige Jeremy et al., 2012; Strack et al., 2014), and coenzyme factors (Zhang et al., 2019), which reflects the significance of FLAPs in studying the mechanisms of various biological processes.

Nevertheless, there are only limited approaches to discovering or improving FLAPs to date. The first fluorogenic RNA aptamer, Spinach, was selected through systematic evolution of ligands by exponential enrichment (SELEX) (Paige Jeremy et al., 2011). SELEX isolates high-affinity FLAPs from 10^13^ ∼ 10^15^ distinct sequences and is currently a popular method to discover new nucleic acid aptamers (Paige Jeremy et al., 2011; Dey et al., 2022). Alternatively, microfluidic-assisted *in vitro* compartmentalization (µIVC) approach can also be used to discover new aptamers. Taking advantage of digital droplet PCR, for instance, iSpinach and Mango II-IV aptamers have been identified by the Ryckelynck group and Unrau group (Autour et al., 2016; Autour et al., 2018). However, SELEX and µIVC processes usually take rounds of tedious and laborious selection and use a specific instrument. In contrast, without re-selection, the Jaffrey group demonstrated that both the RNA aptamer and its cognate fluorogen could be re-designed to create a new construct having red-shifted fluorescence emission using prior chemistry knowledge (Li et al., 2020).

**FIGURE 1:**
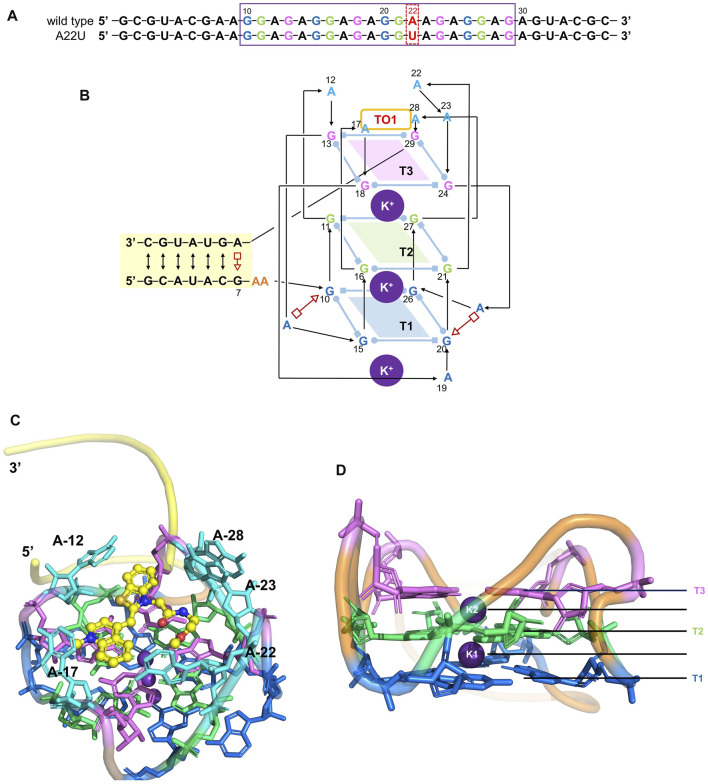
Structure of the Mango-II. **(A)**: nucleic acid sequence of both wild-type and A22U mutate of Mango-II, with the purple box highlighting the G-quadruplex component (site 10–29). **(B)**: Secondary structure of the wild-type complex. Base pairs are represented with Leontis−Westhof symbols, and the connectivity of the main chain is denoted by arrows and lines. Three G-quadruplex layers have been illustrated by blue (T1), green (T2), and violet (T3) planes. **(C)**: Cartoon representation of the crystal structure of Mango-II_TO1-Biotin complex (PDB id: 6C63) **(D)**. Side view of the G-quadruplex (2 K^+^ have been abbreviated as K1 and K2).

**FIGURE 2:**
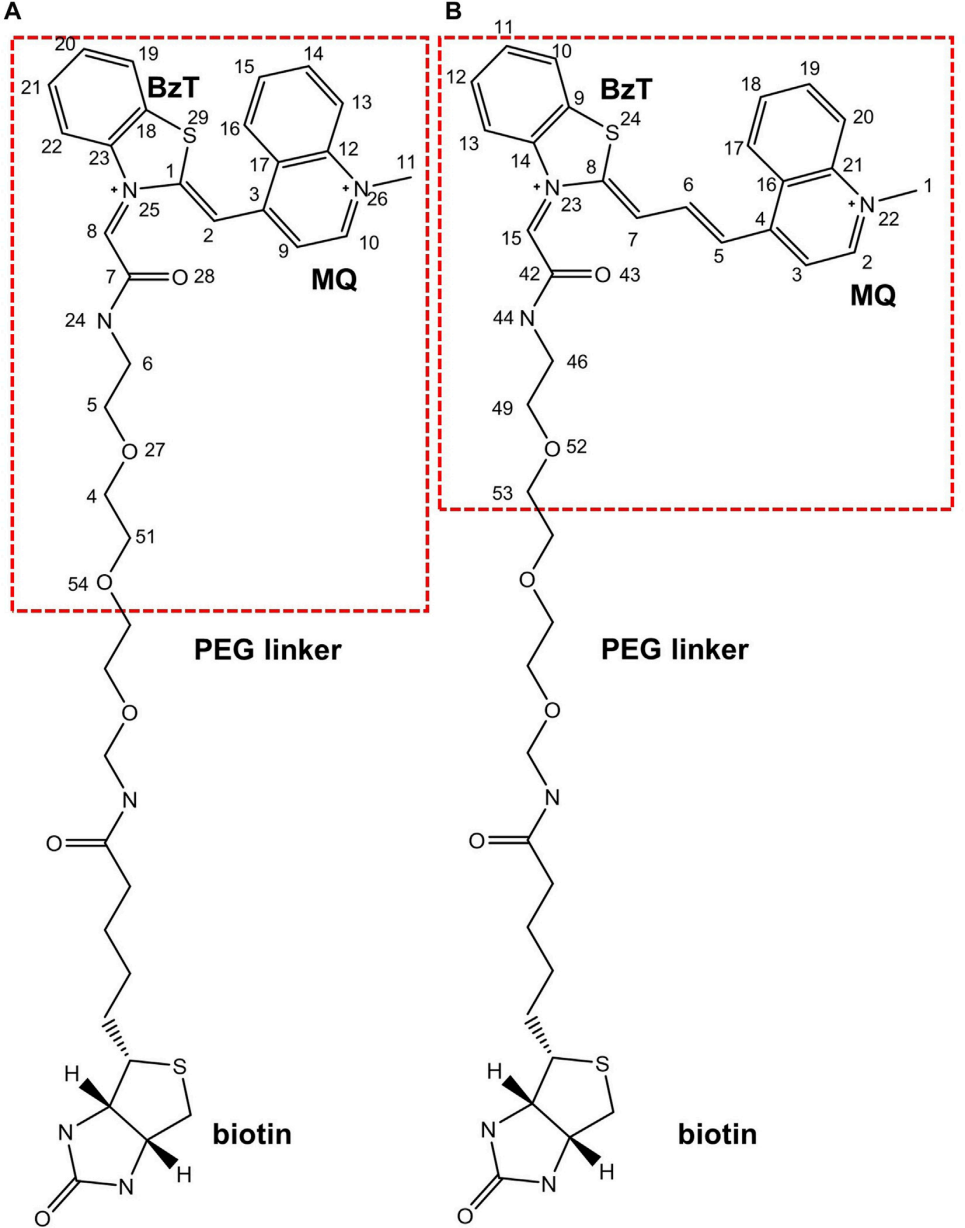
Chemical structures of **(A)**. TO1-Biotin and **(B)**. TO3-Biotin. BzT means benzothiazole and MQ mean methyl quinoline. The red dash box represents the part inside the binding pocket.

Since it is difficult to develop aptamers with high affinity, and even more challenging to simultaneously enhance the affinity and fluorescent properties of existing FLAP-fluorogen pairs in experiments, computational strategies can be utilized to reveal the specific mechanism underlying the binding and provide rational guidance in designing efficient FLAPs. AMOEBA (Ren and Ponder, 2003; Zhang et al., 2018) is a polarizable force field (FF) capable of accurately depicting the interactions between complex biosystems, especially highly charged macromolecules, and ligands. Because Mango-II possesses a strongly twisted double strand component and a three-layer G-quadruplex held together by potassium ions (see Figure 1), it is not suitable for classical force fields without polarization effects to model the structure and describe the interactions (Lemkul and MacKerell, 2017a; Lemkul and MacKerell, 2017b; Salsbury and Lemkul, 2019). The polarizable multipole-based AMOEBA model has been well demonstrated on DNA/RNA systems in previous works (Zhang et al., 2018; Jing et al., 2019). Recently, TO1-Biotin and TO3-Biotin ligands have been found out sharing an almost similar affinity in binding to Mango-II (Trachman et al., 2018) (Figure 2). The binding pocket of Mango-II is smaller than that of Mango-I (Autour et al., 2018). Only part of the ligands (BzT and MQ in Figure 2) has a direct interaction with the Mango-II, and the PEG linker is long enough to separate the whole ligands into two relatively isolated parts.

Here we applied AMOEBA (Ren and Ponder, 2003; Zhang et al., 2018) to examine the detailed interactions between Mango-II and specified fluorogens and evaluated their binding free energies from molecular dynamics simulations. Moreover, one mutant of Mango-II (A22U) has been investigated for the effect of mutation on binding. Our predicted binding free energy values are in excellent agreement with the prior reported experimental values (within 10% error), in both wildtype Mango-II and A22U mutant with known crystal structures. We believe this work represents the first effort to model RNA binding with fluorogen molecules and to compute rigorous binding thermodynamics of RNA-ligand from all-atom simulations using an advanced polarizable force field.

## Methods

The AMOEBA FF for nucleic acids (Zhang et al., 2018) has been utilized for wildtype Mango-II and its mutant. The parameters of fluorogens were derived from Polytype 2 program (Wu et al., 2012a). Here is a brief workflow in parametrization: First, the fluorogen structures were optimized at the MP2/6–31 g* level of theory. The optimized structures were used to derive the atomic multipoles. Next, atomic multipoles were initially derived from QM electron density calculated at the MP2/6-311G** level of theory via the distributed multipole analysis (DMA) (Stone, 2005) approach. Subsequently, the dipole and quadrupole moments were further optimized by targeting the electrostatic potential of the MP2/aug-cc-pvtz level of theory. The POTENTIAL program in Tinker (Rackers et al., 2018) was utilized in this process. Finally, the van der Waals, polarizability (Litman et al., 2022), and the torsion parameters were automatically assigned. For the torsion parameters that could not be found in the database, a fragment-based fitting scheme was performed and the fitted parameters were transferred to the ligands. The detailed process can be accessed on the publicly accessible Github site (Wu et al., 2009; Wu et al., 2012b). All the quantum mechanics (QM) calculations inside Poltype 2 were performed with Psi4 software (Smith et al., 2020).

The crystal structures of FLAP-fluorogen complexes were taken from the Protein Data Bank: Mango-II_TO1-Biotin from PDB ID 6C63, Mango-II_TO3-Biotin from 6C64, and Mango-II-A22U_TO1-Biotin from 6C65. However, since there is no Mango-II-A22U_TO3-Biotin crystal structure so far, a starting structure was generated by replacing TO1-Biotin in 6C65 with TO3-Biotin. All the systems were neutralized by K^+^ in a water box with a size of 60 Å × 70 Å × 65 Å such that the distance between the FLAP and its periodic images was at least 12 Å. Additional KCl was added to obtain 0.15 mol/L salt concentration to mimic the correct physiological condition. The systems contain ∼25,000 atoms and were subject to NPT simulations to relax to desired densities. Both the ligands (TO1-Biotin and TO3-Biotin) were trimmed at a certain point and remained the part (red box in Figure 2) with direct interaction with Mango-II for computational efficiency. From Ref (Trachman et al., 2018), Trachman et al. tested a set of truncated ligands with different numbers of atoms remaining at the linker (no Biotin on them). They found the free energy could converge to a value very close to that with a full PEG linker and Biotin when the remaining linker is long enough. The chosen remaining length of the linker in this work is much longer over the convergence point. Moreover, biotin might impact the binding, but the experimental values show the error of removing Biotin is minimal and acceptable. Therefore, it is reasonable to use truncated ligands in the computational study. In this work, we called those truncated ligands their initial names: TO1-Biotin and TO3-Biotin.

The systems were initially minimized in Tinker (Rackers et al., 2018), and then subject to a 2 ns gradual heating process to reach 298K under NPT ensemble at 1atm, with RNA-ligand atoms restrained. At 298K, restrained MD simulations on all atoms of the complex for 1 ns were performed followed by equilibration of the whole system without any restraint for 10 ns under NPT ensemble at 1 atm. All the complexes were observed to reach equilibrium at ∼4 ns from the RMSD of the heavy atoms from the G-quadruplex part (bases 10–29) (Supplementary Figure S1–3). The frame at 4ns was extracted for subsequent free energy simulations. For the conversion to configuration B of Mango-II-A22U_TO1-Biotin, it took a long time to equilibrate. The total 35 ns MD simulation was performed on this configuration, and it reached equilibrium at around 15 ns (Supplementary Figures S5,6). The snapshot at 15 ns was chosen for the subsequent free energy analysis. The MD simulations were propagated by using the RESPA integrator (Tuckerman et al., 1992) with a 2.0 fs time step. Temperature and pressure were controlled by the Bussi thermostat (Bussi et al., 2007) and Monte Carlo barostat (Frenkel et al., 1996) respectively. The van der Waals (vdW) iterations used a 12.0 Å cut-off, while the electrostatic interactions were treated by PME with a real-space cutoff of 7.0 Å. The induced dipoles were calculated through self-consistent iterations with a convergence tolerance of $1\times 10^{-4}$ Debye. All MD simulations were carried out using the Tinker nine program on graphical processing units (GPUs).

The absolute binding free energies were calculated by the alchemical double annihilation method. The formula to calculate the free energies ∆G can be expressed as Eq. 1:

$$\begin{array}{c} \Delta G=\Delta G_{FLAP\to complex}-\Delta G_{solv} \end{array}$$ (1)

$\Delta G_{FLAP\to complex}$ is the free energy change due to annihilating ligand in the RNA-ligand complex solution, and $\Delta G_{solv}$ refers to the free energy of annihilating fluorogen ligand in water. The annihilation end state of the two simulations is the same for the ligand, where the ligand atoms have no electrostatic or vdw interactions with other atoms in the system.

The “annihilation” is done by scaling electrostatic parameters of the FLAP and vdw interactions between the ligand and surrounding in several stages (states) by using $\lambda_{ele}$ and $\lambda_{vdw}$ parameters respectively. The coefficients $\lambda_{\text{vdw}}=0$ and $\lambda_{\text{ele}}=0$ denote the state in which the fluorogen is not interacting with the rest of the system (unbinding or desolvation). The stages of the annihilation process from unbound FLAP to the complex were described by $\lambda_{\text{vdw}}$ values as 0.0, 0.5, 0.55, 0.6, 0.62, 0.65, 0.7, 0.75, 0.8, 0.9, 1.0 (while $\lambda_{ele}=0$ ). and then $\lambda_{\text{ele}}$ increasing linearly from 0.0 to 1.0 with the interval 0.1 (i.e., 21 states) with $\lambda_{vdw}=1$ . To guarantee the ligand stayed in the binding pocket when λ=0 , a harmonic restraint between the fluorogen and RNA was used (details in Supplementary Table S4). In this work, the harmonic restraint is added between the center of mass (COM) of the ligand and K2 (K^+^ between T3 and T2 layers). A restraint correction was calculated to remove the effect of restraint and the specific equation for that can be accessed in Ref. (Boresch et al., 2003; Hamelberg and McCammon, 2004; Jiao et al., 2008). A total of 6 ns simulations were performed for each stage, with the last 5 ns trajectories for free energy calculations. Thus, a simulation of the total 126 ns was performed on each complex to obtain one binding free energy. The free energy changes between adjacent states were calculated by the Bennett acceptance ratio (BAR) method (Bennett, 1976; Shirts and Chodera, 2008; Bell et al., 2016) of Tinker implementation, and the energy contribution from the restraint has been corrected by using the FREEFIX program in Tinker. The calculation of solvation free energy of ligands in water boxes follows the same procedure without any extra restraint.

## Results and discussion

### Simulation of wild-type Mango-II without ligand

First, we checked the structural stability of Mango-II without any ligands. To better quantify the stacking structures, an important structural measure, the layer distance, has been defined as the distance between the mass center of each layer (or base ring). In the following analysis, the layer distance between the base parts of any two residues is directly represented by the pair of their name. For example, A23-A28 denotes the layer distance between the nucleobases of A23 and A28. Also, it is convenient to identify any atom of ligands with an index: for example 24N meaning the nitrogen with an index of 24 in the ligand. The indexing of all the ligands can be found in Figure 2.

The polarization effects are very important to stabilize the third-layer G-quadruplex and the positions of interior K^+^. It can be observed from RMSDs (heavy atoms of the core part) of wild-type Mango-II with and without polarization terms (Supplementary Figure S1, 2): RMSD with polarization reaches ∼2.5 Å while RMSD without polarization comes to ∼3 Å. Meanwhile, the positions of K^+^ in the G-quadruplex are much more obvious to reflect the stability of this structure. The K^+^ near the surface was moving out of the complex, which means that only two interior K^+^ (K1 and K2 in Figure 1D) between the three layers are playing a major role in the stabilization of this structure. The position of interior K^+^ can be represented by the distance to the COM of the heavy atoms on base cycles from all nucleotides of the specified layer, which provides four distances (T3-K2, T2-K2, T2-K1, T1-K1) in this system. In Supplementary Figure S9; Supplementary Table S2, these four distances are fluctuating around 1.8 Å when polarization is considered. And it indicates that the layer distances were kept in the range of 3.0–3.5 Å. However, when the polarization was removed, the whole structure collapsed. It can be easily found from Supplementary Figure S10 that all four distances leap to very high values and are far from a stable state. As a result, the significance of including polarization has been proven for such an RNA system.

The stability of the three-layer G-quadruplex for other complexes with ligands has been also tested in this work. First, the RMSD of all the other complexes can be found in Supplementary Figures S3, 5, and 7. In addition, Supplementary Figures S11–16 record the interior K^+^ positions from six complexes with ligands during the equilibration, and Supplementary Table S2 gives the specific statistical data. Because the alchemy process involves too many simulations, only the statistical data of interior K^+^ positions are given in Supplementary Table S3. In general, the K2 becomes much closer to T3 than the T2 layer when the ligand exists, indicating that the ionic ligand shows electric interaction with K2.

Although the function of interior K^+^ is significant for stabilization, the ligand does not have any obvious impact on the formation of the G-quadruplex since this structure was not altered when removing the ligand.

**FIGURE 3:**
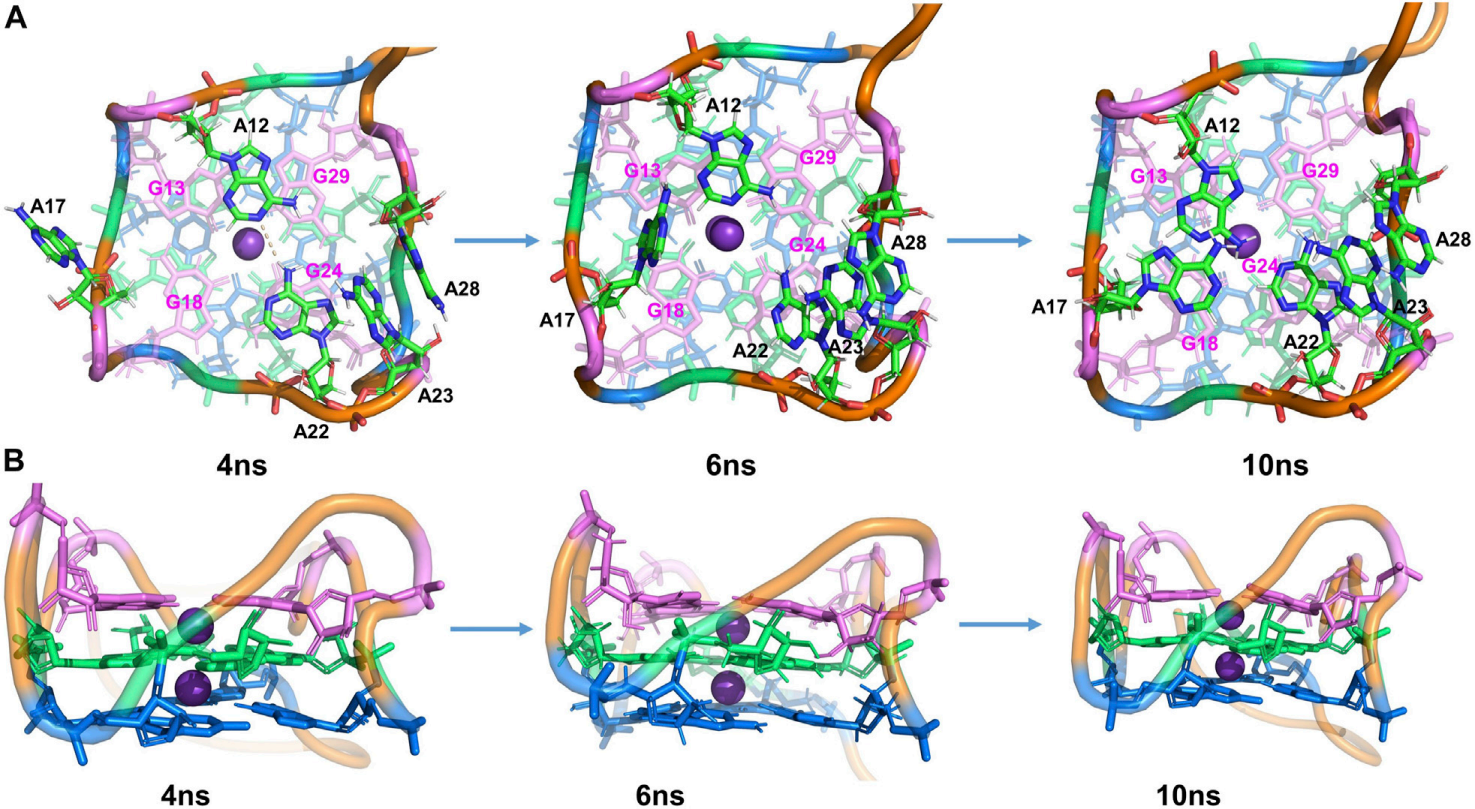
Structural analysis of MD simulation on wild-type Mango-II without ligand: the process of structural change during the MD (4 ns, 6 ns, 10 ns snapshots). **(A)**: top view. **(B)**: Side view.

Furthermore, additional interactions were found in the surrounding adenines (A12, A17, A22, A23, and A28), keeping the whole structure stable during the MD process. From Figure S4, the distance between A23-A28 is very stable during the MD, slightly fluctuating around 3.8 Å. At ∼1.5 ns, it is observed that the distance between A22-A23 and that of A22-G24 decreased significantly. By examining the simulated structure at 4 ns (Figure 3), it is clear that the π−π stackings between A22-A23 and A22-G24 are taking over during this period, suggesting that the RNA structure in the solution, especially the non-core (G-quad) region, is dynamic and can form stacking interactions not observed in the X-ray structures of RNA-ligand complexes. Also, one hydrogen bond (HB) was observed to form between A12 and A22 at ∼2.5 ns (Figure 3), which is diagonally crossing the pocket above the T3 layer. In addition, the distance of N-H falls to 2 Å at ∼2.5 ns and is kept around 3 Å for about 2 ns, which shows that this HB lasts from 2.5 to 4.5 ns.

After 4.5 ns, owing to the stacking effects of A12-G13 becoming dominant, the HB of A12-A22 is broken. It can be proved by seeing that the distance of N-H is getting larger while the layer distance of A12-G13 is steadily decreasing to ∼3.8 Å in the period of 4.5–7 ns (Supplementary Figure S4). The A17 is also attracted to the binding pocket because of the stacking with G18 (Figure 3). As a result, the initial G-quadruplex is augmented into an even larger multi-layered structure with additional adenine stacking, by which the binding pocket is completely closed and occupied (see the snapshot at 10 ns in Figure 3). Nonetheless, the initial hexad of T1 with A14 and A25 no longer existed in an equilibrium structure. Both A14 and A25 stretched to the waterside and had no interaction with the T1 layer, indicating that it has minimal effects on the stability.

The simulations showed that the wild-type structure without any fluorogen is quite stable and the core (G-quadruple) remains unchanged from that of the complex, although our MD simulations sampled alternative interactions and structures among the peripheral nucleotides.

### The complexes of Mango-II with TO1-Biotin and TO3-Biotin

The complexes of Mango-II bounded with the TO1-Biotin and TO3-Biotin were subject to 10 ns NPT simulation to analyze the complex structures.

**FIGURE 4:**
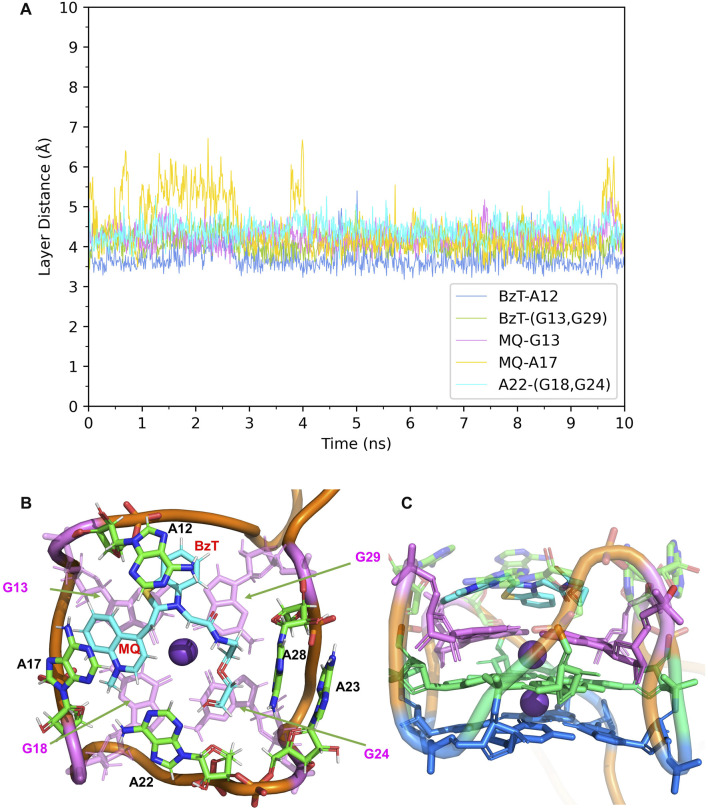
Structural analysis of MD simulation on wild-type Mango-II with TO1-Biotin. **(A)**: the trajectories of important structural parameters in the MD process. **(B)**: the top view of equilibrium structure from the MD process (5 ns snapshot), with the ligand shown in cyan and T3 guanines shown in pink. **(C)**: the side view.

In Mango-II_TO1-Biotin, the main interactions of the binding mode are the A12-BzT-T3 layer (G13 or G29), A17-MQ-T3 layer (G13 or G29), A22-T3 layer (G18 or G24), and A23-A28, which are illustrated in Figure 4B. From Table 1; Figure 4B, it takes about 4 ns for this complex to reach equilibrium in our simulations: the layer distance BzT-A12 is stable at around 3.66 Å, and the distances MQ-G13 and MQ-A17 are fluctuating around 4.24 Å A22 is always stacking with the T3 layer (G18 or G24), occupying a large part of the binding pocket to restrain TO1-Biotin near A12 and G13. The PEG linker would go freely through the wide space between A21 and A22 to connect the Biotin outside the binding pocket, so the steric hindrance is avoided.

**TABLE 1:** Binding modes and structural data of aptamer-fluorogen complexes from MD simulations.

Aptamer	Fluorogen	Average layer distance (Å) ^(^ ^a^ ^)^	
Mango-II(WT)	TO1-Biotin	BzT - A12	3.66 ± 0.27
		BzT - (G13,G29) (^b)^	4.07 ± 0.23
		MQ - G13	4.25 ± 0.25
		MQ - A17	4.23 ± 0.41
		A22 - (G18,G24) (^c)^	4.41 ± 0.26
Mango-II(WT)	TO3-Biotin	BzT - A12	4.61 ± 0.57
		BzT - (G13,G29) (^b)^	4.48 ± 0.25
		MQ - G13	4.43 ± 0.50
		MQ - A17	3.74 ± 0.21
Mango-II-A22U	TO1-Biotin	Configuration A	
		BzT - A12	3.95 ± 0.47
		BzT - (G13,G29) (^b)^	4.37 ± 0.40
		MQ - G13	4.21 ± 0.31
		MQ - A17	4.07 ± 0.45
		U22 - G24	4.94 ± 0.42
		Configuration B	
		BzT - A12	5.02 ± 0.49
		BzT - (G13,G29) (^b)^	4.08 ± 0.26
		MQ - U22	3.56 ± 0.16
Mango-II-A22U	TO3-Biotin	Configuration A	
		BzT - A12	4.19 ± 0.47
		MQ - G13	3.83 ± 0.20
		MQ - A17	4.01 ± 0.35
		U22 - G24	3.84 ± 0.52
		Configuration B	
		BzT - A12	5.82 ± 1.02
		MQ - A12	6.93 ± 1.76
		MQ - G13	4.00 ± 0.27
		MQ - A17	4.27 ± 0.56
		BzT - G29	4.70 ± 0.37

a Average Layer Distance: the average of the layer distance of the specified stacking pairs from 4 to 10 ns MD, simulation. For configuration B, the average is from 15 to 30 ns. (b). (G13, G29) represents the center of mass of bases from both G13 and G29. (c). (G18, G24) represents the center of mass of bases from both G18 and G24.

**FIGURE 5:**
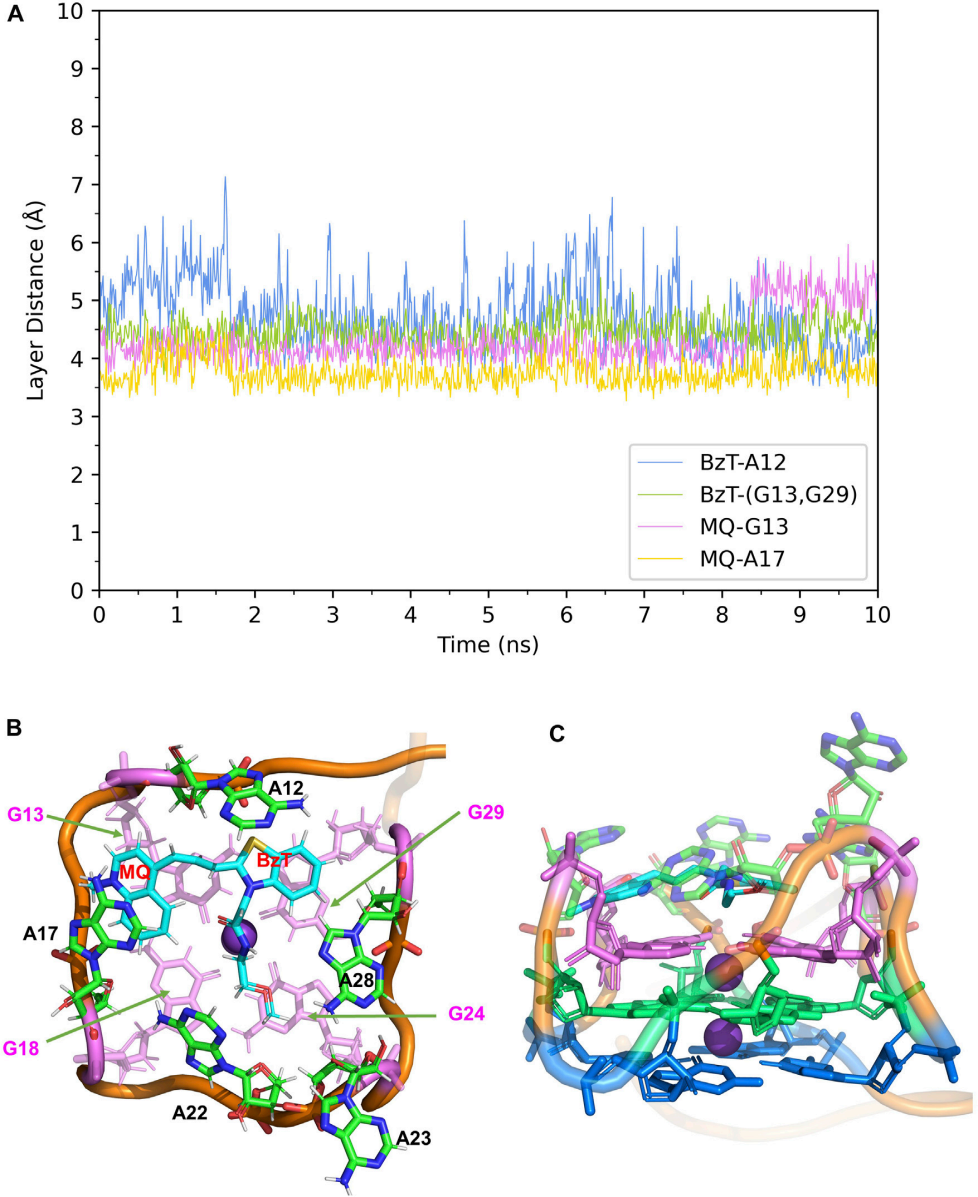
Structural analysis of MD simulation on wild-type Mango-II with TO3-Biotin. **(A)**: the trajectories of important structural parameters in the MD process. **(B)**: the top view of equilibrium structure from the MD process (4 ns snapshot), with the ligand shown in cyan and T3 guanines shown in pink. **(C)**: the side view.

In Mango-II_TO3-Biotin, the binding mode is similar to that in Mango-II_TO1-Biotin: A12-BzT-T3 layer (G13 or G29), A17-MQ-G13 and A22-T3 layer (G18) stacking are important to hold the ligand tightly in the binding pocket (Figure 5B). As shown in Figure 5A, the layer distances of MQ-G13 and MQ-A17 are stabilized at around 4 Å during the whole 10 ns MD simulation, and the average after equilibration is 4.43 Å and 3.74 Å, respectively (Table 1). Due to the extended linker between BzT and MQ (Figure 2), BzT moves almost beyond the binding region of A12 when MQ interacts with A17 and G13. Hence the interaction between BzT and A12 is not so stable, and the layer distance is oscillating in the range of 4–6 Å. In addition that, both BzT and the PEG linker are getting closer to A23, which disrupts the A23-A28 interaction.

The free energy calculations were performed on both complexes and the results are shown in Table 2, and the detailed free energy change between any neighbor states in the alchemical process has been recorded in Supplementary Tables S5–S12. The calculated free energy of TO1-Biotin binding to Mango-II is -11.91 kcal/mol while the free energy of TO3-Biotin binding to Mango-II is -11.07 kcal/mol. These calculated values are in good agreement with those from experimental measurements within ∼1 kcal/mol. Also, Mango-II shows a slight preference for TO1-Biotin according to experiments, which is also reflected in our results. These results fully verify the reliability of AMOEBA in the study of ligand binding in complicated RNA complexes. Moreover, the analysis of structures of both Mango-II_TO1-Biotin and Mango-II_TO3-Biotin explains the difference in free energies. The high commonality in the binding mode of both Mango-II_TO1-Biotin and Mango-II_TO3-Biotin accounts for the similar binding free energy. However, the loose stacking of BzT with A12 and the interrupted A23-A28 interaction causes relatively less stability of Mango-II_TO3-Biotin. Therefore, it is reasonable to see that the free energy of Mango-II_TO1-Biotin is slightly greater (more negative) than that of Mango-II_TO3-Biotin.

**TABLE 2:** Calculated and experimental binding free energies between aptamers and fluorogens (in kcal/mol).

Aptamer	Fluorogen	Calc.^(a)^	Exp.^(b)^
Mango-II(WT)	TO1-Biotin	-11.91 ± 0.45	-12.48 ∼ -12.15^(c)^
			-12.90 ∼ -12.35^(d)^
Mango-II(WT)	TO3-Biotin	-11.09 ± 0.44	-12.30 ∼ -12.04^(c)^
			-12.04 ∼ -11.97^(d)^
Mango-II-A22U	TO1-Biotin	-11.07 ± 0.84 (Configuration A)	-12.77 ∼ -12.20^(c)^
		-9.91 ± 0.43 (Configuration B)	
Mango-II-A22U	TO3-Biotin	-17.22 ± 0.38 (Configuration A)	-11.88 ∼ -11.12^(c)^
		-9.82 ± 0.39 (Configuration B)	

a Calc: calculated free energies by AMOEBA

b Exp: Experimental free energies.

c Value from **Ref** (Trachman et al., 2018)**.** (Free energies were converted from dissociation constant K_d_).

d Value from **Ref** (Autour et al., 2018)**.** (Free energies were converted from dissociation constant K_d_).

### The complexes of A22U mutation with TO1-Biotin and TO3-Biotin

The binding mode between Mango-II and two ligands has been illustrated from MD simulations as described above. Next, a mutated Mango-II structure (A22U) (Trachman et al., 2018), which potentially has better stereoselectivity and specificity, was investigated.

**FIGURE 6:**
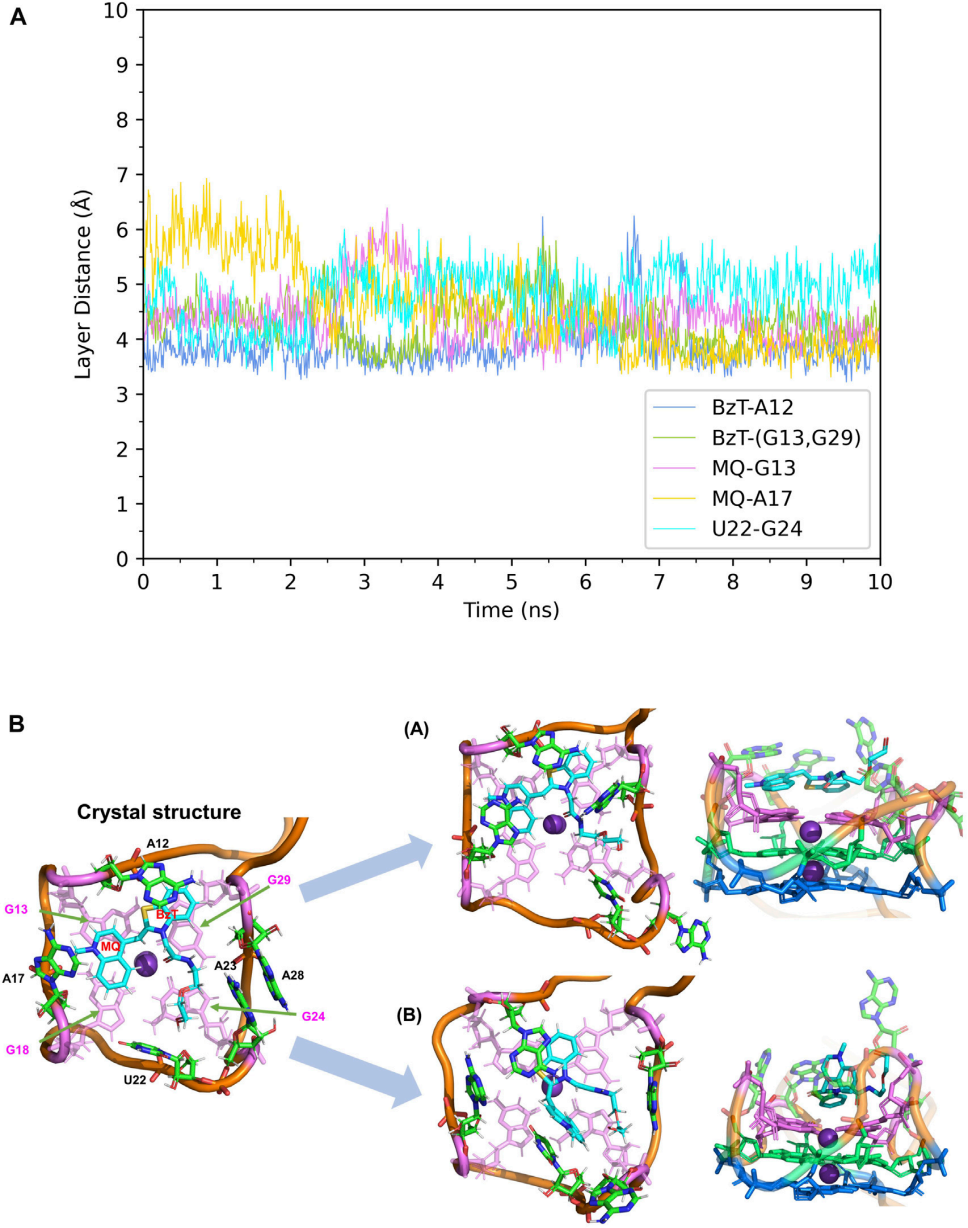
Structural analysis of MD simulation on Mango-II mutant (A22U) with TO1-Biotin. **(A)**: the trajectories of important structural parameters in the MD process to obtain configuration A. **(B)**: the two configurations of the equilibrium structure from the MD process (4 ns snapshot for configuration A, 15 ns snapshot for configuration B, with the ligand shown in cyan and T3 guanines shown in pink. (Side views have been placed on the right).


First, the crystal structure of Mango-II-A22U is given by PDB 6C65 and was used for simulation. From our simulations, two possible configurations emerged in the MD process, one of which matched crystal structure while another one show a 90° rotation of TO1-Biotin (Figure 6B). The binding free energy computed from the first configuration (A) is -11.07 ± 0.84 kcal/mol (Table 2), which is in agreement with the experimental value, and its binding mode resembles not only the crystal structure of the mutant but also the previous wild type. From Figure 6A, it is clear that all the important stacking pairs in configuration A were stable during the MD simulations. The layer distances of BzT-A12, MQ-G13, and MQ-A17 were kept around 4 Å, while the U22 did not stack with layer T3 (G24) as well as A22, manifesting a larger fluctuation of layer distance in 4–5.5 Å (see Table 1; Figure 6A). When A22 is replaced with U22, the side chain, i.e., the base part of this nucleotide, becomes smaller. This change causes the space of the binding pocket to grow larger and leaves more room for the ligand to move inside. Thus, configuration B occurred in the simulation, and this conversion from A to B was slow to reach an equilibrium. Configuration B showed a very different binding mode: A12-BzT-G13 and MQ-U22, and both two rings were straying from the coplanar state, and these binding modes were not so stable (see Supplementary Figure S6). The decrease in stacking interactions, the damage to the coplanar structure, and the steric hindrance around the PEG linker, altogether led to a lower binding free energy: 9.91 ± 0.43 kcal/mol (Table 2).

$$K_{d}^{}=\frac{\left[R\right]\left[L\right]}{\left[RL\right]}$$ (2)

**FIGURE 7:**
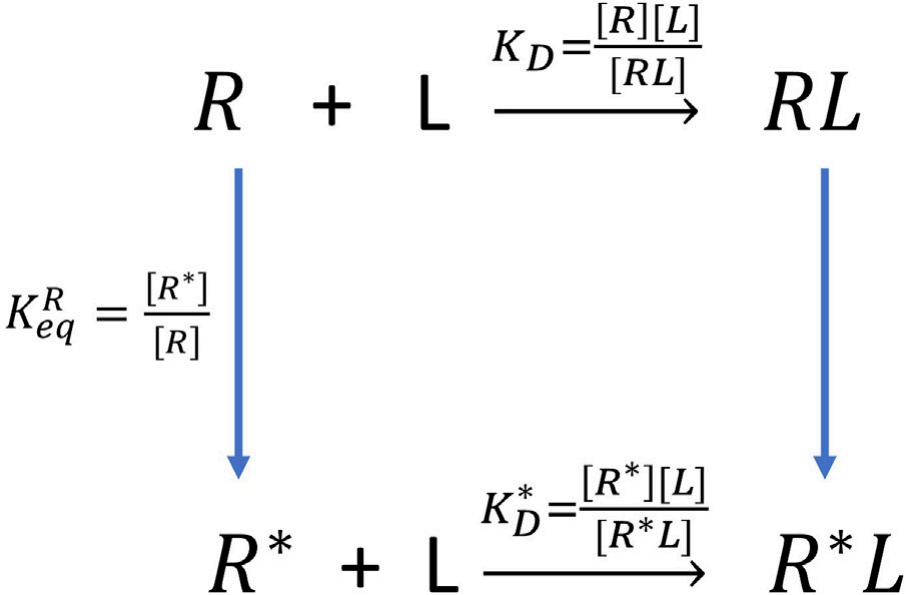
The thermodynamic cycle of double configurations for aptamer-fluorogen binding.

The experiment can only measure the apparent dissociation constant $K_{d}^{app}$ without knowing different binding modes or states (Eq. 2). When aptamer-fluorogen complexes possess two or even more configurations or binding modes, $K_{d}^{app}$ is a combined contribution from each configuration. However, computational simulations can unveil much more information, including atomistic structures of different configurations. The calculated free energy is for one specified configuration by using relative short (several ns) alchemical pathways simulations (see Figure 7). For example, it is possible to recover the $K_{d}^{app}$ by using Eq. 3 when there exist 2 separate binding configurations. The details to derive this equation can be found in **SI**.

$$\begin{array}{c} K_{d}^{app}=\frac{\left(\left[R\right]+\left[R^{\text{∗}}\right]\right)\left[L\right]}{\left[RL\right]+\left[RL^{\text{∗}}\right]}=\left(1+K_{eq}^{R}\right)\frac{K_{D}K_{D}^{\text{∗}}}{K_{D}+K_{eq}^{R}K_{D}^{\text{∗}}} \end{array}$$ (3)

[RL] and $\left[RL^{*}\right]$ are two different configurations of complexes, and they correspond to distinct configurations of RNA: [R] and $\left[R^{*}\right]$ . The alchemical pathways can only gradually transform [R] to [RL] ( $\left[R^{*}\right]$ to $\left[RL^{*}\right]$ ), and the free energy calculated under these situations can be translated into $K_{D}$ and $K_{D}^{*}$ respectively. However, $K_{eq}^{R}$ is unsolved. It denotes the transformation between two configurations of apo RNA itself, which could be evaluated by using pathway sampling such as umbrella sampling (US) (Torrie and Valleau, 1977; Kumar et al., 1992; Bartels, 2000). This could be very challenging as the free RNA aptamers without ligands are likely to be very dynamic and have no well-defined structures. Therefore, it is not surprising that RNA aptamer crystal structures all have ligand bound. Thus, we will leave it for future studies and here we only report the binding free energy for each state. Note that previously we took a similar approach to investigate apparent binding affinity for phosphate binding where the phosphate possesses multiple protonation states (Qi et al., 2018). Since the Mango-II-A22U_TO1-Biotin crystal structure is available and similar to that of A, it is reasonable to hypothesize that configuration A is dominant. Thus, the apparent binding free energy of Mango-II-A22U_TO1-Biotin from simulation should be closer to -11.07 kcal/mol (in the range of -11.07∼-9.91 kcal/mol).

**FIGURE 8:**
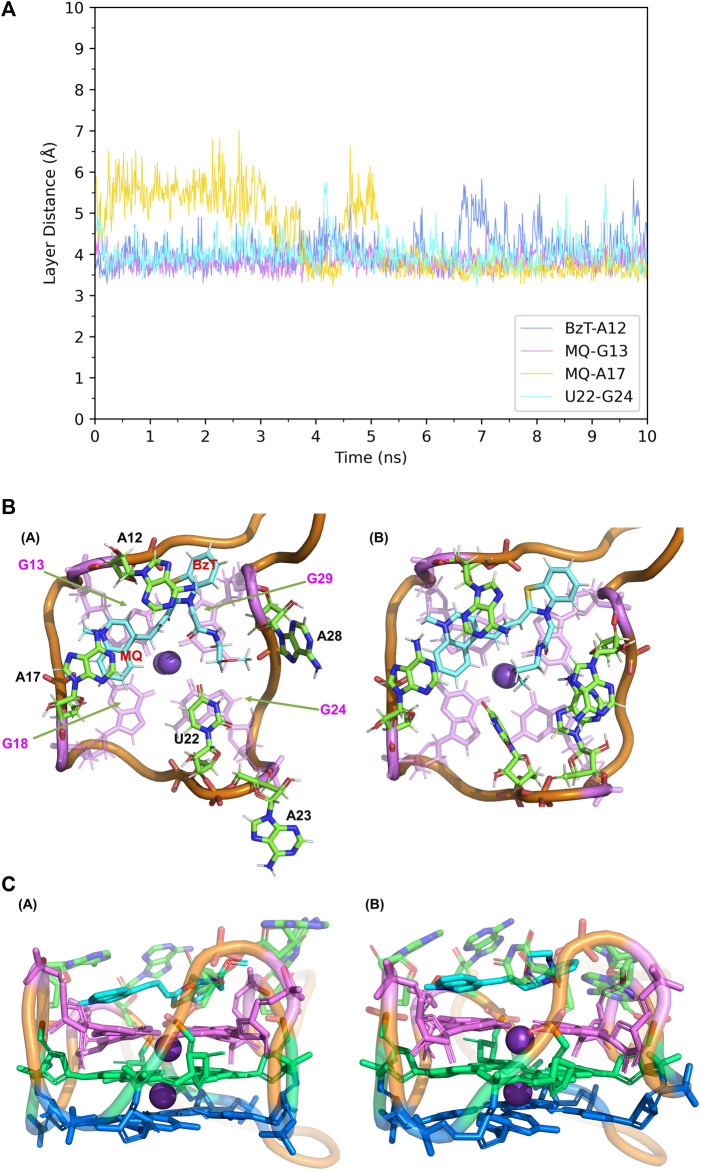
Structural analysis of MD simulation on Mango-II mutant (A22U) with TO3-Biotin. **(A)**: the trajectories of important structural parameters in the MD process. **(B)**: top views of the equilibrium structure from the MD process (both at 4 ns snapshot) (Left: configuration A; Right: configuration B. **(C)**: side views of the equilibrium structure.

When it comes to the simulation of Mango-II-A22U and TO3-Biotin, it was complicated as there is no known crystal structure for this complex. The Mango-II-A22U_TO1-Biotin complex (6C65) was utilized as a starting structure, with TO3-Biotin placed into the pocket following the same binding mode as that in Mango-II-A22U_TO1-Biotin. The 10 ns MD simulation led to two configurations in the study. In Figure 8; Table 1, the layer distances of stacking pairs BzT-A12, MQ-G13, MQ-A17, and U22-G24 of configuration A mostly stabilize around 3.8 Å after the 4 ns MD process. Compared with the structure of WT Mango-II_TO3-Biotin, the TO3-Biotin seemed to fit in the binding pocket of mutant even better than that of wild type, especially the U22 can hold a solid stacking with T3 layer (G24) in mutant while A22 stacking is interrupted by the PEG in the wild-type case. All of these observations account for the stronger binding free energy, which is -17.22 ± 0.38 kcal/mol from the calculation (Table 2). However, the structure of configuration B is not as stable as that of configuration A. From Table 1; Supplementary Figure S8, the stacking of BzT-A12 and MQ-A12 shows a large oscillation. It indicates that A12 is moving back and forth, holding different stacking periodically. At the same time, the stacking of MQ-G13 and MQ-A17 is relatively stable, but the distances are all larger than those from configuration A. All these structural data tell that this configuration has a more flexible core structure and the binding is not tight enough in the pocket. So, it is undoubtful to see the calculated free energy is -9.82 ± 0.39 kcal/mol (Table 2), which is far less than that of configuration A. Both simulation results seem to contradict the experimental observation that this mutant displayed a reduced affinity for TO3-Biotin, to -11.23 kcal/mol. As we explained above the apparent, this simulation started from a hypothetical structure and the apparent $K_{d}^{app}$ could arise from multiple states we did not completely sample in the simulations. Therefore, the simulation results presented here should be compared until further sampling or better structural information is available.

Experimentally (Trachman et al., 2018) it was suggested the A22U of Mango-II mutant showed an increased affinity for TO1-Biotin but decreased affinity for TO3. However, the increase of binding free energy for Mango-II-A22U_TO1-Biotin is almost minimal from Kd = 1.3 nM to 0.9 nM, which is only ∼0.2 kcal/mol difference in free energy equivalence. It means the steric hindrance of the PEG linker with the bases on sites 22, 23, and 28 does not significantly impact the binding. In addition, this linker is flexible and long without any side chains so that the space surrounding it is adequate for those bases to move freely. But the base on site 22 could control the volume of the binding pocket and create stereoselectivity as a result. There is no crystal structure or any further discussion on how TO3-Biotin could have weaker binding to this mutant. The experimental research on this mutant is far from completion and we will attempt additional experimental and computational studies in the future.

## Conclusion

In this work, we reported all-atom MD simulations of ligand binding in FLAPs-fluorogen complex using polarizable force field AMOEBA. The most important interactions in the binding arise from π−π stacking between two heterocycles (MQ, BzT) of the TO1-Biotin/TO3-Biotin and A12, A17, and T3 base layer of Mango-II. The binding free energy values calculated by AMOEBA are highly consistent with the experimental results for the wild-type Mango-II with its matching fluorogens (TO1-Biotin and TO3-Biotin), including the subtle preference among the two fluorogen molecules. In addition, the simulations unveil atomistic details about the structure and the binding modes of one mutant (A22U). We found that TO1-Biotin and TO3-Biotin indeed have similar binding affinities to wild-type Mango-II, and their structures are rather stable in a coplanar state. However, the simulations reveal the possibility that the mutant can transform into different configurations when forming complexes with ligands or in apo form. Additional work is needed to further understand the binding modes and configurations of the mutants.

RNA system is usually complicated to sample in computational simulation for its strong electric interactions, flexible backbones, and slow dynamics, which makes ligand binding in RNA study even more challenging. This work is the first attempt to simulate RNA ligand binding using advanced electrostatic and polarizable models. The results are encouraging but also demonstrate the need for experimental support (crystal structures).

We will continue to investigate the binding mechanisms underlying FLAPs-fluorogen complexes. The Mango-II, or other FLAPs such as Mango-III (Trachman et al., 2019) and Broccoli (Filonov et al., 2014), can be used to generate more mutants with the potential improvement in affinity and fluorescence. To explore the fluorescent properties, the QM/MM method (Senn and Thiel, 2009; Loco et al., 2016; Loco et al., 2017) involving AMOEBA will be a viable strategy. Also, the FLAPs can be specially designed to image non-nucleic acid molecules or metal ions by an allosteric mecahnism, in which the FLAP can bind non-nucleic acid targets and activate high fluorescence only after binding the specific fluorogen at first. There will be a wide range of application of aptamers in bio-sensing and bio-imaging, and molecular modeling can play a significant role in our understanding and designing the underlying molecular systems.

## Data Availability

The datasets presented in this study can be found in online repositories. The names of the repository/repositories and accession number(s) can be found in the article/Supplementary Material.
