# Supplementary material for: Computational study on the binding of Mango-II RNA aptamer and fluorogen using the polarizable force field AMOEBA
Source: Front Mol Biosci. 2022 Sep 2;9:946708. doi: 10.3389/fmolb.2022.946708 (PMC9478177; doi:10.3389/fmolb.2022.946708)
Supplement: Supplementary file 1 [file DataSheet1.pdf]

Supplementary Information

Computational Study on the Binding of Mango-II RNA Aptamer and Fluorogen Using the Polarizable Force Field AMOEBA

Xudong Yang[1], Chengwen Liu[1], Yu-An Kuo[1,2], Hsin-Chih Yeh[1,2], Pengyu Ren[1,*]

1. Department of Biomedical Engineering, The University of Texas at Austin, Austin, TX 78712, USA

2. Texas Materials Institute, University of Texas at Austin, Austin, TX 78712, USA

* Corresponding author. Email: pren@utexas.edu.

Contents

I. Tables 2

II. Figures 10

III. Deduction of equation...18

IV. QM and MM geometries of ligands 18

V. Parameters of ligands in Tinker format 24

(TO1-Biotin parameters: 24; TO3-Biotin parameters: 45)

I. Tables

Table S1. The details of the system used for simulation.

Complex	Number of K^{+} a)	Number of Cl^{-} b)	Range of restraint c)
Mango-II-WT TO1-Biotin	58	27	5.00 ~ 6.00
Mango-II-WT TO3-Biotin	58	27	5.00 ~ 6.00
Mango-II-A22U TO1-Biotin	57	27	5.00 ~ 6.00 (Config. A)<br>5.00 ~ 6.20 (Config. B)
Mango-II-A22U TO3-Biotin	57	27	5.50 ~ 6.80 (Config. A)<br>5.00 ~ 6.80 (Config. B)

a). K^{+} in solvation (The number of K^{+} inside the G-quadruplex is 2 and they are not counted here). b). Cl^{-} in solvation. c). The harmonic restraint will be removed only if the distance between COM of ligands and K2 (the K^{+} between T3 and T2 layers) is inside the range mentioned here.

Table S2. Statistics of the position of K^{+} in three-layer G-quadruplex during the equilibration.

Complex a)	T3-K2 b)	T2-K2 b)	T2-K1 b)	T1-K1 b)
Mango-II-WT	1.45±0.19	1.99±0.18	1.84±0.19	1.53±0.20
Mango-II-WT TO1-Biotin	1.29±0.17	2.12±0.17	1.68±0.19	1.69±0.20
Mango-II-WT TO3-Biotin	1.36±0.17	2.07±0.17	1.76±0.19	1.57±0.20
Mango-II-A22U TO1-Biotin (A)	1.44±0.21	2.01±0.20	1.85±0.21	1.50±0.21
Mango-II-A22U TO1-Biotin (B)	1.25±0.22	2.18±0.21	1.70±0.21	1.60±0.22
Mango-II-A22U TO3-Biotin (A)	1.39±0.19	2.07±0.19	1.82±0.24	1.56±0.23
Mango-II-A22U TO3-Biotin (B)	1.22±0.19	2.15±0.18	1.71±0.18	1.63±0.19

a). (A)/(B) mean the configuration A or B. b). The specific definition of the abbreviations can be found in Figure 1. The data has been shown in the format as: Average ± standard deviation (Unit: Å).

Table S3. Statistics of the position of K^+ in three-layer G-quadruplex during the FEP alchemical calculation (126ns).

Complex[a)]	T3-K2[b)]	T2-K2[b)]	T2-K1[b)]	T1-K1[b)]
Mango-II-WT TO1-Biotin	1.16±0.37	2.32±0.23	1.55±0.22	1.85±0.22
Mango-II-WT TO3-Biotin	1.23±0.20	2.20±0.20	1.68±0.08	1.66±0.21
Mango-II-A22U TO1-Biotin (A)	1.35±0.23	2.08±0.22	1.80±0.21	1.55±0.22
Mango-II-A22U TO1-Biotin (B)	1.13±0.21	2.31±0.21	1.59±0.21	1.71±0.22
Mango-II-A22U TO3-Biotin (A)	1.31±0.20	2.14±0.20	1.75±0.22	1.62±0.22
Mango-II-A22U TO3-Biotin (B)	1.14±0.27	2.41±0.71	1.66±0.23	1.71±0.24

a). (A)/(B) mean the configuration A or B. b). The specific definition of the abbreviations can be found in Figure 1. The data has been shown in the format as: Average ± standard deviation (Unit: Å).

Table S4. The alchemical states used for the calculation of free energy

Index of state	λ_{vdw}	λ_{ele}	Force constant of harmonic restraint k_R (kcal/(mol·Å^2))
1	0.00	0.00	15
2	0.50	0.00	15
3	0.55	0.00	15
4	0.60	0.00	15
5	0.62	0.00	15
6	0.65	0.00	15
7	0.70	0.00	15
8	0.75	0.00	15
9	0.80	0.00	15
10	0.90	0.00	15
11	1.00	0.00	15
12	1.00	0.10	15
13	1.00	0.20	15
14	1.00	0.30	15
15	1.00	0.40	15
16	1.00	0.50	15
17	1.00	0.60	10
18	1.00	0.70	5

19	1.00	0.80	0
20	1.00	0.90	0
21	1.00	1.00	0

Table S5. Statistics of the interstage free energy change in the annihilation of TO1-Biotin from wild-type Mango-II_TO1-Biotin complex. (The states here are represented by corresponding indices mentioned in Table S4. Unit: kcal/mol)

State change	ΔG	RMSD
1 to 2	5.106	0.055
2 to 3	7.9549	0.0478
3 to 4	13.9947	0.1602
4 to 5	4.2996	0.1079
5 to 6	3.1671	0.078
6 to 7	2.1343	0.1204
7 to 8	-0.2994	0.0681
8 to 9	-2.4445	0.0627
9 to 10	-11.9338	0.0948
10 to 11	-24.5045	0.1109
11 to 12	-0.7262	0.0375
12 to 13	-1.9454	0.0405
13 to 14	-3.2742	0.0384
14 to 15	-4.7055	0.0438
15 to 16	-6.0603	0.037
16 to 17	-7.5918	0.0477
17 to 18	-9.6083	0.0483
18 to 19	-11.9364	0.0597
19 to 20	-14.6174	0.0572
20 to 21	-17.5225	0.0693
Restrain Correction	0.6913	

Table S6. Statistics of the interstage free energy change in the annihilation of TO1-Biotin from the configuration A of Mango-II-A22U_TO1-Biotin complex. (The states here are represented by corresponding indices mentioned in Table S4. Unit: kcal/mol)

State change	ΔG	RMSD
1 to 2	1.9359	0.0091
2 to 3	3.7139	0.0131
3 to 4	7.9712	0.0313
4 to 5	4.7967	0.0217
5 to 6	8.5257	0.0548
6 to 7	12.2082	0.2782

7 to 8	7.0319	0.1392
8 to 9	3.8106	0.0825
9 to 10	-0.2502	0.1902
10 to 11	-10.7289	0.0836
11 to 12	4.0386	0.0315
12 to 13	2.3296	0.0332
13 to 14	0.479	0.0355
14 to 15	-1.5732	0.0383
15 to 16	-4.0227	0.0461
16 to 17	-7.2265	0.0561
17 to 18	-11.725	0.0818
18 to 19	-18.9037	0.1339
19 to 20	-31.0238	0.289
20 to 21	-51.0601	0.5887
Restrain Correction	0.6913	

Table S7. Statistics of the interstage free energy change in the annihilation of TO1-Biotin from the configuration B of Mango-II-A22U_TO1-Biotin complex. (The states here are represented by corresponding indices mentioned in Table S4. Unit: kcal/mol)

State change	ΔG	RMSD
1 to 2	5.1753	0.0526
2 to 3	7.8988	0.0557
3 to 4	13.4672	0.165
4 to 5	3.8369	0.1007
5 to 6	2.287	0.0834
6 to 7	1.1154	0.0769
7 to 8	-0.643	0.0682
8 to 9	-2.7028	0.0547
9 to 10	-13.7957	0.0625
10 to 11	-23.7263	0.0776
11 to 12	-0.0529	0.0427
12 to 13	-1.3402	0.052
13 to 14	-2.4986	0.0449
14 to 15	-3.9358	0.0442
15 to 16	-5.4716	0.0461
16 to 17	-7.2293	0.0479
17 to 18	-9.0417	0.0478
18 to 19	-11.3286	0.0641
19 to 20	-13.7957	0.0625
20 to 21	-16.6278	0.064
Restrain	0.5875	

Correction		

Table S8. Statistics of the interstage free energy change in the solvation of TO1-Biotin. (The states here are represented by corresponding indices mentioned in Table S4. Unit: kcal/mol)

State change	ΔG	RMSD
1 to 2	3.2544	0.0182
2 to 3	5.994	0.0317
3 to 4	10.5201	0.1414
4 to 5	3.1783	0.0744
5 to 6	2.4685	0.0716
6 to 7	1.744	0.0795
7 to 8	0.1795	0.0591
8 to 9	-1.1372	0.0442
9 to 10	-6.4516	0.0795
10 to 11	-14.4784	0.0888
11 to 12	-0.0249	0.0445
12 to 13	-1.3465	0.0472
13 to 14	-2.7753	0.0443
14 to 15	-4.1272	0.0458
15 to 16	-5.6486	0.0483
16 to 17	-7.3282	0.0492
17 to 18	-9.2552	0.055
18 to 19	-11.434	0.0576
19 to 20	-13.9842	0.0657
20 to 21	-17.2574	0.0798

Table S9. Statistics of the interstage free energy change in the annihilation of TO3-Biotin from wild-type Mango-II_TO3-Biotin complex. (The states here are represented by corresponding indices mentioned in Table S4. Unit: kcal/mol)

State change	ΔG	RMSD
1 to 2	5.6047	0.0425
2 to 3	8.6706	0.0687
3 to 4	14.3202	0.156
4 to 5	4.8909	0.124
5 to 6	3.4468	0.0857
6 to 7	2.5624	0.1083
7 to 8	-0.021	0.0903
8 to 9	-2.674	0.0628
9 to 10	-13.6573	0.1419
10 to 11	-27.1263	0.0757
11 to 12	-0.2698	0.0366
12 to 13	-0.9386	0.0338

13 to 14	-1.8874	0.041
14 to 15	-2.9836	0.0353
15 to 16	-4.3279	0.0499
16 to 17	-5.7511	0.0381
17 to 18	-6.8371	0.0413
18 to 19	-8.1012	0.0421
19 to 20	-9.7248	0.0488
20 to 21	-11.7511	0.0546
Restrain Correction	0.6913	

Table S10. Statistics of the interstage free energy change in the annihilation of TO3-Biotin from the configuration A of Mango-II-A22U_TO3-Biotin complex. (The states here are represented by corresponding indices mentioned in Table S4. Unit: kcal/mol)

State change	ΔG	RMSD
1 to 2	4.8775	0.0534
2 to 3	7.6464	0.0484
3 to 4	13.6055	0.1509
4 to 5	4.2185	0.0982
5 to 6	3.0746	0.0899
6 to 7	1.7182	0.1039
7 to 8	-0.7575	0.0739
8 to 9	-2.839	0.0488
9 to 10	-12.3229	0.1074
10 to 11	-26.3573	0.1284
11 to 12	-0.5565	0.0413
12 to 13	-1.6898	0.0366
13 to 14	-2.6647	0.0356
14 to 15	-3.6047	0.0372
15 to 16	-4.642	0.0379
16 to 17	-5.7927	0.0421
17 to 18	-6.9909	0.04
18 to 19	-8.1808	0.0436
19 to 20	-9.6719	0.0561
20 to 21	-11.9472	0.0578
Restrain Correction	0.4398	

Table S11. Statistics of the interstage free energy change in the annihilation of TO3-Biotin from the configuration B of Mango-II-A22U_TO3-Biotin complex. (The states here are represented by corresponding indices mentioned in Table S4. Unit: kcal/mol)

State change	ΔG	RMSD
1 to 2	5.2736	0.0366
2 to 3	8.2494	0.0449
3 to 4	14.2153	0.1186
4 to 5	5.3124	0.0712
5 to 6	4.1662	0.0793
6 to 7	2.6974	0.1183
7 to 8	-0.3375	0.0542
8 to 9	-2.4511	0.0446
9 to 10	-11.8772	0.0805
10 to 11	-25.1459	0.1092
11 to 12	-0.4573	0.0275
12 to 13	-1.3929	0.0346
13 to 14	-2.3161	0.0262
14 to 15	-3.3461	0.0367
15 to 16	-4.7802	0.0321
16 to 17	-5.9471	0.0313
17 to 18	-7.1036	0.0313
18 to 19	-8.2823	0.0335
19 to 20	-9.8166	0.0378
20 to 21	-11.6017	0.0396
Restrain Correction	0.3294	

Table S12. Statistics of the interstage free energy change in the solvation of TO3-Biotin. (The states here are represented by corresponding indices mentioned in Table S4. Unit: kcal/mol)

State change	ΔG	RMSD
1 to 2	3.2647	0.0162
2 to 3	6.0518	0.029
3 to 4	10.4509	0.1559
4 to 5	2.8574	0.0603
5 to 6	2.4299	0.0624
6 to 7	1.8998	0.0805
7 to 8	0.1607	0.0529
8 to 9	-1.1475	0.0404
9 to 10	-6.3288	0.069
10 to 11	-13.355	0.061
11 to 12	0.1322	0.0398
12 to 13	-0.7653	0.0401
13 to 14	-1.7234	0.0399
14 to 15	-2.7606	0.0407
15 to 16	-3.9498	0.0425

16 to 17	-5.1851	0.0435
17 to 18	-6.5626	0.0448
18 to 19	-8.1086	0.0492
19 to 20	-9.9617	0.0537
20 to 21	-12.1937	0.058

II. Figures

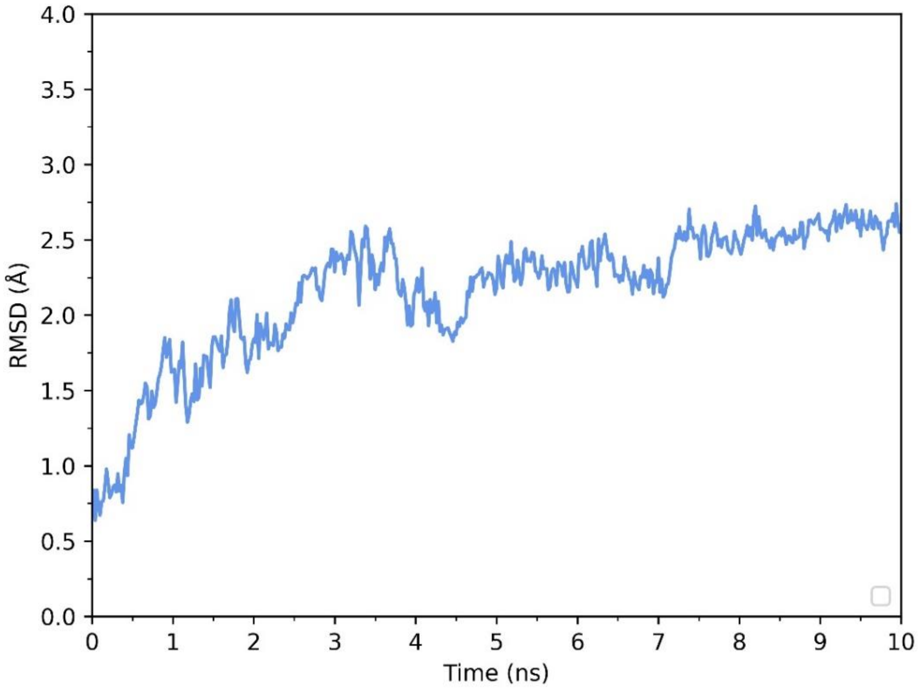

Figure S1. RMSD trajectory of core G-quadruplex (the heavy atoms from bases of 10~29) from the MD simulation of single wild type Mango-II.

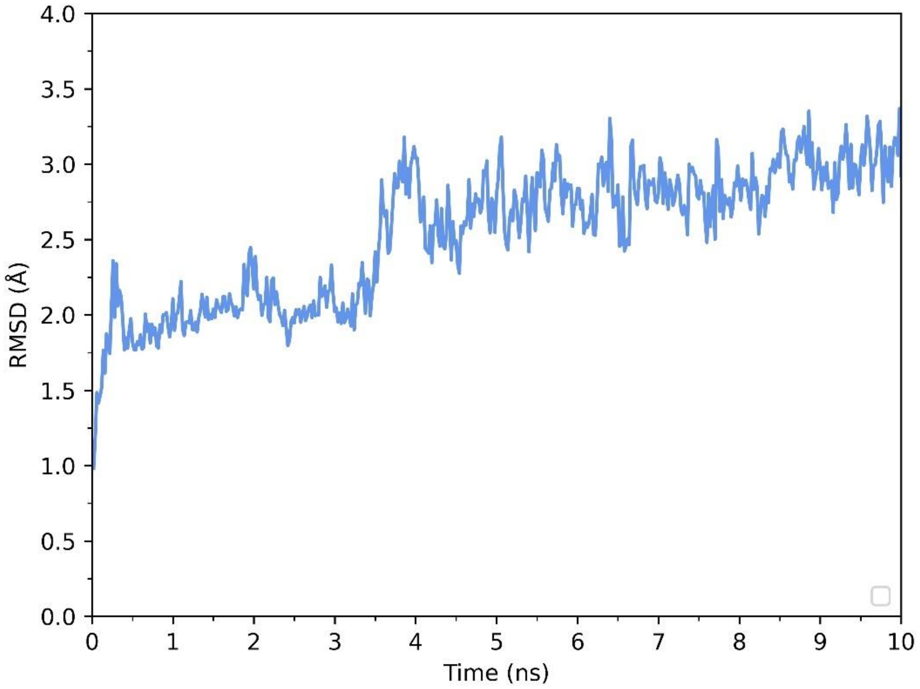

Figure S2. RMSD trajectory of core G-quadruplex (the heavy atoms from bases of 10~29) from the MD simulation of single wild type Mango-II without polarization.

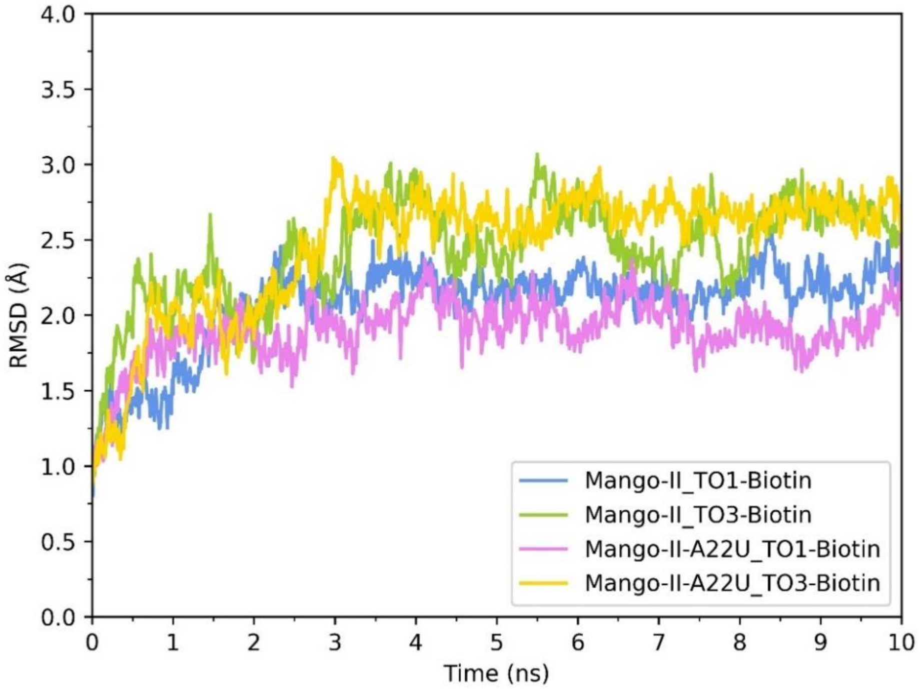

Figure S3. RMSD trajectories of core G-quadruplex (the heavy atoms from bases of 10~29) from the MD simulations on 4 aptamer-fluorogen complexes (Mango-II-A22U complexes choose the configuration A)

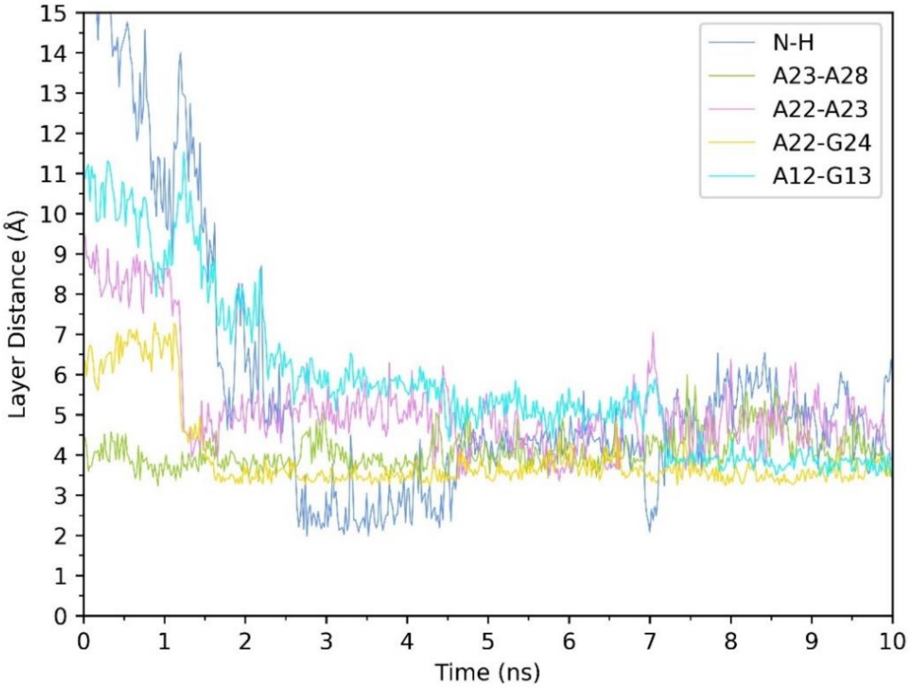

Figure S4. The trajectories of important structural parameters in MD simulation (N-H denotes the distance of N of A12 and the H on G24, see the 4ns snapshot of the **Figure**

3).

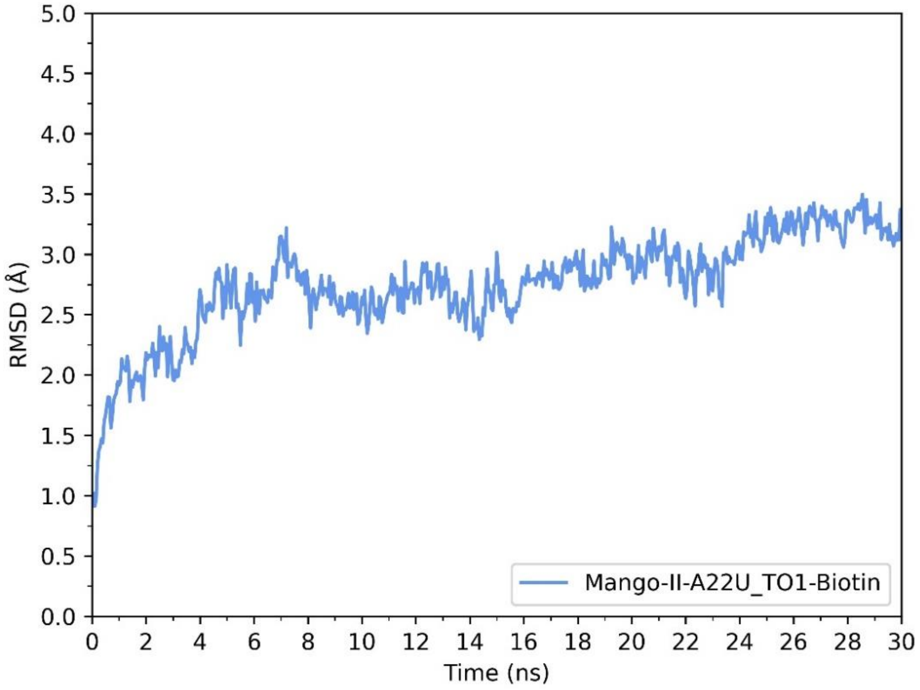

Figure S5. RMSD trajectories of core G-quadruplex (the heavy atoms from bases of 10~29) from the MD simulation on the configuration B of Mango-II-A22U_TO1-Biotin.

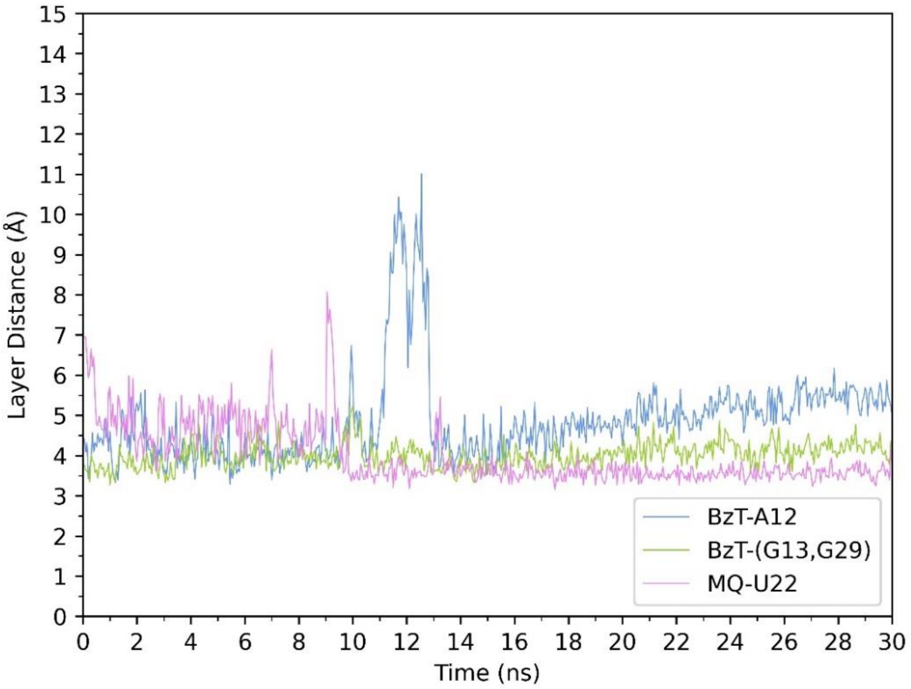

Figure S6. Trajectories of important structural parameters from the MD simulation on the configuration B of Mango-II-A22U_TO1-Biotin.

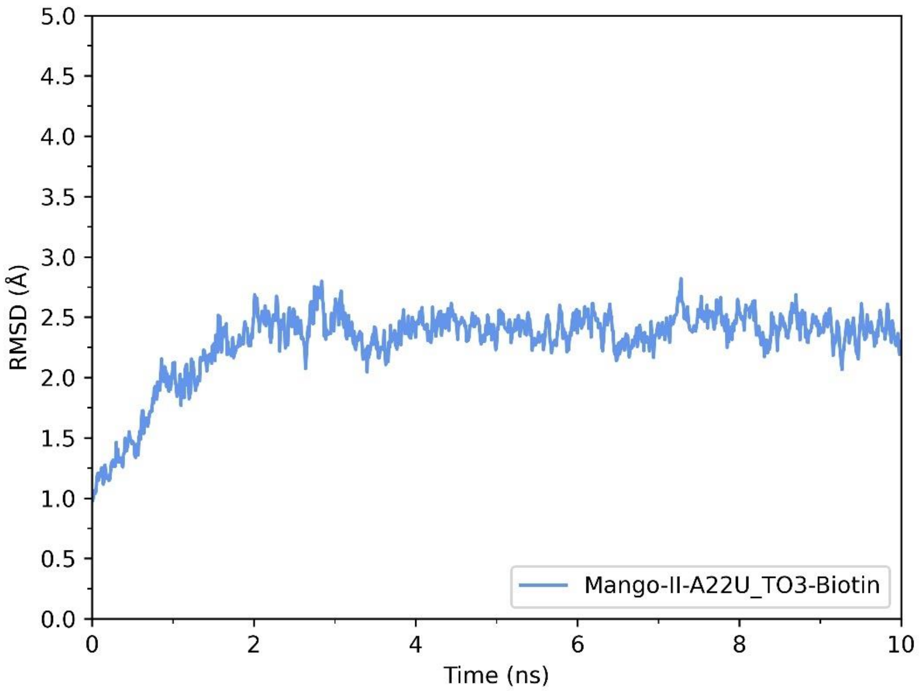

Figure S7. RMSD trajectories of core G-quadruplex (the heavy atoms from bases of 10~29) from the MD simulation on the configuration B of Mango-II-A22U_TO3-Biotin.

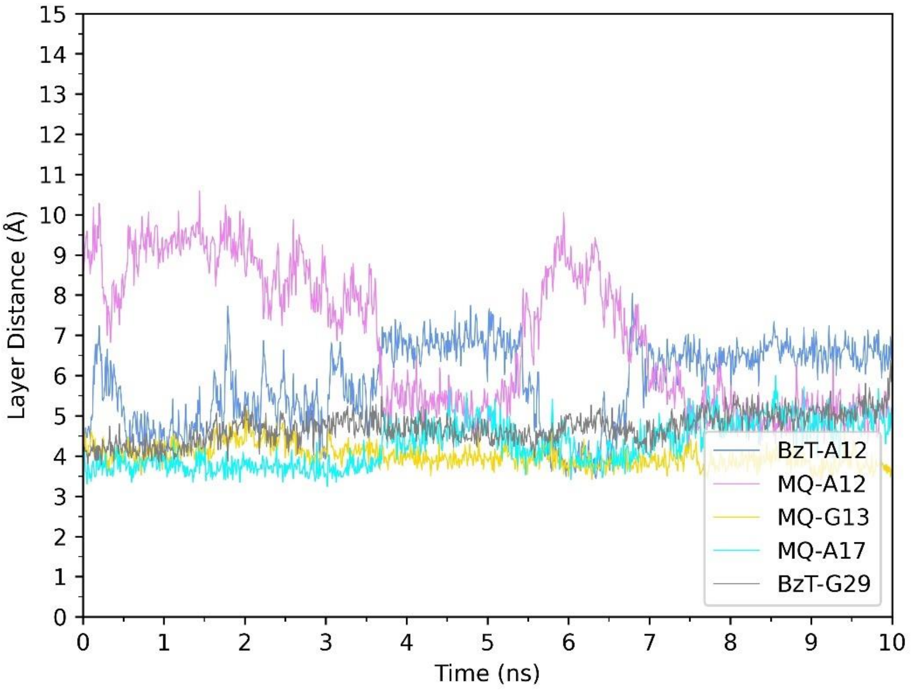

Figure S8. Trajectories of important structural parameters from the MD simulation on the configuration B of Mango-II-A22U_TO3-Biotin.

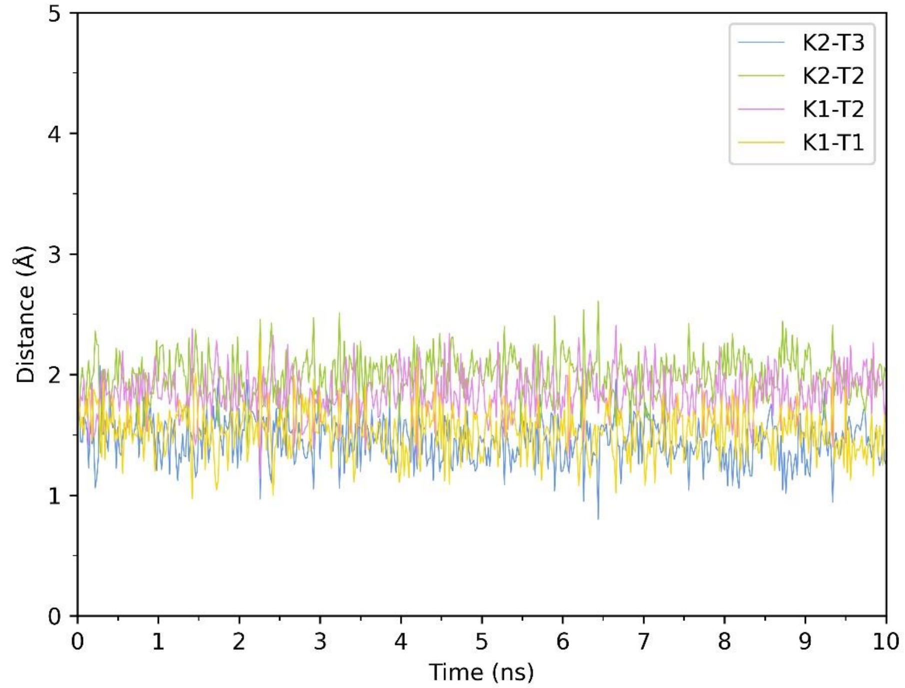

Figure S9. Statistics of the position of K^+ in three-layer G-quadruplex of wild-type Mango-II during the 10ns equilibration.

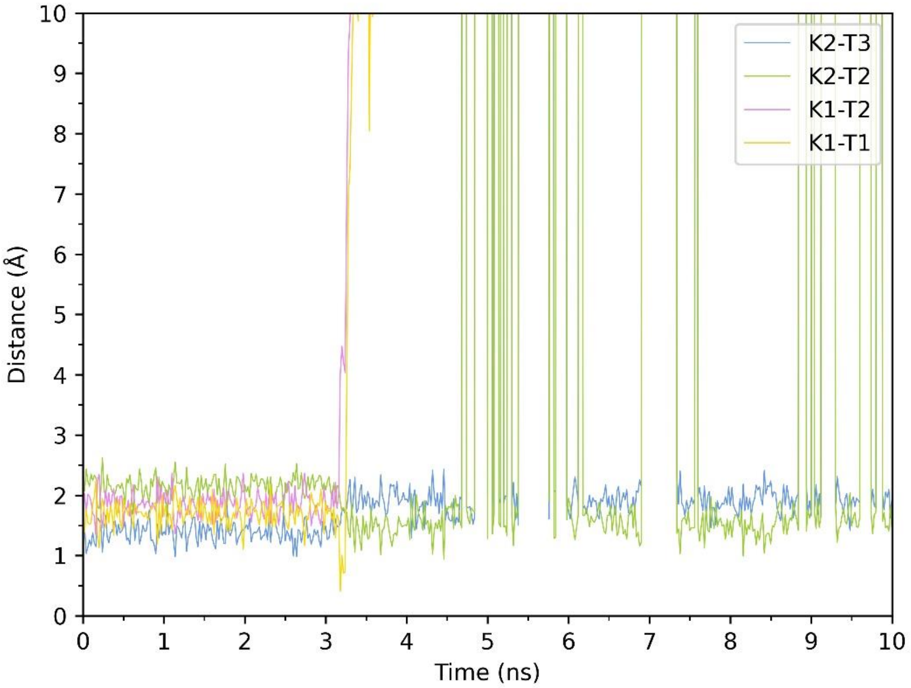

Figure S10. Statistics of the position of K^+ in three-layer G-quadruplex of wild-type Mango-II during the 10ns equilibration with no polarization.

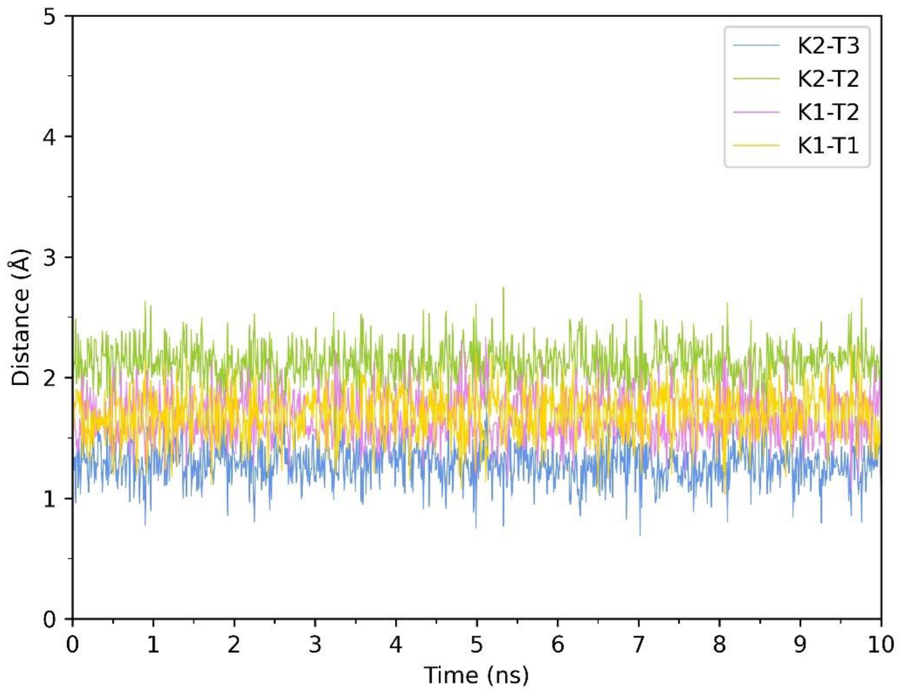

Figure S11. Statistics of the position of K⁺ in three-layer G-quadruplex of wild-type Mango-II_TO1-Biotin complex during the 10ns equilibration.

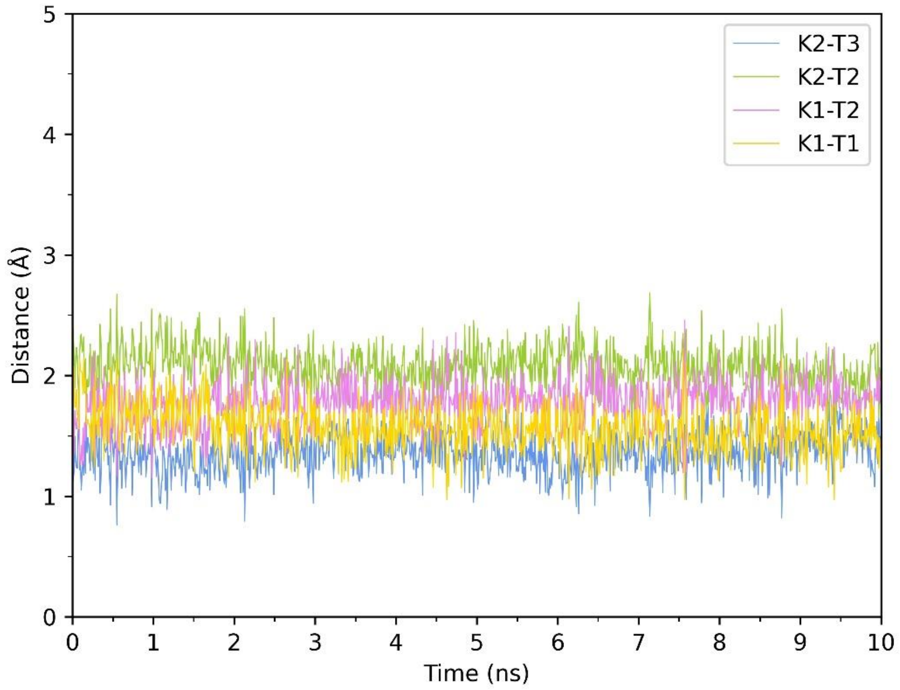

Figure S12. Statistics of the position of K⁺ in three-layer G-quadruplex of wild-type Mango-II_TO3-Biotin complex during the 10ns equilibration.

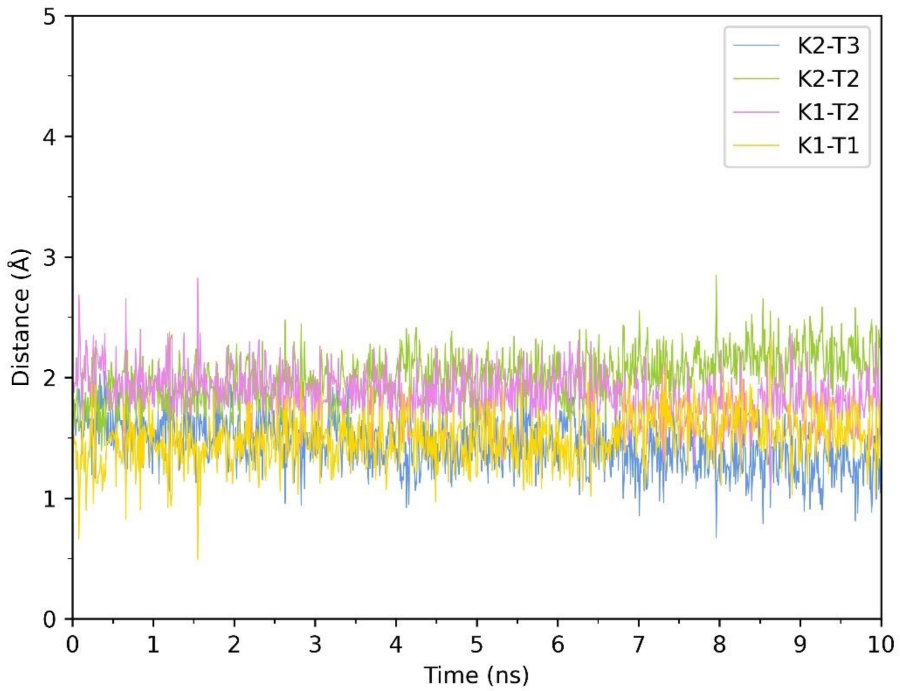

Figure S13. Statistics of the position of K+ in three-layer G-quadruplex from configuration A of Mango-II-A22U_TO1-Biotin complex during the 10ns equilibration.

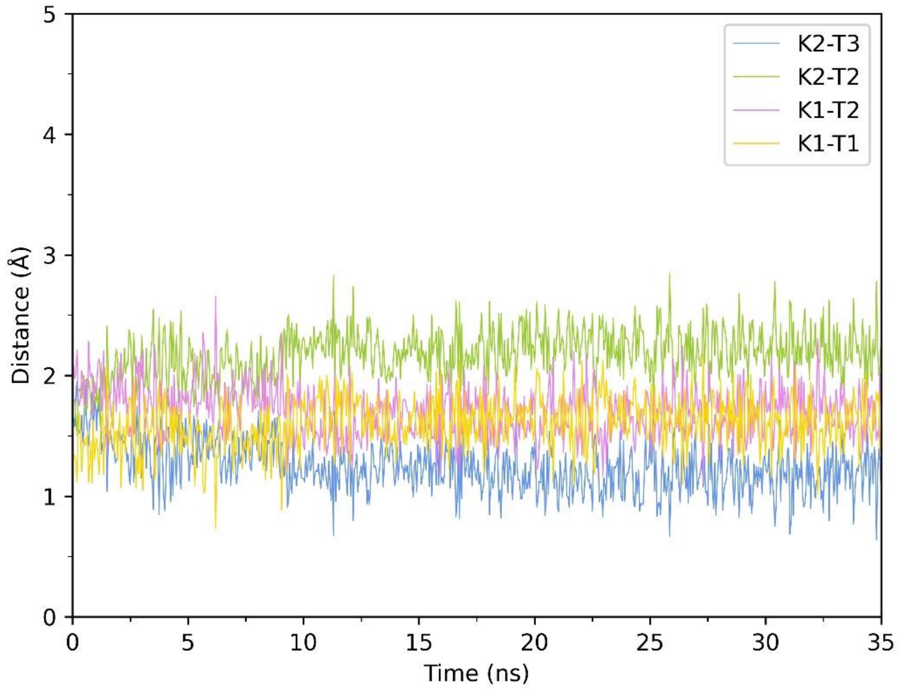

Figure S14. Statistics of the position of K+ in three-layer G-quadruplex from configuration B of Mango-II-A22U_TO1-Biotin complex during the 35ns equilibration.

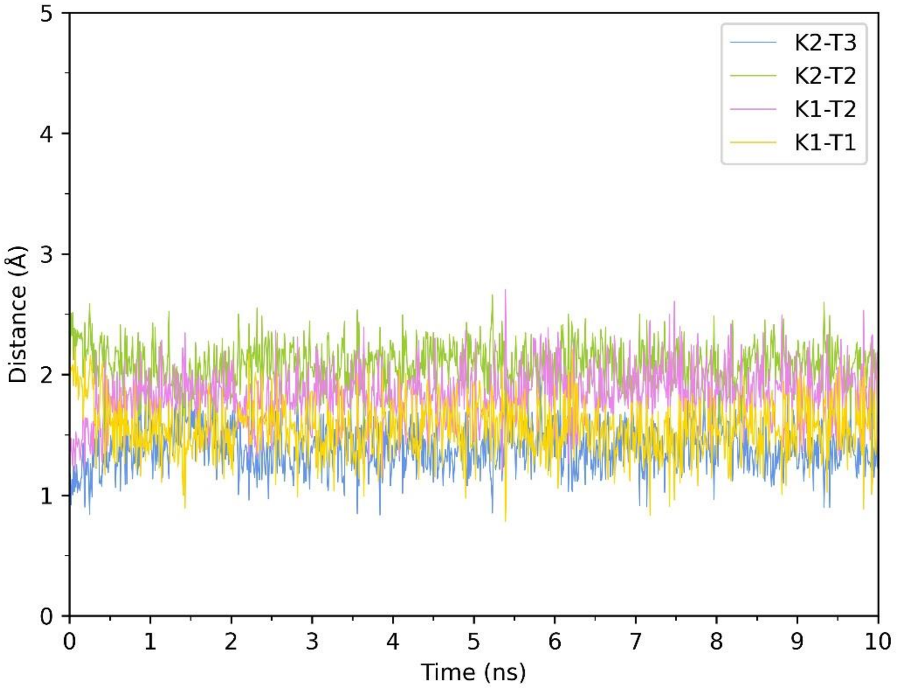

Figure S15. Statistics of the position of K^+ in three-layer G-quadruplex from configuration A of Mango-II-A22U_TO3-Biotin complex during the 10ns equilibration.

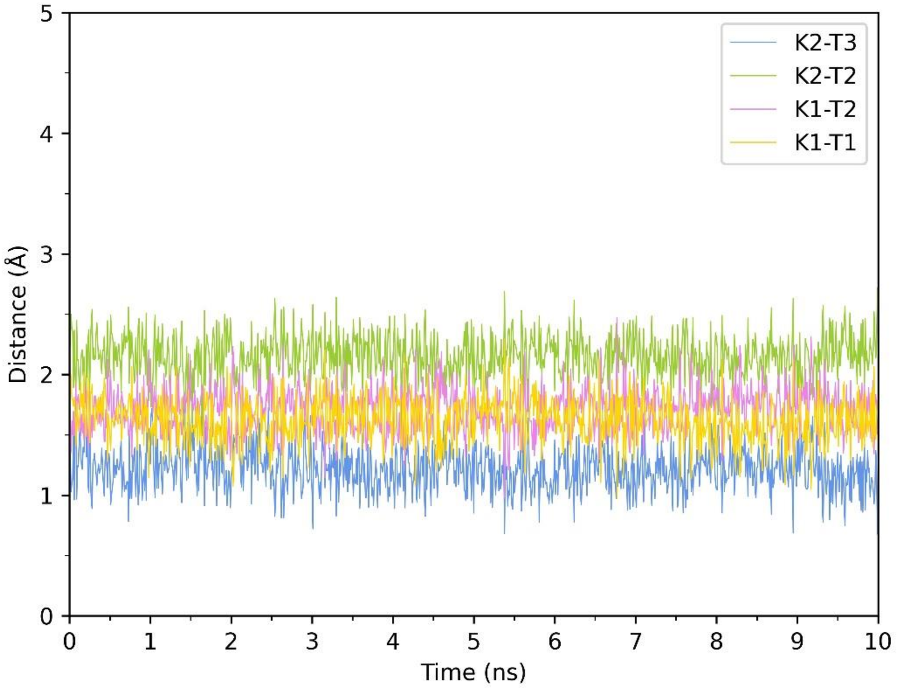

Figure S16. Statistics of the position of K^+ in three-layer G-quadruplex from configuration B of Mango-II-A22U_TO3-Biotin complex during the 10ns equilibration.

III. Deduction of Equation

The deduction of the Equation 3

$$K_D = \frac{[R][L]}{[RL]} \Rightarrow [RL] = \frac{[R][L]}{K_D}$$

$$K_D^* = \frac{[R^*][L]}{[R^*L]} \Rightarrow [R^*L] = \frac{[R^*][L]}{K_D^*}$$

$$K_{eq}^R = \frac{[R^*]}{[R]}$$

$$K_D^{app} = \frac{([R) + [R^*]][L]}{[RL] + [R^*L]} = \frac{[R] + [R^*]}{\frac{[R]}{K_D} + \frac{[R^*]}{K_D^*}} = \frac{[R](1 + K_{eq}^R)}{[R](\frac{1}{K_D} + \frac{K_{eq}^R}{K_D^*})} = K_D K_D^* \frac{1 + K_{eq}^R}{K_D^* + K_D K_{eq}^R}$$

IV. QM and AMOEBA geometries of ligands

QM optimized geometry of TO1-Biotin (charge: 2)

1 C -0.998818978622 -0.185918954587 0.530002383579
2 C -1.963549127011 0.196160926568 1.384379580966
3 C -3.391955323401 -0.058349347291 1.111113270404
4 C 6.030877920694 -0.341452952144 -1.324460246607
5 C 5.593902707390 1.080726916052 0.573956937976
6 C 4.523832945947 2.057786265438 1.003439761675
7 C 2.129059490923 1.907350616975 1.412620725232
8 C 0.795574556665 1.226506101804 1.243582096525
9 C -4.068154225733 -0.976661632262 1.896224011781
10 C -5.423936666970 -1.220622235116 1.680140424820
11 C -7.546279688641 -0.878673071709 0.557129231319
12 C -5.503019399443 0.375511295886 -0.077953092555
13 C -6.243362003610 1.068917874901 -1.060185666615
14 C -5.613222638703 2.018393084701 -1.836638341604
15 C -4.247653293261 2.321212279697 -1.645700795884
16 C -3.507597256710 1.652796516714 -0.694851385303
17 C -4.112910774053 0.667768418988 0.124175110207
18 C 0.465168125424 -1.680521912260 -0.759241395365
19 C 1.039212024108 -2.751986414952 -1.433203879320
20 C 2.296974138269 -3.196421078840 -1.018527547735
21 C 2.951893463816 -2.608122850747 0.071447383863
22 C 2.392327622506 -1.522988083458 0.742830908361
23 C 1.175954817529 -1.035790613801 0.261038186022

24 N 3.271657474525 1.293987324841 1.055033824462
25 N 0.415223497784 0.058874797601 0.787028901250
26 N -6.106676153516 -0.568901230270 0.731443672867
27 O 5.165984735015 0.617204736258 -0.702126483613
28 O 2.031306721980 3.025090372217 1.933429962915
29 S -1.161879916087 -1.024934360994 -1.005429868065
30 H -1.695629645958 0.643254410077 2.342281374778
31 H 5.557137259178 -0.587144515203 -2.275636450761
32 H 6.085322836019 -1.254905325636 -0.714192529726
33 H 6.555594381759 1.601626308376 0.523268330776
34 H 5.688552030259 0.249117758084 1.288471575092
35 H 4.442338265918 2.877065654768 0.282564204836
36 H 4.725476798909 2.476717322714 1.992042581249
37 H -3.559861921018 -1.534603856150 2.677030348192
38 H -5.976610877250 -1.942415340474 2.271932379305
39 H -7.712320693219 -1.253208388386 -0.452818519662
40 H -8.130219696171 0.023924940423 0.736623138506
41 H -7.822492327652 -1.642509497515 1.280515404664
42 H -7.294624701779 0.863852184920 -1.223416961318
43 H -6.182824147914 2.551752022993 -2.592375515326
44 H -3.777489130147 3.083754388763 -2.260003541623
45 H -2.463580410836 1.905725940087 -0.541177980702
46 H 0.511169065439 -3.254772865339 -2.238577782110
47 H 2.755320802413 -4.039278509588 -1.527990237084
48 H 3.894905223239 -3.018589997482 0.419605895360
49 H 2.866809167974 -1.100580599155 1.622249665742
50 H 3.245204255295 0.567263006659 0.340245286686
51 C 7.445466788240 0.145418672211 -1.588769726362
52 H 7.961804187137 0.426327778669 -0.663131660376
53 H 7.413153069526 1.024003508715 -2.244659209760
54 O 8.082250724926 -0.970639454572 -2.198836972719
55 H 8.801452445795 -0.648618862665 -2.766357568423
56 H -0.012930712016 1.908846854731 1.494663695102

AMOEBA optimized geometry of TO1-Biotin (charge: 2) (.txyz file)

56
1 C -1.10076903 -0.40865457 0.54317338 419 2 25
29
2 C -2.02702691 0.22803889 1.32923273 423 1 3
30
3 C -3.50040049 0.05103561 1.12483467 415 2 9
17
4 C 6.45961786 0.21539982 -1.46881474 401 27 31
32 51
5 C 5.79775227 1.05530202 0.74974962 404 6 27

```
33    34
      6   C      4.63802924     1.89320884     1.32178825    403       5     24
35    36
      7   C      2.17668831     1.63831895     1.39318463    408       8     24
28
      8   C      0.84871034     0.90921279     1.20236044    411       7     25
56
      9   C     -4.22660615    -0.53695857     2.15439343    414       3     10
37
     10   C     -5.60515509    -0.71734158     2.03294725    410       9     26
38
     11   C     -7.75844640    -0.52885153     0.88115041    405      26     39
40    41
     12   C     -5.65209674     0.43950944    -0.07039131    409      13     17
26
     13   C     -6.38345650     1.00309565    -1.17998759    413      12     14
42
     14   C     -5.70956634     1.72544669    -2.15956417    417      13     15
43
     15   C     -4.31282011     1.92811808    -2.07746233    424      14     16
44
     16   C     -3.58411662     1.40240407    -1.01741900    416      15     17
45
     17   C     -4.22892762     0.62839686     0.01335856    412       3     12
16
     18   C      0.16411873    -2.16513183    -0.66796708    425      19     23
29
     19   C      0.62700694    -3.31285663    -1.32941925    420      18     20
46
     20   C      1.97120375    -3.70020732    -1.16514002    427      19     21
47
     21   C      2.82771339    -2.96161269    -0.32858160    421      20     22
48
     22   C      2.37254632    -1.81958230     0.34871095    426      21     23
49
     23   C      1.03241534    -1.39280097     0.13062391    418      18     22
25
     24   N      3.37882954     1.15336406     1.00995339    406       6      7
50
     25   N      0.37333260    -0.24708158     0.71578948    422       1      8
23
     26   N     -6.29195445    -0.27365785     0.93781050    407      10     11
12
     27   O      5.51836441     0.96914657    -0.65818233    429       4      5
```

28	O	2.01375266	2.70388099	2.00897340	437	7			
29	S	-1.45874765	-1.53451283	-0.77899099	430	1	18		
30	H	-1.72495490	0.87417727	2.15832223	442	2			
31	H	6.07785826	0.23899936	-2.50256569	432	4			
32	H	6.45535087	-0.83075356	-1.11919331	432	4			
33	H	6.74888445	1.57442989	0.95511022	433	5			
34	H	5.85707870	0.06776170	1.23868831	433	5			
35	H	4.60458534	2.89091746	0.87466088	434	6			
36	H	4.73683327	2.01269197	2.40412352	434	6			
37	H	-3.75558059	-0.88526068	3.07745305	440	9			
38	H	-6.17496379	-1.21081672	2.81343897	438	10			
39	H	-7.98452201	-0.96264989	-0.09457557	435	11			
40	H	-8.25822472	0.42859707	1.04396880	435	11			
41	H	-8.03310848	-1.24062986	1.66242463	435	11			
42	H	-7.45582135	0.88827216	-1.27382174	439	13			
43	H	-6.27691631	2.13897124	-2.99575775	445	14			
44	H	-3.81407384	2.50973580	-2.85531865	446	15			
45	H	-2.51056640	1.59049138	-0.96187569	444	16			
46	H	-0.02961499	-3.90658939	-1.96925354	448	19			
47	H	2.34231194	-4.58717814	-1.68300056	447	20			
48	H	3.85839094	-3.29647795	-0.19220782	449	21			
49	H	3.02577291	-1.32735208	1.05456218	441	22			
50	H	3.45354876	0.45815539	0.27347602	436	24			
51	C	7.91730448	0.71999285	-1.48900302	402	4	52	53	54
52	H	8.36228198	0.70864112	-0.48200142	431	51			
53	H	7.97423625	1.75406487	-1.86503375	431	51			
54	O	8.58676792	-0.19623854	-2.34931460	428	51	55		
55	H	9.35146573	0.24948475	-2.75167873	450	54			
56	H	0.09898998	1.61226046	1.55650241	443	8			

QM optimized geometry of TO3-Biotin (charge: 2)

1 C -8.311832562926 -0.920443135638 0.411793438441
2 C -6.413746683550 0.586126895554 0.497733249872
3 C -5.054484302805 0.879403422247 0.504547763197
4 C -4.102756140989 -0.133684115521 0.470238175842
5 C -2.709908274110 0.270465310889 0.293797813382
6 C -1.789896029170 -0.416396186818 -0.441960287481
7 C -0.403296225598 -0.093846898446 -0.653944742540
8 C 0.320394038028 0.965820508342 -0.178433039024
9 C 1.582854891239 2.372581817101 1.404630379396
10 C 2.070642274651 3.207657641845 2.407507322838
11 C 3.431339071480 3.495708800184 2.424083050256
12 C 4.281628576292 2.993937060812 1.426573403026
13 C 3.802254741639 2.173243550669 0.414647590027

14 C 2.447354715656 1.810342119789 0.452165171328
15 C 2.105547291625 0.645253712069 -1.726849283006
16 C -4.562326263008 -1.484692418613 0.549849672882
17 C -3.693758521491 -2.590553405513 0.728271443411
18 C -4.189028395935 -3.875696741877 0.781636549834
19 C -5.572380545341 -4.118607685420 0.658405351525
20 C -6.459928712717 -3.070800723521 0.540676585416
21 C -5.973388260787 -1.745182834843 0.514665986099
22 N -6.852037817188 -0.678158081129 0.469603127199
23 N 1.718862386098 1.082665610826 -0.532892483302
24 S -0.114695261309 2.068147505540 1.094489508001
25 H -8.545422810865 -1.490156253517 -0.487565124665
26 H -8.816845040473 0.042197509475 0.374558812633
27 H -8.623365495669 -1.462998352466 1.304390667661
28 H -7.167980406464 1.365602456002 0.486557903993
29 H -4.759021089910 1.924318325971 0.480594633432
30 H -2.467368782262 1.242391406773 0.716539088313
31 H -2.110967622131 -1.331093525107 -0.933439789589
32 H 0.131335808181 -0.791669247340 -1.294811648695
33 H 1.401123778200 3.636853538672 3.147652527768
34 H 3.832549194246 4.138415504546 3.202416865861
35 H 5.331608812660 3.270409417465 1.426540170444
36 H 4.464331890205 1.830167324878 -0.369597136426
37 H 1.298858728113 0.485138748361 -2.435804800896
38 H -2.635298864068 -2.415136868375 0.880111521024
39 H -3.510334392974 -4.709452188207 0.936697906728
40 H -5.948657529395 -5.137141059664 0.687215411243
41 H -7.521539945480 -3.280284765814 0.489849093411
42 C 3.409630159481 0.157550394555 -2.272165884576
43 O 3.436068014386 0.037377430201 -3.502223963144
44 N 4.446857183989 -0.126841735597 -1.446283144543
45 H 4.247949413750 -0.308971221155 -0.465850834208
46 C 5.627458968994 -0.788226148122 -2.017638834734
47 H 5.348718837396 -1.742405424964 -2.476127168677
48 H 6.034851820934 -0.148320398377 -2.801818845143
49 C 6.667663705835 -1.016331453761 -0.936817403302
50 H 6.973600755993 -0.057399386644 -0.486124048269
51 H 6.259022507462 -1.650346078882 -0.129734200530
52 O 7.740181972337 -1.646068165948 -1.585065245654
53 C 8.816311090054 -1.933041577890 -0.695188259970
54 H 8.495184301270 -2.611551406181 0.104273661042
55 H 9.587896267134 -2.413894426194 -1.293477457938
56 H 9.215744701641 -1.013173053099 -0.251782395563

AMOEBA optimized geometry of TO3-Biotin (charge: 2) (.txyz file)

56

1	C	-8.42642347	-1.04459087	0.91645676	403	22	25	26	27
2	C	-6.47728422	0.43046644	1.06645699	409	3	22	28	
3	C	-5.11772846	0.73148477	0.97982117	413	2	4	29	
4	C	-4.16832933	-0.24000306	0.63040848	415	3	5	16	
5	C	-2.77888909	0.25736188	0.44364760	420	4	6	30	
6	C	-1.87984147	-0.16548930	-0.50776888	422	5	7	31	
7	C	-0.50016236	0.21955994	-0.79265872	425	6	8	32	
8	C	0.37724943	1.14236561	-0.20309076	419	7	23	24	
9	C	1.73656669	2.66445944	1.23179265	427	10	14	24	
10	C	2.28842082	3.51329116	2.20549488	423	9	11	33	
11	C	3.62908992	3.90505289	2.08279253	428	10	12	34	
12	C	4.40482965	3.46436626	0.99295286	424	11	13	35	
13	C	3.85902578	2.61646911	0.01171032	426	12	14	36	
14	C	2.50686026	2.19550337	0.14967015	418	9	13	23	
15	C	2.17875007	0.63085836	-1.77369766	410	23	37	42	
16	C	-4.66218162	-1.60378299	0.42693096	411	4	17	21	
17	C	-3.79379730	-2.73380774	0.18248825	416	16	18	38	
18	C	-4.30381491	-4.00815041	-0.03875333	421	17	19	39	
19	C	-5.70156777	-4.22432279	-0.00125248	417	18	20	40	
20	C	-6.57870599	-3.17880377	0.27607855	412	19	21	41	
21	C	-6.08829948	-1.86812627	0.50845269	408	16	20	22	
22	N	-6.95510406	-0.82444785	0.81931731	406	1	2	21	

23	N	1.75096284	1.33967121	-0.71107306	414	8	14	15	
24	S	0.08080504	2.12251306	1.20703580	430	8	9		
25	H	-8.77100509	-1.43074511	-0.04591061	433	1			
26	H	-8.93205919	-0.09986952	1.12820164	433	1			
27	H	-8.60157850	-1.74833825	1.73406873	433	1			
28	H	-7.20396095	1.19447733	1.32341921	437	2			
29	H	-4.82934726	1.76796398	1.17360661	440	3			
30	H	-2.48323248	0.99439127	1.19099909	443	5			
31	H	-2.24413557	-0.90365189	-1.22864535	448	6			
32	H	-0.12862139	-0.34868692	-1.64832798	446	7			
33	H	1.69238901	3.87501845	3.04604018	450	10			
34	H	4.07506083	4.56269890	2.83137108	447	11			
35	H	5.44773054	3.76875764	0.88800997	449	12			
36	H	4.47409611	2.32032910	-0.82869214	445	13			
37	H	1.51932668	0.65574813	-2.64407258	438	15			
38	H	-2.72266487	-2.59508143	0.19004461	441	17			
39	H	-3.62849747	-4.84290774	-0.23501954	444	18			
40	H	-6.09749844	-5.22597032	-0.18222114	442	19			
41	H	-7.63688930	-3.40327392	0.31222443	439	20			
42	C	3.43625864	-0.09400965	-2.18129032	407	15	43	44	
43	O	3.47915148	-0.35483195	-3.38961430	436	42			
44	N	4.44232224	-0.35911255	-1.29236206	405	42	45	46	
45	H	4.32647752	-0.13388180	-0.30764448	435	44			
46	C	5.67454024	-1.02250887	-1.79055632	401	44	47	48	49
47	H	5.41272773	-1.97948870	-2.24885942	432	46			
48	H	6.11997460	-0.41582037	-2.58274832	432	46			
49	C	6.68588937	-1.23368442	-0.64446465	402	46	50	51	52
50	H	6.94453333	-0.26546828	-0.18275941	431	49			
51	H	6.23064538	-1.83980396	0.15723068	431	49			
52	O	7.80570032	-1.86866126	-1.24434361	429	49	53		
53	C	8.87732431	-2.15205939	-0.30876256	404	52	54	55	56
54	H	8.52122751	-2.79347206	0.50011936	434	53			
55	H	9.68122938	-2.66633707	-0.83754968	434	53			
56	H	9.27001434	-1.22440286	0.11241682	434	53			

V. Parameters in Tinker format

TO1-Biotin parameters

atom 419 419 C "TO1-E " 6

12.011 3
atom 423 423 C "TO1-E " 6
12.011 3
atom 415 415 C "TO1-E " 6
12.011 3
atom 401 401 C "TO1-E " 6
12.011 4
atom 404 404 C "TO1-E " 6
12.011 4
atom 403 403 C "TO1-E " 6
12.011 4
atom 408 408 C "TO1-E " 6
12.011 3
atom 411 411 C "TO1-E " 6
12.011 3
atom 414 414 C "TO1-E " 6
12.011 3
atom 410 410 C "TO1-E " 6
12.011 3
atom 405 405 C "TO1-E " 6
12.011 4
atom 409 409 C "TO1-E " 6
12.011 3
atom 413 413 C "TO1-E " 6
12.011 3
atom 417 417 C "TO1-E " 6
12.011 3
atom 424 424 C "TO1-E " 6
12.011 3
atom 416 416 C "TO1-E " 6
12.011 3
atom 412 412 C "TO1-E " 6
12.011 3
atom 425 425 C "TO1-E " 6
12.011 3
atom 420 420 C "TO1-E " 6
12.011 3
atom 427 427 C "TO1-E " 6
12.011 3
atom 421 421 C "TO1-E " 6
12.011 3
atom 426 426 C "TO1-E " 6
12.011 3
atom 418 418 C "TO1-E " 6

12.011 3
atom 406 406 N "TO1-E " 7
14.007 3
atom 422 422 N "TO1-E " 7
14.007 3
atom 407 407 N "TO1-E " 7
14.007 3
atom 429 429 O "TO1-E " 8
15.999 2
atom 437 437 O "TO1-E " 8
15.999 1
atom 430 430 S "TO1-E " 16
32.066 2
atom 442 442 H "TO1-E " 1
1.008 1
atom 432 432 H "TO1-E " 1
1.008 1
atom 433 433 H "TO1-E " 1
1.008 1
atom 434 434 H "TO1-E " 1
1.008 1
atom 440 440 H "TO1-E " 1
1.008 1
atom 438 438 H "TO1-E " 1
1.008 1
atom 435 435 H "TO1-E " 1
1.008 1
atom 439 439 H "TO1-E " 1
1.008 1
atom 445 445 H "TO1-E " 1
1.008 1
atom 446 446 H "TO1-E " 1
1.008 1
atom 444 444 H "TO1-E " 1
1.008 1
atom 448 448 H "TO1-E " 1
1.008 1
atom 447 447 H "TO1-E " 1
1.008 1
atom 449 449 H "TO1-E " 1
1.008 1
atom 441 441 H "TO1-E " 1
1.008 1
atom 436 436 H "TO1-E " 1

1.008 1
atom 402 402 C "TO1-E " 6
12.011 4
atom 431 431 H "TO1-E " 1
1.008 1
atom 428 428 O "TO1-E " 8
15.999 2
atom 450 450 H "TO1-E " 1
1.008 1
atom 443 443 H "TO1-E " 1
1.008 1
vdw 418 3.8200 0.1010
vdw 429 3.4050 0.1100
vdw 428 3.4050 0.1100
vdw 450 2.6550 0.0135 0.910
vdw 432 2.8700 0.0240 0.910
vdw 433 2.8700 0.0240 0.910
vdw 431 2.8700 0.0240 0.910
vdw 401 3.8200 0.1010
vdw 404 3.8200 0.1010
vdw 403 3.8200 0.1010
vdw 405 3.8200 0.1010
vdw 402 3.8200 0.1010
vdw 435 2.9800 0.0240 0.920
vdw 434 2.9600 0.0220 0.920
vdw 423 3.8200 0.1060
vdw 408 3.8200 0.1060
vdw 411 3.8200 0.1060
vdw 437 3.3000 0.1120
vdw 406 3.7100 0.1100
vdw 436 2.5900 0.0220 0.900
vdw 442 2.9200 0.0300 0.920
vdw 443 2.9200 0.0300 0.920
vdw 430 4.0050 0.3550
vdw 419 3.8000 0.0890
vdw 420 3.8000 0.0890
vdw 438 2.9800 0.0260 0.920
vdw 439 2.9800 0.0260 0.920
vdw 444 2.9800 0.0260 0.920
vdw 448 2.9800 0.0260 0.920
vdw 441 2.9800 0.0260 0.920
vdw 414 3.8000 0.0910
vdw 426 3.8000 0.0910
vdw 440 2.9800 0.0260 0.920

vdw 445 2.9800 0.0260 0.920
vdw 446 2.9800 0.0260 0.920
vdw 447 2.9800 0.0260 0.920
vdw 449 2.9800 0.0260 0.920
vdw 407 3.7100 0.1050
vdw 415 3.8000 0.1010
vdw 410 3.8000 0.1010
vdw 409 3.8000 0.1010
vdw 413 3.8000 0.1010
vdw 417 3.8000 0.1010
vdw 424 3.8000 0.1010
vdw 416 3.8000 0.1010
vdw 412 3.8000 0.1010
vdw 425 3.8000 0.1010
vdw 427 3.8000 0.1010
vdw 421 3.8000 0.1010
vdw 422 3.7100 0.1050
bond 419 430 216.0 1.76
bond 411 422 400.0 1.31
bond 407 410 381.30 1.34
bond 408 411 385.0 1.51
bond 430 425 250.0 1.77
bond 437 408 601.80 1.24
bond 442 423 400.0 1.09
bond 443 411 400.0 1.09
bond 415 423 385.0 1.48
bond 423 419 385.0 1.34
bond 422 419 600.0 1.46
bond 422 418 600.0 1.43
bond 409 407 400.0 1.3471999999999997
bond 414 415 379.094345 1.38
bond 412 415 337.112301 1.42
bond 429 401 274.968492 1.43
bond 432 401 340.532535 1.1
bond 402 401 205.758598 1.52
bond 403 404 205.758598 1.51
bond 429 404 274.968492 1.42
bond 433 404 340.532535 1.1
bond 406 403 232.730342 1.47
bond 434 403 340.532535 1.09
bond 406 408 363.15688 1.35
bond 410 414 375.678112 1.39
bond 440 414 368.093365 1.09
bond 438 410 375.586577 1.08

bond 407 405 295.419463 1.4494
bond 435 405 345.97894 1.09
bond 413 409 375.678112 1.41
bond 412 409 375.678112 1.3990999999999998
bond 417 413 379.094345 1.38
bond 439 413 368.093365 1.08
bond 424 417 379.094345 1.41
bond 445 417 368.093365 1.09
bond 416 424 379.094345 1.38
bond 446 424 368.093365 1.09
bond 412 416 337.112301 1.42
bond 444 416 368.093365 1.09
bond 420 425 378.323984 1.39
bond 418 425 375.678112 1.4
bond 427 420 379.094345 1.4
bond 448 420 368.093365 1.09
bond 421 427 379.094345 1.4
bond 447 427 368.093365 1.09
bond 426 421 379.094345 1.39
bond 449 421 368.093365 1.09
bond 418 426 375.678112 1.4
bond 441 426 368.093365 1.08
bond 436 406 475.798234 1.02
bond 431 402 340.532535 1.1
bond 428 402 216.835083 1.42
bond 450 428 497.050438 0.97

angle	419	423	442	50.0	119.9
angle	419	422	411	65.0	119.52
angle	419	430	425	80.0	85.2493
angle	423	419	422	80.0	120.96719999999999
angle	423	419	430	53.2	132.0401
angle	408	411	422	80.0	134.33
angle	411	422	418	65.0	134.19639999999998
angle	418	425	430	53.2	113.39
angle	422	419	430	60.0	107.0648
angle	422	411	443	60.0	115.08
angle	403	404	429	88.0	104.62
angle	402	401	429	88.0	112.949
angle	430	425	420	60.0	126.68
angle	438	410	407	58.99	116.8
angle	443	411	408	38.0	110.4
angle	405	407	410	51.80	118.48
angle	437	408	411	65.0	114.07550000000002
angle	406	408	411	60.0	119.83359999999999

angle 406 408 437 76.98 126.23
angle 415 423 442 50.0 119.39959999999999
angle 409 407 405 65.0 118.53649999999999
angle 415 423 419 60.0 120.36589999999998
angle 407 410 414 60.0 121.38
angle 422 418 425 60.0 108.79489999999998
angle 422 418 426 60.0 127.04
angle 409 407 410 65.0 121.66
angle 412 415 423 60.0 122.08
angle 413 409 407 80.0 118.9745
angle 412 409 407 80.0 118.67
angle 414 415 423 60.0 118.81
angle 418 422 419 65.0 105.6393
angle 410 414 415 89.183524 120.2
angle 440 414 415 36.770174 121.32
angle 409 412 415 89.183524 119.02
angle 416 412 415 78.148553 122.67
angle 404 429 401 88.186905 112.08529999999999
angle 431 402 401 48.260518 110.71
angle 428 402 401 94.530114 103.89
angle 406 403 404 99.687701 104.74660000000002
angle 434 403 404 48.260518 111.22
angle 433 404 403 48.260518 110.26
angle 408 406 403 87.947117 116.052
angle 436 406 403 29.797294 111.62
angle 436 406 408 40.855201 114.67800000000001
angle 412 415 414 59.084556 119.0
angle 438 410 414 59.926322 123.49129999999998
angle 440 414 410 47.265893 118.47
angle 417 413 409 89.183524 119.36
angle 439 413 409 47.265893 119.64210000000001
angle 416 412 409 89.183524 118.3
angle 412 409 413 59.084556 121.74650000000001
angle 424 417 413 74.630713 120.9
angle 445 417 413 36.770174 119.35
angle 439 413 417 36.770174 121.7983
angle 416 424 417 74.630713 120.59
angle 446 424 417 36.770174 119.52
angle 445 417 424 36.770174 119.73
angle 412 416 424 59.084556 120.44
angle 444 416 424 36.770174 122.93060000000001
angle 446 424 416 36.770174 119.89
angle 444 416 412 26.510066 116.906
angle 427 420 425 68.376488 118.24

angle 448 420 425 29.804975 121.03
angle 426 418 425 76.194709 122.2
angle 418 425 420 89.183524 119.9
angle 421 427 420 74.630713 121.21
angle 447 427 420 36.770174 119.18
angle 448 420 427 36.770174 120.69
angle 426 421 427 74.630713 120.94
angle 449 421 427 36.770174 119.78
angle 447 427 421 36.770174 119.57
angle 418 426 421 89.183524 117.09
angle 441 426 421 36.770174 122.97119999999998
angle 449 421 426 36.770174 119.25
angle 441 426 418 47.265893 121.66
angle 434 403 406 57.622801 109.71
angle 435 405 407 53.335255 106.86390000000002
angle 432 401 429 54.887155 107.82
angle 433 404 429 54.887155 111.69
angle 432 401 432 30.490529 110.2527
angle 402 401 432 48.260518 108.71
angle 433 404 433 30.490529 108.3
angle 434 403 434 30.490529 110.709
angle 435 405 435 36.406852 112.96910000000001
angle 450 428 402 48.760726 106.30720000000001
angle 431 402 431 30.490529 108.27
angle 428 402 431 60.032562 111.64

strbnd	419	423	442	0	0
strbnd	419	422	411	0	0
strbnd	419	430	425	0	0
strbnd	423	419	422	0	0
strbnd	423	419	430	0	0
strbnd	408	411	422	0	0
strbnd	411	422	418	0	0
strbnd	418	425	430	0	0
strbnd	422	419	430	0	0
strbnd	422	411	443	0	0
strbnd	403	404	429	0	0
strbnd	402	401	429	0	0
strbnd	430	425	420	0	0
strbnd	438	410	407	11.50	11.50
strbnd	443	411	408	0	0
strbnd	405	407	410	7.20	7.20
strbnd	437	408	411	0	0
strbnd	406	408	411	0	0
strbnd	406	408	437	18.70	18.70

strbnd 415 423 442 0 0
strbnd 409 407 405 0 0
strbnd 415 423 419 0 0
strbnd 407 410 414 0 0
strbnd 422 418 425 0 0
strbnd 422 418 426 0 0
strbnd 409 407 410 0 0
strbnd 412 415 423 0 0
strbnd 413 409 407 0 0
strbnd 412 409 407 0 0
strbnd 414 415 423 0 0
strbnd 410 414 415 20.4528 20.4528
strbnd 440 414 415 20.4528 20.4528
strbnd 409 412 415 20.4528 20.4528
strbnd 416 412 415 20.4528 20.4528
strbnd 404 429 401 6.8713 6.8713
strbnd 431 402 401 5.7126 5.7126
strbnd 428 402 401 5.7126 5.7126
strbnd 406 403 404 5.7126 5.7126
strbnd 434 403 404 5.7126 5.7126
strbnd 433 404 403 5.7126 5.7126
strbnd 408 406 403 25.9516 25.9516
strbnd 436 406 403 25.9516 25.9516
strbnd 436 406 408 25.9516 25.9516
strbnd 412 415 414 20.4528 20.4528
strbnd 438 410 414 36.8955 36.8955
strbnd 440 414 410 20.4528 20.4528
strbnd 417 413 409 20.4528 20.4528
strbnd 439 413 409 20.4528 20.4528
strbnd 416 412 409 20.4528 20.4528
strbnd 412 409 413 20.4528 20.4528
strbnd 424 417 413 20.4528 20.4528
strbnd 445 417 413 20.4528 20.4528
strbnd 439 413 417 20.4528 20.4528
strbnd 416 424 417 20.4528 20.4528
strbnd 446 424 417 20.4528 20.4528
strbnd 445 417 424 20.4528 20.4528
strbnd 412 416 424 20.4528 20.4528
strbnd 444 416 424 20.4528 20.4528
strbnd 446 424 416 20.4528 20.4528
strbnd 444 416 412 20.4528 20.4528
strbnd 427 420 425 20.4528 20.4528
strbnd 448 420 425 20.4528 20.4528
strbnd 426 418 425 20.4528 20.4528

strbnd 418 425 420 20.4528 20.4528
strbnd 421 427 420 20.4528 20.4528
strbnd 447 427 420 20.4528 20.4528
strbnd 448 420 427 20.4528 20.4528
strbnd 426 421 427 20.4528 20.4528
strbnd 449 421 427 20.4528 20.4528
strbnd 447 427 421 20.4528 20.4528
strbnd 418 426 421 20.4528 20.4528
strbnd 441 426 421 20.4528 20.4528
strbnd 449 421 426 20.4528 20.4528
strbnd 441 426 418 20.4528 20.4528
strbnd 434 403 406 5.7126 5.7126
strbnd 435 405 407 5.7126 5.7126
strbnd 432 401 429 5.7126 5.7126
strbnd 433 404 429 5.7126 5.7126
strbnd 432 401 432 5.7126 5.7126
strbnd 402 401 432 5.7126 5.7126
strbnd 433 404 433 5.7126 5.7126
strbnd 434 403 434 5.7126 5.7126
strbnd 435 405 435 5.7126 5.7126
strbnd 450 428 402 6.2784 6.2784
strbnd 431 402 431 5.7126 5.7126
strbnd 428 402 431 5.7126 5.7126
opbend 411 408 0 0 42.40
opbend 442 423 0 0 140.30
opbend 443 411 0 0 140.30
opbend 415 423 0 0 42.40
opbend 405 407 0 0 12.90
opbend 419 423 0 0 14.40
opbend 422 419 0 0 18.00
opbend 422 418 0 0 18.00
opbend 423 415 0 0 14.40
opbend 407 409 0 0 14.40
opbend 419 422 0 0 10.80
opbend 418 422 0 0 10.80
opbend 414 415 0 0 84.8861
opbend 415 414 0 0 84.8861
opbend 412 415 0 0 100.1116
opbend 415 412 0 0 100.1116
opbend 403 406 0 0 16.5457
opbend 406 408 0 0 92.7841
opbend 408 406 0 0 92.7841
opbend 437 408 0 0 16.5045
opbend 410 414 0 0 34.0822

opbend 414 410 0 0 34.0822
opbend 440 414 0 0 72.8135
opbend 438 410 0 0 72.8135
opbend 413 409 0 0 47.0937
opbend 409 413 0 0 47.0937
opbend 412 409 0 0 47.0937
opbend 409 412 0 0 47.0937
opbend 417 413 0 0 84.8861
opbend 413 417 0 0 84.8861
opbend 439 413 0 0 72.8135
opbend 424 417 0 0 84.8861
opbend 417 424 0 0 84.8861
opbend 445 417 0 0 72.8135
opbend 416 424 0 0 84.8861
opbend 424 416 0 0 84.8861
opbend 446 424 0 0 72.8135
opbend 412 416 0 0 100.1116
opbend 416 412 0 0 100.1116
opbend 444 416 0 0 72.8135
opbend 420 425 0 0 14.9839
opbend 425 420 0 0 14.9839
opbend 418 425 0 0 47.0937
opbend 425 418 0 0 47.0937
opbend 427 420 0 0 84.8861
opbend 420 427 0 0 84.8861
opbend 448 420 0 0 72.8135
opbend 421 427 0 0 84.8861
opbend 427 421 0 0 84.8861
opbend 447 427 0 0 72.8135
opbend 426 421 0 0 84.8861
opbend 421 426 0 0 84.8861
opbend 449 421 0 0 72.8135
opbend 418 426 0 0 39.6142
opbend 426 418 0 0 39.6142
opbend 441 426 0 0 72.8135
opbend 436 406 0 0 26.8945
opbend 423 419 0 0 14.39
opbend 430 419 0 0 14.39
opbend 422 411 0 0 14.39
opbend 411 422 0 0 3.6
opbend 408 411 0 0 14.39
opbend 407 410 0 0 14.39
opbend 410 407 0 0 3.6
opbend 409 407 0 0 3.6

opbend 430 425 0 0 14.39
torsion 414 415 423 419 -0.480 0.0 1 14.422 180.0 2 4.720 0.0 3
torsion 412 415 423 419 -2.723 0.0 1 -16.439 180.0 2 3.870 0.0 3
torsion 408 411 422 419 0.000 0.0 1 0.000 180.0 2 0.299 0.0 3
torsion 443 411 422 419 0.000 0.0 1 0.000 180.0 2 0.299 0.0 3
torsion 420 425 430 419 0.854 0.0 1 -0.374 180.0 2 0.108 0.0 3
torsion 418 425 430 419 0.854 0.0 1 -0.374 180.0 2 0.108 0.0 3
torsion 411 422 419 423 -0.67 0.0 1 6.287 180.0 2 0 0.0 3
torsion 418 422 419 423 -0.67 0.0 1 6.287 180.0 2 0 0.0 3
torsion 425 430 419 423 0.854 0.0 1 -0.374 180.0 2 0.108 0.0 3
torsion 422 419 423 415 0.000 0.0 1 0.000 180.0 2 0.299 0.0 3
torsion 430 419 423 415 0.000 0.0 1 0.000 180.0 2 0.299 0.0 3
torsion 418 422 411 408 0.000 0.0 1 0.000 180.0 2 0.299 0.0 3
torsion 430 419 422 411 -0.67 0.0 1 6.287 180.0 2 0 0.0 3
torsion 425 418 422 411 -0.67 0.0 1 6.287 180.0 2 0 0.0 3
torsion 426 418 422 411 -0.67 0.0 1 6.287 180.0 2 0 0.0 3
torsion 412 409 407 405 -0.67 0.0 1 6.287 180.0 2 0 0.0 3
torsion 422 419 430 425 0.854 0.0 1 -0.374 180.0 2 0.108 0.0 3
torsion 430 425 418 426 -0.67 0.0 1 6.287 180.0 2 0 0.0 3
torsion 430 419 422 418 -0.67 0.0 1 6.287 180.0 2 0 0.0 3
torsion 443 411 422 418 0.000 0.0 1 0.000 180.0 2 0.299 0.0 3
torsion 406 403 404 429 -0.628 0.0 1 -0.391 180.0 2 0.534 0.0 3
torsion 406 408 411 422 0.924 0.0 1 -0.102 180.0 2 0.514 0.0 3
torsion 442 423 419 422 0.000 0.0 1 0.000 180.0 2 0.299 0.0 3
torsion 437 408 411 422 0.063 0.0 1 -0.102 180.0 2 -0.807 0.0 3
torsion 430 425 418 422 -0.67 0.0 1 6.287 180.0 2 0 0.0 3
torsion 442 423 419 430 0.000 0.0 1 0.000 180.0 2 0.299 0.0 3
torsion 430 425 420 427 -0.67 0.0 1 6.287 180.0 2 0 0.0 3
torsion 430 425 420 448 0.55 0.0 1 6.187 180.0 2 -0.55 0.0 3
torsion 405 407 410 414 -0.67 0.0 1 6.287 180.0 2 0 0.0 3
torsion 435 405 407 410 0.072 0.0 1 -0.012 180.0 2 0.563 0.0 3
torsion 438 410 407 405 0.121 0.0 1 -0.648 180.0 2 0.199 0.0 3
torsion 437 408 411 443 0 0.0 1 0 180.0 2 0.108 0.0 3
torsion 406 408 411 443 0 0.0 1 0 180.0 2 0.108 0.0 3
torsion 435 405 407 409 0 0.0 1 0 180.0 2 0.108 0.0 3
torsion 403 406 408 411 -1.000 0.0 1 4.283333333333333 180.0 2 2.050 0.0 3
torsion 405 407 409 413 -0.67 0.0 1 6.287 180.0 2 0 0.0 3
torsion 436 406 408 411 0.000 0.0 1 3.4833333333333334 180.0 2 0.800 0.0 3
torsion 437 408 406 403 1.000 0.0 1 9.1 180.0 2 -2.250 0.0 3
torsion 436 406 408 437 0.000 0.0 1 1.619333333333333 180.0 2 -0.357 0.0 3
torsion 412 415 423 442 0 0.0 1 0 180.0 2 0.108 0.0 3
torsion 407 410 414 415 -0.67 0.0 1 6.287 180.0 2 0 0.0 3
torsion 422 418 425 420 -0.67 0.0 1 6.287 180.0 2 0 0.0 3
torsion 422 418 426 421 -0.67 0.0 1 6.287 180.0 2 0 0.0 3

torsion 438 410 414 440 0.000 0.0 1 6.355333333333333 180.0 2 0.000 0.0 3
torsion 422 418 426 441 0.55 0.0 1 6.187 180.0 2 -0.55 0.0 3
torsion 407 410 414 440 0.55 0.0 1 6.187 180.0 2 -0.55 0.0 3
torsion 419 422 418 425 -0.67 0.0 1 6.287 180.0 2 0 0.0 3
torsion 419 422 418 426 -0.67 0.0 1 6.287 180.0 2 0 0.0 3
torsion 421 426 418 425 -0.67 0.0 1 6.287 180.0 2 0 0.0 3
torsion 418 425 420 427 -0.67 0.0 1 6.287 180.0 2 0 0.0 3
torsion 418 426 421 427 -0.67 0.0 1 6.287 180.0 2 0 0.0 3
torsion 447 427 420 425 0.55 0.0 1 6.187 180.0 2 -0.55 0.0 3
torsion 449 421 426 418 0.55 0.0 1 6.187 180.0 2 -0.55 0.0 3
torsion 449 421 427 447 0.000 0.0 1 10.922 180.0 2 0.000 0.0 3
torsion 409 407 410 438 0.55 0.0 1 6.187 180.0 2 -0.55 0.0 3
torsion 414 410 407 409 -0.67 0.0 1 6.287 180.0 2 0 0.0 3
torsion 413 409 407 410 -0.67 0.0 1 6.287 180.0 2 0 0.0 3
torsion 412 409 407 410 -0.67 0.0 1 6.287 180.0 2 0 0.0 3
torsion 409 412 415 423 -0.67 0.0 1 6.287 180.0 2 0 0.0 3
torsion 416 412 415 423 -0.67 0.0 1 6.287 180.0 2 0 0.0 3
torsion 445 417 413 439 0.000 0.0 1 7.072 180.0 2 0.000 0.0 3
torsion 446 424 417 445 0.000 0.0 1 7.072 180.0 2 0.000 0.0 3
torsion 444 416 424 446 0.000 0.0 1 7.072 180.0 2 0.000 0.0 3
torsion 447 427 420 448 0.000 0.0 1 7.072 180.0 2 0.000 0.0 3
torsion 441 426 421 449 0.000 0.0 1 7.072 180.0 2 0.000 0.0 3
torsion 407 409 413 439 0.55 0.0 1 6.187 180.0 2 -0.55 0.0 3
torsion 415 412 416 444 0.250 0.0 1 5.534 180.0 2 -0.550 0.0 3
torsion 409 413 417 445 0.250 0.0 1 5.534 180.0 2 -0.550 0.0 3
torsion 409 412 416 444 0.250 0.0 1 5.534 180.0 2 -0.550 0.0 3
torsion 413 417 424 446 0.250 0.0 1 5.534 180.0 2 -0.550 0.0 3
torsion 417 424 416 444 0.250 0.0 1 5.534 180.0 2 -0.550 0.0 3
torsion 424 417 413 439 0.250 0.0 1 5.534 180.0 2 -0.550 0.0 3
torsion 416 424 417 445 0.250 0.0 1 5.534 180.0 2 -0.550 0.0 3
torsion 412 409 413 439 0.250 0.0 1 5.534 180.0 2 -0.550 0.0 3
torsion 412 416 424 446 0.250 0.0 1 5.534 180.0 2 -0.550 0.0 3
torsion 425 418 426 441 0.55 0.0 1 6.187 180.0 2 -0.55 0.0 3
torsion 420 427 421 449 0.250 0.0 1 5.534 180.0 2 -0.550 0.0 3
torsion 427 421 426 441 0.250 0.0 1 5.534 180.0 2 -0.550 0.0 3
torsion 421 427 420 448 0.250 0.0 1 5.534 180.0 2 -0.550 0.0 3
torsion 426 421 427 447 0.250 0.0 1 5.534 180.0 2 -0.550 0.0 3
torsion 418 425 420 448 0.55 0.0 1 6.187 180.0 2 -0.55 0.0 3
torsion 415 412 409 407 -0.67 0.0 1 6.287 180.0 2 0 0.0 3
torsion 417 413 409 407 -0.67 0.0 1 6.287 180.0 2 0 0.0 3
torsion 416 412 409 407 -0.67 0.0 1 6.287 180.0 2 0 0.0 3
torsion 413 409 412 415 -0.670 0.0 1 4.304 180.0 2 0.000 0.0 3
torsion 424 416 412 415 -0.670 0.0 1 4.304 180.0 2 0.000 0.0 3
torsion 424 417 413 409 -0.670 0.0 1 4.304 180.0 2 0.000 0.0 3

torsion 424 416 412 409 -0.670 0.0 1 4.304 180.0 2 0.000 0.0 3
torsion 416 412 409 413 -0.670 0.0 1 4.304 180.0 2 0.000 0.0 3
torsion 416 424 417 413 -0.670 0.0 1 4.304 180.0 2 0.000 0.0 3
torsion 412 409 413 417 -0.670 0.0 1 4.304 180.0 2 0.000 0.0 3
torsion 412 416 424 417 -0.670 0.0 1 4.304 180.0 2 0.000 0.0 3
torsion 421 427 420 425 -0.67 0.0 1 6.287 180.0 2 0 0.0 3
torsion 426 418 425 420 -0.67 0.0 1 6.287 180.0 2 0 0.0 3
torsion 426 421 427 420 -0.670 0.0 1 4.304 180.0 2 0.000 0.0 3
torsion 414 415 412 409 -0.610 0.0 1 4.212 180.0 2 0.000 0.0 3
torsion 414 415 412 416 -0.610 0.0 1 4.212 180.0 2 0.000 0.0 3
torsion 423 415 414 440 0.55 0.0 1 6.187 180.0 2 -0.55 0.0 3
torsion 412 415 414 440 0.000 0.0 1 0.000 180.0 2 0.341 0.0 3
torsion 410 414 415 423 -0.67 0.0 1 6.287 180.0 2 0 0.0 3
torsion 410 414 415 412 0.260 0.0 1 -0.255 180.0 2 0.260 0.0 3
torsion 415 414 410 438 0.000 0.0 1 0.000 180.0 2 0.341 0.0 3
torsion 436 406 403 434 1.397 0 1 0.709 180 2 -0.436 0 3
torsion 450 428 402 431 -0.448 0 1 0.493 180 2 -0.096 0 3
torsion 434 403 404 429 0.0 0 1 0.0 180 2 0.108 0 3
torsion 434 403 404 433 0.0 0 1 0.0 180 2 0.299 0 3
torsion 434 403 406 408 0.0 0 1 0.0 180 2 0.108 0 3
torsion 431 402 401 429 0.0 0 1 0.0 180 2 0.108 0 3
torsion 431 402 401 432 0.0 0 1 0.0 180 2 0.299 0 3
torsion 406 403 404 433 0.195 0.0 1 -0.098 180.0 2 0.176 0.0 3
torsion 436 406 403 404 0.0 0 1 0.0 180 2 0.108 0 3
torsion 408 406 403 404 -0.879 0 1 -0.305 180 2 -1.398 0 3
torsion 414 415 423 442 0 0.0 1 0 180.0 2 0.108 0.0 3
torsion 433 404 429 401 0.0 0 1 0.0 180 2 0.108 0 3
torsion 403 404 429 401 -0.508 0 1 0.088 180 2 1.122 0 3
torsion 402 401 429 404 1.298 0 1 -0.514 180 2 1.129 0 3
torsion 432 401 429 404 0.0 0 1 0.0 180 2 0.108 0 3
torsion 428 402 401 429 0.912 0 1 -1.127 180 2 2.601 0 3
torsion 428 402 401 432 0.0 0 1 0.0 180 2 0.108 0 3
torsion 450 428 402 401 -0.416 0 1 0.144 180 2 1.086 0 3
SOLUTE 450 2.6533 3.392 2.9044
SOLUTE 442 2.574 2.758 2.9054
SOLUTE 440 2.574 2.758 2.9054
SOLUTE 438 2.9059 2.602 2.5726
SOLUTE 439 2.574 2.758 2.9054
SOLUTE 445 2.574 2.758 2.9054
SOLUTE 446 2.574 2.758 2.9054
SOLUTE 444 2.574 2.758 2.9054
SOLUTE 448 2.574 2.758 2.9054
SOLUTE 447 2.574 2.758 2.9054
SOLUTE 449 2.574 2.758 2.9054

SOLUTE 441 2.574 2.758 2.9054
SOLUTE 443 2.574 2.758 2.9054
SOLUTE 434 3.0556 2.996 4.032
SOLUTE 436 3.6491 3.404 2.9894
SOLUTE 431 3.6491 3.404 2.9894
SOLUTE 432 3.0205 3.885 4.0505
SOLUTE 433 3.9912 4.144 3.8925
SOLUTE 435 3.143 3.374 4.144
SOLUTE 410 3.8286 3.893 4.5084
SOLUTE 419 3.8286 3.893 4.5084
SOLUTE 409 3.8286 3.893 4.5084
SOLUTE 418 3.8585 3.766 3.8448
SOLUTE 415 3.8286 3.893 4.5084
SOLUTE 414 3.8286 3.893 4.5084
SOLUTE 413 3.8286 3.893 4.5084
SOLUTE 416 3.8286 3.893 4.5084
SOLUTE 425 3.8585 3.766 3.8448
SOLUTE 420 3.8286 3.893 4.5084
SOLUTE 426 3.8286 3.893 4.5084
SOLUTE 408 2.9301 4.506 4.2118
SOLUTE 417 3.8286 3.893 4.5084
SOLUTE 424 3.8286 3.893 4.5084
SOLUTE 412 3.8286 3.893 4.5084
SOLUTE 427 3.8286 3.893 4.5084
SOLUTE 421 3.8286 3.893 4.5084
SOLUTE 423 2.9301 4.506 4.2118
SOLUTE 411 2.9301 4.506 4.2118
SOLUTE 405 3.5062 3.309 4.536
SOLUTE 401 3.3025 2.854 4.9016
SOLUTE 404 3.3025 2.854 4.9016
SOLUTE 403 3.3025 2.854 4.9016
SOLUTE 402 3.3025 2.854 4.9016
SOLUTE 422 3.4243 3.491 4.2676
SOLUTE 407 3.4243 3.491 4.2676
SOLUTE 406 2.8836 2.99 3.218
SOLUTE 437 2.9835 3.356 2.9616
SOLUTE 429 3.1684 3.134 3.0999
SOLUTE 428 3.1684 3.134 3.0999
SOLUTE 430 4.4346 4.194 4.5431
polarize 419 2.0645 0.3900 422 430
polarize 423 1.4150 0.3900 442
polarize 415 2.0645 0.3900 414 412
polarize 401 1.6196 0.3900 429 432
polarize 404 1.6196 0.3900 429 433

polarize	403	1.6196	0.3900 434
polarize	408	2.0645	0.3900 406 437
polarize	411	1.4150	0.3900 443
polarize	414	2.0645	0.3900 415 410 440
polarize	410	2.0645	0.3900 414 407 438
polarize	405	1.6196	0.3900 435
polarize	409	2.0645	0.3900 413 412 407
polarize	413	2.0645	0.3900 409 417 439
polarize	417	2.0645	0.3900 413 424 445
polarize	424	2.0645	0.3900 417 416 446
polarize	416	2.0645	0.3900 424 412 444
polarize	412	2.0645	0.3900 415 409 416
polarize	425	2.0645	0.3900 420 418 430
polarize	420	2.0645	0.3900 425 427 448
polarize	427	2.0645	0.3900 420 421 447
polarize	421	2.0645	0.3900 427 426 449
polarize	426	2.0645	0.3900 421 418 441
polarize	418	2.0645	0.3900 425 426 422
polarize	406	1.2433	0.3900 408 436
polarize	422	1.7018	0.3900 419 418
polarize	407	1.7018	0.3900 410 409
polarize	429	0.8122	0.3900 401 404
polarize	437	0.9138	0.3900 408
polarize	430	3.2059	0.3900 419 425
polarize	442	0.4803	0.3900 423
polarize	432	0.4803	0.3900 401
polarize	433	0.4803	0.3900 404
polarize	434	0.4803	0.3900 403
polarize	440	0.4318	0.3900 414
polarize	438	0.4318	0.3900 410
polarize	435	0.4803	0.3900 405
polarize	439	0.4318	0.3900 413
polarize	445	0.4318	0.3900 417
polarize	446	0.4318	0.3900 424
polarize	444	0.4318	0.3900 416
polarize	448	0.4318	0.3900 420
polarize	447	0.4318	0.3900 427
polarize	449	0.4318	0.3900 421
polarize	441	0.4318	0.3900 426
polarize	436	0.4573	0.3900 406
polarize	402	1.6196	0.3900 431 428
polarize	431	0.4803	0.3900 402
polarize	428	0.8122	0.3900 402 450
polarize	450	0.4573	0.3900 428

polarize 443 0.4803 0.3900 411
multipole 419 430 422 -0.22823
0.18994 0.00000 -0.26779
0.43468
0.00000 -0.14760
-0.38505 0.00000 -0.28708
multipole 423 442 415 -0.14327
-0.03283 0.00000 -0.24378
-0.21202
0.00000 0.06064
0.23935 0.00000 0.15138
multipole 415 412 414 -0.07777
-0.28161 0.00000 -0.04373
-0.08134
0.00000 0.51510
-0.23789 0.00000 -0.43376
multipole 401 402 429 0.07114
0.42578 0.00000 -0.00026
0.39558
0.00000 -0.56522
-0.36574 0.00000 0.16964
multipole 404 429 403 0.06569
0.25578 0.00000 0.33266
0.00125
0.00000 -0.51032
-0.05230 0.00000 0.50907
multipole 403 406 404 -0.04002
0.20620 0.00000 0.07646
0.12238
0.00000 -0.54336
-0.20156 0.00000 0.42098
multipole 408 437 406 0.71246
-0.07646 0.00000 0.26776
0.07472
0.00000 -0.18634
0.09823 0.00000 0.11162
multipole 411 443 422 0.04500
0.14958 0.00000 -0.10449
0.16370
0.00000 0.25451
-0.32747 0.00000 -0.41821
multipole 414 440 410 -0.10584
-0.11618 0.00000 0.32915
0.04056

```
                                 0.00000   0.15954
                                -0.05380   0.00000  -0.20010
multipole   410  438  407        0.26395
                                 0.25601   0.00000  -0.19513
                                -0.11635
                                 0.00000  -0.52155
                                -0.20792   0.00000   0.63790
multipole   405  407  409       -0.07500
                                -0.06928   0.00000   0.49814
                                -0.46189
                                 0.00000  -0.50082
                                -0.07817   0.00000   0.96271
multipole   409  407  412        0.08054
                                -0.09848   0.00000   0.06035
                                -0.85295
                                 0.00000  -0.08638
                                -0.00035   0.00000   0.93933
multipole   413  439  417       -0.10830
                                -0.16453   0.00000   0.23837
                                 0.13298
                                 0.00000  -0.01324
                                 0.14099   0.00000  -0.11974
multipole   417  445  424        0.30169
                                 0.01313   0.00000  -0.40834
                                -0.80929
                                 0.00000  -0.76050
                                -0.06625   0.00000   1.56979
multipole   424  446  416       -0.00401
                                 0.00630   0.00000   0.17381
                                 0.21423
                                 0.00000   0.00438
                                -0.07560   0.00000  -0.21861
multipole   416  444  412       -0.19528
                                -0.17316   0.00000   0.26512
                                -0.11654
                                 0.00000   0.11648
                                 0.19812   0.00000   0.00006
multipole   412  416  409        0.01011
                                -0.07833   0.00000   0.07784
                                -0.15988
                                 0.00000   0.70605
                                -0.25498   0.00000  -0.54617
multipole   425  430  418       -0.25554
                                -0.01764   0.00000   0.04851
```

				0.20411		
				0.00000	0.10046	
				-0.19759	0.00000	-0.30457
multipole	420	448	427	-0.07002		
				0.05627	0.00000	0.20446
				0.04495		
				0.00000	0.05750	
				-0.20524	0.00000	-0.10245
multipole	427	447	421	0.04608		
				0.03159	0.00000	-0.00587
				-0.03645		
				0.00000	-0.03725	
				-0.03520	0.00000	0.07370
multipole	421	449	426	-0.02429		
				-0.03498	0.00000	0.10175
				0.16116		
				0.00000	-0.02840	
				-0.02213	0.00000	-0.13276
multipole	426	441	418	-0.11301		
				0.12946	0.00000	0.17794
				0.11695		
				0.00000	0.11344	
				0.05927	0.00000	-0.23039
multipole	418	422	426	0.22996		
				-0.18300	0.00000	-0.51427
				-1.16989		
				0.00000	-1.09858	
				0.47895	0.00000	2.26847
multipole	406	436	408	-0.20500		
				-0.13587	0.00000	0.18086
				0.17332		
				0.00000	-0.54722	
				0.13140	0.00000	0.37390
multipole	422	418	411	0.27933		
				0.08389	0.00000	0.10942
				0.38374		
				0.00000	-0.44294	
				0.03087	0.00000	0.05920
multipole	407	409	405	0.18492		
				-0.23109	0.00000	0.05299
				1.89213		
				0.00000	-1.24307	
				-1.25595	0.00000	-0.64906
multipole	429	404	401	-0.33032		

				0.34821	0.00000	0.17891
				0.16531		
				0.00000	-1.00658	
				-0.55006	0.00000	0.84127
multipole	437	408	406	-0.60533		
				0.03837	0.00000	-0.07788
				-0.45443		
				0.00000	0.20499	
				0.04325	0.00000	0.24944
multipole	430	425	419	0.53116		
				0.59624	0.00000	0.51435
				1.12713		
				0.00000	-2.15635	
				0.01138	0.00000	1.02922
multipole	442	423	415	0.12022		
				0.05061	0.00000	-0.19068
				0.13375		
				0.00000	0.06535	
				0.09409	0.00000	-0.19910
multipole	432	401	402	0.04779		
				0.04084	0.00000	-0.08574
				0.03838		
				0.00000	0.01541	
				0.09149	0.00000	-0.05379
multipole	433	404	433	0.04192		
				0.00505	0.00000	-0.10510
				0.05296		
				0.00000	0.00101	
				0.02418	0.00000	-0.05397
multipole	434	403	434	0.09207		
				-0.04721	0.00000	-0.12965
				0.09422		
				0.00000	0.03951	
				-0.02511	0.00000	-0.13373
multipole	440	414	410	0.09150		
				-0.01030	0.00000	-0.11411
				0.07275		
				0.00000	-0.04835	
				0.01069	0.00000	-0.02440
multipole	438	410	407	0.08191		
				-0.01544	0.00000	-0.04568
				0.01857		
				0.00000	-0.06506	
				-0.02963	0.00000	0.04649

multipole	435	405	407	0.10504		
				0.03232	0.00000	-0.00173
				0.03155		
				0.00000	-0.05626	
				0.02998	0.00000	0.02471
multipole	439	413	417	0.07680		
				-0.05159	0.00000	-0.17562
				0.22322		
				0.00000	-0.08238	
				0.04841	0.00000	-0.14084
multipole	445	417	424	0.05556		
				0.00732	0.00000	0.04164
				-0.05535		
				0.00000	-0.10123	
				0.00805	0.00000	0.15658
multipole	446	424	416	0.05501		
				-0.00503	0.00000	-0.11867
				0.01053		
				0.00000	-0.01205	
				-0.01469	0.00000	0.00152
multipole	444	416	412	0.10631		
				0.15131	0.00000	-0.15273
				0.36776		
				0.00000	-0.18823	
				0.05386	0.00000	-0.17953
multipole	448	420	427	0.04689		
				0.01769	0.00000	-0.14689
				0.07708		
				0.00000	-0.02384	
				-0.00011	0.00000	-0.05324
multipole	447	427	421	0.05597		
				-0.01212	0.00000	-0.11751
				0.05098		
				0.00000	-0.02922	
				-0.01360	0.00000	-0.02176
multipole	449	421	426	0.05801		
				0.02672	0.00000	-0.14513
				0.07342		
				0.00000	0.00083	
				0.01250	0.00000	-0.07425
multipole	441	426	418	0.05912		
				-0.03292	0.00000	-0.19646
				0.15752		
				0.00000	0.04886	

-0.00751 0.00000 -0.20638
multipole 436 406 408 0.15183
-0.12467 0.00000 -0.12334
-0.03581
0.00000 0.10862
-0.07126 0.00000 -0.07281
multipole 402 428 401 0.11936
0.19945 0.00000 0.16810
-0.01740
0.00000 -0.34885
-0.29032 0.00000 0.36625
multipole 431 402 428 0.03463
0.01460 0.00000 -0.04069
0.02305
0.00000 -0.02844
-0.00863 0.00000 0.00539
multipole 428 450 402 -0.39391
0.39572 0.00000 -0.08327
0.56124
0.00000 -0.66438
-0.43514 0.00000 0.10314
multipole 450 428 402 0.23855
-0.07521 0.00000 -0.14168
-0.00949
0.00000 0.00186
-0.06292 0.00000 0.00763
multipole 443 411 422 0.08813
0.07024 0.00000 -0.11076
0.11547
0.00000 0.00615
0.05162 0.00000 -0.12162

TO3-Biotin parameters

atom 403 403 C "TO3-E " 6
12.011 4
atom 409 409 C "TO3-E " 6
12.011 3
atom 413 413 C "TO3-E " 6
12.011 3
atom 415 415 C "TO3-E " 6
12.011 3
atom 420 420 C "TO3-E " 6
12.011 3
atom 422 422 C "TO3-E " 6
12.011 3

atom 425 425 C "TO3-E " 6
12.011 3
atom 419 419 C "TO3-E " 6
12.011 3
atom 427 427 C "TO3-E " 6
12.011 3
atom 423 423 C "TO3-E " 6
12.011 3
atom 428 428 C "TO3-E " 6
12.011 3
atom 424 424 C "TO3-E " 6
12.011 3
atom 426 426 C "TO3-E " 6
12.011 3
atom 418 418 C "TO3-E " 6
12.011 3
atom 410 410 C "TO3-E " 6
12.011 3
atom 411 411 C "TO3-E " 6
12.011 3
atom 416 416 C "TO3-E " 6
12.011 3
atom 421 421 C "TO3-E " 6
12.011 3
atom 417 417 C "TO3-E " 6
12.011 3
atom 412 412 C "TO3-E " 6
12.011 3
atom 408 408 C "TO3-E " 6
12.011 3
atom 406 406 N "TO3-E " 7
14.007 3
atom 414 414 N "TO3-E " 7
14.007 3
atom 430 430 S "TO3-E " 16
32.066 2
atom 433 433 H "TO3-E " 1
1.008 1
atom 437 437 H "TO3-E " 1
1.008 1
atom 440 440 H "TO3-E " 1
1.008 1
atom 443 443 H "TO3-E " 1
1.008 1

atom 448 448 H "TO3-E " 1
1.008 1
atom 446 446 H "TO3-E " 1
1.008 1
atom 450 450 H "TO3-E " 1
1.008 1
atom 447 447 H "TO3-E " 1
1.008 1
atom 449 449 H "TO3-E " 1
1.008 1
atom 445 445 H "TO3-E " 1
1.008 1
atom 438 438 H "TO3-E " 1
1.008 1
atom 441 441 H "TO3-E " 1
1.008 1
atom 444 444 H "TO3-E " 1
1.008 1
atom 442 442 H "TO3-E " 1
1.008 1
atom 439 439 H "TO3-E " 1
1.008 1
atom 407 407 C "TO3-E " 6
12.011 3
atom 436 436 O "TO3-E " 8
15.999 1
atom 405 405 N "TO3-E " 7
14.007 3
atom 435 435 H "TO3-E " 1
1.008 1
atom 401 401 C "TO3-E " 6
12.011 4
atom 432 432 H "TO3-E " 1
1.008 1
atom 402 402 C "TO3-E " 6
12.011 4
atom 431 431 H "TO3-E " 1
1.008 1
atom 429 429 O "TO3-E " 8
15.999 2
atom 404 404 C "TO3-E " 6
12.011 4
atom 434 434 H "TO3-E " 1
1.008 1

vdw 429 3.4050 0.1100
vdw 431 2.8700 0.0240 0.910
vdw 403 3.8200 0.1010
vdw 401 3.8200 0.1010
vdw 402 3.8200 0.1010
vdw 404 3.8200 0.1010
vdw 433 2.9800 0.0240 0.920
vdw 434 2.8900 0.0240 0.910
vdw 432 2.9600 0.0220 0.920
vdw 420 3.8200 0.1060
vdw 425 3.8200 0.1060
vdw 410 3.8200 0.1060
vdw 407 3.8200 0.1060
vdw 436 3.3000 0.1120
vdw 405 3.7100 0.1100
vdw 435 2.5900 0.0220 0.900
vdw 443 2.9200 0.0300 0.920
vdw 448 2.9200 0.0300 0.920
vdw 446 2.9200 0.0300 0.920
vdw 438 2.9200 0.0300 0.920
vdw 430 4.0050 0.3550
vdw 419 3.8200 0.1060
vdw 423 3.8000 0.0890
vdw 418 3.8000 0.0890
vdw 437 2.9800 0.0260 0.920
vdw 450 2.9800 0.0260 0.920
vdw 445 2.9800 0.0260 0.920
vdw 441 2.9800 0.0260 0.920
vdw 439 2.9800 0.0260 0.920
vdw 413 3.8000 0.0910
vdw 426 3.8000 0.0910
vdw 440 2.9800 0.0260 0.920
vdw 447 2.9800 0.0260 0.920
vdw 449 2.9800 0.0260 0.920
vdw 444 2.9800 0.0260 0.920
vdw 442 2.9800 0.0260 0.920
vdw 406 3.7100 0.1050
vdw 409 3.8000 0.1010
vdw 415 3.8000 0.1010
vdw 422 3.8000 0.1010
vdw 427 3.8000 0.1010
vdw 428 3.8000 0.1010
vdw 424 3.8000 0.1010
vdw 411 3.8000 0.1010

vdw 416 3.8000 0.1010
vdw 421 3.8000 0.1010
vdw 417 3.8000 0.1010
vdw 412 3.8000 0.1010
vdw 408 3.8000 0.1010
vdw 414 3.7100 0.1050
bond 419 430 216.0 1.74
bond 410 414 400.0 1.33
bond 406 409 381.30 1.34
bond 407 410 385.0 1.5
bond 425 422 385.0 1.44
bond 430 427 250.0 1.75
bond 436 407 601.80 1.24
bond 446 425 400.0 1.09
bond 438 410 400.0 1.09
bond 415 420 385.0 1.46
bond 425 419 385.0 1.37
bond 414 419 600.0 1.45
bond 414 418 600.0 1.42
bond 408 406 400.0 1.3469999999999998
bond 406 403 295.419463 1.4472
bond 433 403 345.97894 1.09
bond 413 409 375.678112 1.39
bond 437 409 375.586577 1.08
bond 415 413 379.094345 1.39
bond 440 413 368.093365 1.09
bond 411 415 337.112301 1.43
bond 422 420 485.389179 1.36
bond 443 420 365.16166 1.09
bond 448 422 365.16166 1.09
bond 418 427 375.678112 1.4
bond 423 427 378.323984 1.39
bond 428 423 379.094345 1.39
bond 450 423 368.093365 1.09
bond 424 428 379.094345 1.4
bond 447 428 368.093365 1.09
bond 426 424 379.094345 1.39
bond 449 424 368.093365 1.09
bond 418 426 375.678112 1.4
bond 445 426 368.093365 1.08
bond 416 411 337.112301 1.42
bond 408 411 337.112301 1.4021
bond 421 416 379.094345 1.38
bond 441 416 368.093365 1.08

bond 417 421 379.094345 1.41
bond 444 421 368.093365 1.09
bond 412 417 379.094345 1.38
bond 442 417 368.093365 1.09
bond 408 412 375.678112 1.3817
bond 439 412 368.093365 1.08
bond 405 407 363.15688 1.36
bond 435 405 475.798234 1.02
bond 401 405 232.730342 1.47
bond 432 401 340.532535 1.09
bond 402 401 205.758598 1.52
bond 431 402 340.532535 1.1
bond 429 402 274.968492 1.4
bond 404 429 291.912992 1.43
bond 434 404 345.97894 1.09

angle	425	419	414	80.0	116.24440000000001
angle	425	419	430	53.2	130.2004
angle	419	425	446	50.0	116.2
angle	419	414	410	65.0	116.2248
angle	419	430	427	80.0	89.85
angle	418	427	430	53.2	114.01
angle	418	414	410	65.0	131.4245
angle	414	419	430	60.0	111.7
angle	414	410	438	60.0	114.77
angle	414	410	407	60.0	133.53
angle	404	429	402	88.5	107.8052
angle	401	402	429	88.0	105.22
angle	430	427	423	60.0	124.8
angle	437	409	406	58.99	116.8
angle	438	410	407	38.0	111.21
angle	446	425	422	38.0	114.61
angle	409	406	403	51.80	118.57
angle	436	407	410	65.0	116.47529999999999
angle	405	407	410	60.0	119.6379
angle	405	407	436	76.98	124.7
angle	415	420	443	50.0	114.39
angle	408	406	403	65.0	118.3764
angle	448	422	425	38.0	113.99
angle	422	420	415	60.0	123.08210000000001
angle	425	422	420	60.0	126.68280000000001
angle	419	425	422	60.0	129.18
angle	406	409	413	60.0	121.29
angle	414	418	427	60.0	111.02
angle	414	418	426	60.0	127.39

angle 408 406 409 65.0 121.34
angle 411 415 420 60.0 123.4199
angle 411 408 406 80.0 119.04
angle 412 408 406 80.0 118.2808
angle 413 415 420 60.0 117.0
angle 418 414 419 65.0 111.46
angle 415 413 409 89.183524 121.02
angle 440 413 409 47.265893 117.95
angle 437 409 413 59.926322 123.63799999999999
angle 411 415 413 59.084556 117.86
angle 440 413 415 36.770174 120.94
angle 416 411 415 78.148553 123.19
angle 408 411 415 89.183524 119.1
angle 448 422 420 57.300684 117.94
angle 443 420 422 57.300684 120.63
angle 426 418 427 76.194709 120.61
angle 428 423 427 68.376488 118.37
angle 450 423 427 29.804975 120.76
angle 418 427 423 89.183524 120.85
angle 424 428 423 74.630713 120.67
angle 447 428 423 36.770174 119.5
angle 450 423 428 36.770174 120.86
angle 426 424 428 74.630713 121.35
angle 449 424 428 36.770174 119.66
angle 447 428 424 36.770174 119.8
angle 418 426 424 89.183524 117.83
angle 445 426 424 36.770174 121.68780000000001
angle 449 424 426 36.770174 118.97
angle 445 426 418 47.265893 121.88
angle 421 416 411 59.084556 120.81
angle 441 416 411 26.510066 117.768
angle 412 408 411 59.084556 122.27369999999999
angle 408 411 416 89.183524 117.64
angle 417 421 416 74.630713 120.65
angle 444 421 416 36.770174 119.78
angle 441 416 421 36.770174 122.09589999999999
angle 412 417 421 74.630713 120.55
angle 442 417 421 36.770174 119.94
angle 444 421 417 36.770174 119.56
angle 408 412 417 89.183524 119.56
angle 439 412 417 36.770174 121.5936
angle 442 417 412 36.770174 119.48
angle 439 412 408 47.265893 119.85829999999999
angle 433 403 406 53.335255 107.4068

angle 433 403 433 36.406852 113.00880000000001

angle 435 405 407 40.855201 115.76610000000001

angle 401 405 407 87.947117 114.61710000000001

angle 432 401 405 57.622801 108.3487

angle 402 401 405 99.687701 108.4788

angle 401 405 435 29.797294 116.85

angle 431 402 401 48.260518 110.6

angle 432 401 432 30.490529 108.8989

angle 402 401 432 48.260518 110.06

angle 431 402 431 30.490529 107.64

angle 429 402 431 54.887155 111.41

angle 434 404 429 57.322419 109.45

angle 434 404 434 72.813704 109.48

strbnd	425	419	414	0	0
strbnd	425	419	430	0	0
strbnd	419	425	446	0	0
strbnd	419	414	410	0	0
strbnd	419	430	427	0	0
strbnd	418	427	430	0	0
strbnd	418	414	410	0	0
strbnd	414	419	430	0	0
strbnd	414	410	438	0	0
strbnd	414	410	407	0	0
strbnd	404	429	402	0	0
strbnd	401	402	429	0	0
strbnd	430	427	423	0	0
strbnd	437	409	406	11.50	11.50
strbnd	438	410	407	0	0
strbnd	446	425	422	0	0
strbnd	409	406	403	7.20	7.20
strbnd	436	407	410	0	0
strbnd	405	407	410	0	0
strbnd	405	407	436	18.70	18.70
strbnd	415	420	443	0	0
strbnd	408	406	403	0	0
strbnd	448	422	425	0	0
strbnd	422	420	415	0	0
strbnd	425	422	420	0	0
strbnd	419	425	422	0	0
strbnd	406	409	413	0	0
strbnd	414	418	427	0	0
strbnd	414	418	426	0	0
strbnd	408	406	409	0	0
strbnd	411	415	420	0	0

strbnd 411 408 406 0 0
strbnd 412 408 406 0 0
strbnd 413 415 420 0 0
strbnd 415 413 409 20.4528 20.4528
strbnd 440 413 409 20.4528 20.4528
strbnd 437 409 413 36.8955 36.8955
strbnd 411 415 413 20.4528 20.4528
strbnd 440 413 415 20.4528 20.4528
strbnd 416 411 415 20.4528 20.4528
strbnd 408 411 415 20.4528 20.4528
strbnd 448 422 420 34.2002 34.2002
strbnd 443 420 422 34.2002 34.2002
strbnd 426 418 427 20.4528 20.4528
strbnd 428 423 427 20.4528 20.4528
strbnd 450 423 427 20.4528 20.4528
strbnd 418 427 423 20.4528 20.4528
strbnd 424 428 423 20.4528 20.4528
strbnd 447 428 423 20.4528 20.4528
strbnd 450 423 428 20.4528 20.4528
strbnd 426 424 428 20.4528 20.4528
strbnd 449 424 428 20.4528 20.4528
strbnd 447 428 424 20.4528 20.4528
strbnd 418 426 424 20.4528 20.4528
strbnd 445 426 424 20.4528 20.4528
strbnd 449 424 426 20.4528 20.4528
strbnd 445 426 418 20.4528 20.4528
strbnd 421 416 411 20.4528 20.4528
strbnd 441 416 411 20.4528 20.4528
strbnd 412 408 411 20.4528 20.4528
strbnd 408 411 416 20.4528 20.4528
strbnd 417 421 416 20.4528 20.4528
strbnd 444 421 416 20.4528 20.4528
strbnd 441 416 421 20.4528 20.4528
strbnd 412 417 421 20.4528 20.4528
strbnd 442 417 421 20.4528 20.4528
strbnd 444 421 417 20.4528 20.4528
strbnd 408 412 417 20.4528 20.4528
strbnd 439 412 417 20.4528 20.4528
strbnd 442 417 412 20.4528 20.4528
strbnd 439 412 408 20.4528 20.4528
strbnd 433 403 406 5.7126 5.7126
strbnd 433 403 433 5.7126 5.7126
strbnd 435 405 407 25.9516 25.9516
strbnd 401 405 407 25.9516 25.9516

strbnd 432 401 405 5.7126 5.7126
strbnd 402 401 405 5.7126 5.7126
strbnd 401 405 435 25.9516 25.9516
strbnd 431 402 401 5.7126 5.7126
strbnd 432 401 432 5.7126 5.7126
strbnd 402 401 432 5.7126 5.7126
strbnd 431 402 431 5.7126 5.7126
strbnd 429 402 431 5.7126 5.7126
strbnd 434 404 429 5.7126 5.7126
strbnd 434 404 434 5.7126 5.7126
opbend 410 407 0 0 42.40
opbend 443 420 0 0 140.30
opbend 448 422 0 0 140.30
opbend 446 425 0 0 140.30
opbend 438 410 0 0 140.30
opbend 415 420 0 0 42.40
opbend 403 406 0 0 12.90
opbend 422 425 0 0 42.40
opbend 422 420 0 0 14.40
opbend 419 425 0 0 14.40
opbend 414 419 0 0 18.00
opbend 414 418 0 0 18.00
opbend 420 415 0 0 14.40
opbend 406 408 0 0 14.40
opbend 419 414 0 0 10.80
opbend 418 414 0 0 10.80
opbend 413 409 0 0 34.0822
opbend 409 413 0 0 34.0822
opbend 437 409 0 0 72.8135
opbend 415 413 0 0 84.8861
opbend 413 415 0 0 84.8861
opbend 440 413 0 0 72.8135
opbend 411 415 0 0 100.1116
opbend 415 411 0 0 100.1116
opbend 418 427 0 0 47.0937
opbend 427 418 0 0 47.0937
opbend 423 427 0 0 14.9839
opbend 427 423 0 0 14.9839
opbend 428 423 0 0 84.8861
opbend 423 428 0 0 84.8861
opbend 450 423 0 0 72.8135
opbend 424 428 0 0 84.8861
opbend 428 424 0 0 84.8861
opbend 447 428 0 0 72.8135

opbend 426 424 0 0 84.8861
opbend 424 426 0 0 84.8861
opbend 449 424 0 0 72.8135
opbend 418 426 0 0 39.6142
opbend 426 418 0 0 39.6142
opbend 445 426 0 0 72.8135
opbend 416 411 0 0 95.117
opbend 411 416 0 0 95.117
opbend 408 411 0 0 47.0937
opbend 411 408 0 0 47.0937
opbend 421 416 0 0 84.8861
opbend 416 421 0 0 84.8861
opbend 441 416 0 0 72.8135
opbend 417 421 0 0 84.8861
opbend 421 417 0 0 84.8861
opbend 444 421 0 0 72.8135
opbend 412 417 0 0 84.8861
opbend 417 412 0 0 84.8861
opbend 442 417 0 0 72.8135
opbend 408 412 0 0 47.0937
opbend 412 408 0 0 47.0937
opbend 439 412 0 0 72.8135
opbend 436 407 0 0 16.5045
opbend 405 407 0 0 92.7841
opbend 407 405 0 0 92.7841
opbend 435 405 0 0 26.8945
opbend 401 405 0 0 16.5457
opbend 406 409 0 0 14.39
opbend 409 406 0 0 3.6
opbend 420 422 0 0 14.39
opbend 425 419 0 0 14.39
opbend 425 422 0 0 14.39
opbend 430 419 0 0 14.39
opbend 414 410 0 0 14.39
opbend 410 414 0 0 3.6
opbend 430 427 0 0 14.39
opbend 407 410 0 0 14.39
opbend 408 406 0 0 3.6
torsion 425 422 420 415 0.000 0.0 1 0.000 180.0 2 0.299 0.0 3
torsion 448 422 420 415 0.000 0.0 1 0.000 180.0 2 0.299 0.0 3
torsion 420 422 425 419 12.504 0.0 1 -9.345 180.0 2 2.432 0.0 3
torsion 414 419 425 422 0.000 0.0 1 0.000 180.0 2 0.299 0.0 3
torsion 430 419 425 422 0.000 0.0 1 0.000 180.0 2 0.299 0.0 3
torsion 443 420 422 425 0.000 0.0 1 0.000 180.0 2 0.299 0.0 3

torsion 418 414 419 425 -0.67 0.0 1 6.287 180.0 2 0 0.0 3
torsion 410 414 419 425 -0.67 0.0 1 6.287 180.0 2 0 0.0 3
torsion 427 430 419 425 0.854 0.0 1 -0.374 180.0 2 0.108 0.0 3
torsion 448 422 425 419 0 0.0 1 0 180.0 2 0.108 0.0 3
torsion 438 410 414 419 0.000 0.0 1 0.000 180.0 2 0.299 0.0 3
torsion 407 410 414 419 0.000 0.0 1 0.000 180.0 2 0.299 0.0 3
torsion 418 427 430 419 0.854 0.0 1 -0.374 180.0 2 0.108 0.0 3
torsion 423 427 430 419 0.854 0.0 1 -0.374 180.0 2 0.108 0.0 3
torsion 410 414 418 427 -0.67 0.0 1 6.287 180.0 2 0 0.0 3
torsion 414 419 430 427 0.854 0.0 1 -0.374 180.0 2 0.108 0.0 3
torsion 430 427 418 426 -0.67 0.0 1 6.287 180.0 2 0 0.0 3
torsion 410 414 418 426 -0.67 0.0 1 6.287 180.0 2 0 0.0 3
torsion 430 419 414 418 -0.67 0.0 1 6.287 180.0 2 0 0.0 3
torsion 438 410 414 418 0.000 0.0 1 0.000 180.0 2 0.299 0.0 3
torsion 407 410 414 418 0.000 0.0 1 0.000 180.0 2 0.299 0.0 3
torsion 430 419 414 410 -0.67 0.0 1 6.287 180.0 2 0 0.0 3
torsion 446 425 419 414 0.000 0.0 1 0.000 180.0 2 0.299 0.0 3
torsion 430 427 418 414 -0.67 0.0 1 6.287 180.0 2 0 0.0 3
torsion 436 407 410 414 0.747 0.0 1 -0.991 180.0 2 -1.637 0.0 3
torsion 405 407 410 414 1.838 0.0 1 -0.988 180.0 2 0.608 0.0 3
torsion 446 425 419 430 0.000 0.0 1 0.000 180.0 2 0.299 0.0 3
torsion 448 422 420 443 0.000 0.0 1 0.000 180.0 2 0.299 0.0 3
torsion 405 401 402 429 -2.572 0.0 1 2.571 180.0 2 -2.571 0.0 3
torsion 401 402 429 404 -1.165 0.0 1 -0.066 180.0 2 0.911 0.0 3
torsion 434 404 429 402 0 0.0 1 0 180.0 2 0.108 0.0 3
torsion 430 427 423 428 -0.67 0.0 1 6.287 180.0 2 0 0.0 3
torsion 430 427 423 450 0.55 0.0 1 6.187 180.0 2 -0.55 0.0 3
torsion 403 406 409 413 -0.67 0.0 1 6.287 180.0 2 0 0.0 3
torsion 433 403 406 409 0.072 0.0 1 -0.012 180.0 2 0.563 0.0 3
torsion 437 409 406 403 0.121 0.0 1 -0.648 180.0 2 0.199 0.0 3
torsion 436 407 410 438 0 0.0 1 0 180.0 2 0.108 0.0 3
torsion 405 407 410 438 0 0.0 1 0 180.0 2 0.108 0.0 3
torsion 433 403 406 408 0 0.0 1 0 180.0 2 0.108 0.0 3
torsion 403 406 408 412 -0.67 0.0 1 6.287 180.0 2 0 0.0 3
torsion 401 405 407 410 -1.000 0.0 1 4.283333333333333 180.0 2 2.050 0.0 3
torsion 435 405 407 410 0.000 0.0 1 3.4833333333333334 180.0 2 0.800 0.0 3
torsion 436 407 405 401 1.000 0.0 1 9.1 180.0 2 -2.250 0.0 3
torsion 435 405 407 436 0.000 0.0 1 1.6193333333333333 180.0 2 -0.357 0.0 3
torsion 406 409 413 415 -0.67 0.0 1 6.287 180.0 2 0 0.0 3
torsion 414 418 427 423 -0.67 0.0 1 6.287 180.0 2 0 0.0 3
torsion 414 418 426 424 -0.67 0.0 1 6.287 180.0 2 0 0.0 3
torsion 440 413 409 437 0.000 0.0 1 10.922 180.0 2 0.000 0.0 3
torsion 406 409 413 440 0.55 0.0 1 6.187 180.0 2 -0.55 0.0 3
torsion 414 418 426 445 0.55 0.0 1 6.187 180.0 2 -0.55 0.0 3

torsion 419 414 418 427 -0.67 0.0 1 6.287 180.0 2 0 0.0 3
torsion 419 414 418 426 -0.67 0.0 1 6.287 180.0 2 0 0.0 3
torsion 424 426 418 427 -0.67 0.0 1 6.287 180.0 2 0 0.0 3
torsion 418 427 423 428 -0.67 0.0 1 6.287 180.0 2 0 0.0 3
torsion 418 426 424 428 -0.67 0.0 1 6.287 180.0 2 0 0.0 3
torsion 447 428 423 427 0.55 0.0 1 6.187 180.0 2 -0.55 0.0 3
torsion 449 424 426 418 0.55 0.0 1 6.187 180.0 2 -0.55 0.0 3
torsion 449 424 428 447 0.000 0.0 1 10.922 180.0 2 0.000 0.0 3
torsion 411 408 406 403 -0.67 0.0 1 6.287 180.0 2 0 0.0 3
torsion 408 406 409 437 0.55 0.0 1 6.187 180.0 2 -0.55 0.0 3
torsion 411 408 406 409 -0.67 0.0 1 6.287 180.0 2 0 0.0 3
torsion 412 408 406 409 -0.67 0.0 1 6.287 180.0 2 0 0.0 3
torsion 413 409 406 408 -0.67 0.0 1 6.287 180.0 2 0 0.0 3
torsion 416 411 415 420 -0.67 0.0 1 6.287 180.0 2 0 0.0 3
torsion 408 411 415 420 -0.67 0.0 1 6.287 180.0 2 0 0.0 3
torsion 447 428 423 450 0.000 0.0 1 7.072 180.0 2 0.000 0.0 3
torsion 445 426 424 449 0.000 0.0 1 7.072 180.0 2 0.000 0.0 3
torsion 444 421 416 441 0.000 0.0 1 7.072 180.0 2 0.000 0.0 3
torsion 442 417 421 444 0.000 0.0 1 7.072 180.0 2 0.000 0.0 3
torsion 439 412 417 442 0.000 0.0 1 7.072 180.0 2 0.000 0.0 3
torsion 406 408 412 439 0.55 0.0 1 6.187 180.0 2 -0.55 0.0 3
torsion 415 411 416 441 0.250 0.0 1 5.534 180.0 2 -0.550 0.0 3
torsion 427 418 426 445 0.55 0.0 1 6.187 180.0 2 -0.55 0.0 3
torsion 423 428 424 449 0.250 0.0 1 5.534 180.0 2 -0.550 0.0 3
torsion 428 424 426 445 0.250 0.0 1 5.534 180.0 2 -0.550 0.0 3
torsion 424 428 423 450 0.250 0.0 1 5.534 180.0 2 -0.550 0.0 3
torsion 426 424 428 447 0.250 0.0 1 5.534 180.0 2 -0.550 0.0 3
torsion 418 427 423 450 0.55 0.0 1 6.187 180.0 2 -0.55 0.0 3
torsion 411 416 421 444 0.250 0.0 1 5.534 180.0 2 -0.550 0.0 3
torsion 411 408 412 439 0.250 0.0 1 5.534 180.0 2 -0.550 0.0 3
torsion 416 421 417 442 0.250 0.0 1 5.534 180.0 2 -0.550 0.0 3
torsion 421 417 412 439 0.250 0.0 1 5.534 180.0 2 -0.550 0.0 3
torsion 417 421 416 441 0.250 0.0 1 5.534 180.0 2 -0.550 0.0 3
torsion 412 417 421 444 0.250 0.0 1 5.534 180.0 2 -0.550 0.0 3
torsion 408 411 416 441 0.250 0.0 1 5.534 180.0 2 -0.550 0.0 3
torsion 408 412 417 442 0.250 0.0 1 5.534 180.0 2 -0.550 0.0 3
torsion 415 411 408 406 -0.67 0.0 1 6.287 180.0 2 0 0.0 3
torsion 416 411 408 406 -0.67 0.0 1 6.287 180.0 2 0 0.0 3
torsion 417 412 408 406 -0.67 0.0 1 6.287 180.0 2 0 0.0 3
torsion 421 416 411 415 -0.670 0.0 1 4.304 180.0 2 0.000 0.0 3
torsion 412 408 411 415 -0.670 0.0 1 4.304 180.0 2 0.000 0.0 3
torsion 424 428 423 427 -0.67 0.0 1 6.287 180.0 2 0 0.0 3
torsion 426 418 427 423 -0.67 0.0 1 6.287 180.0 2 0 0.0 3
torsion 426 424 428 423 -0.670 0.0 1 4.304 180.0 2 0.000 0.0 3

torsion 417 421 416 411 -0.670 0.0 1 4.304 180.0 2 0.000 0.0 3
torsion 417 412 408 411 -0.670 0.0 1 4.304 180.0 2 0.000 0.0 3
torsion 412 408 411 416 -0.670 0.0 1 4.304 180.0 2 0.000 0.0 3
torsion 412 417 421 416 -0.670 0.0 1 4.304 180.0 2 0.000 0.0 3
torsion 408 411 416 421 -0.670 0.0 1 4.304 180.0 2 0.000 0.0 3
torsion 408 412 417 421 -0.670 0.0 1 4.304 180.0 2 0.000 0.0 3
torsion 413 415 411 416 -0.610 0.0 1 4.212 180.0 2 0.000 0.0 3
torsion 413 415 411 408 -0.610 0.0 1 4.212 180.0 2 0.000 0.0 3
torsion 420 415 413 440 0.55 0.0 1 6.187 180.0 2 -0.55 0.0 3
torsion 411 415 413 440 0.000 0.0 1 0.000 180.0 2 0.341 0.0 3
torsion 409 413 415 420 -0.67 0.0 1 6.287 180.0 2 0 0.0 3
torsion 409 413 415 411 0.260 0.0 1 -0.255 180.0 2 0.260 0.0 3
torsion 415 413 409 437 0.000 0.0 1 0.000 180.0 2 0.341 0.0 3
torsion 432 401 405 435 1.397 0 1 0.709 180 2 -0.436 0 3
torsion 420 422 425 446 -4.964 0.0 1 6.384 180.0 2 -1.126 0.0 3
torsion 448 422 425 446 -12.504 0.0 1 10.836 180.0 2 -2.478 0.0 3
torsion 432 401 405 407 0.0 0 1 0.0 180 2 0.108 0 3
torsion 431 402 429 404 0.622 0.0 1 -0.653 180.0 2 0.259 0.0 3
torsion 405 401 402 431 -0.076 0.0 1 1.259 180.0 2 0.652 0.0 3
torsion 432 401 402 431 1.413 0.0 1 -2.181 180.0 2 0.500 0.0 3
torsion 402 401 405 435 0.0 0 1 0.0 180 2 0.108 0 3
torsion 432 401 402 429 0.0 0 1 0.0 180 2 0.108 0 3
torsion 411 415 420 422 -1.188 0 1 1.147 180 2 -0.917 0 3
torsion 443 420 415 411 0.0 0 1 0.0 180 2 0.108 0 3
torsion 402 401 405 407 -0.879 0 1 -0.305 180 2 -1.398 0 3
torsion 422 420 415 413 -0.176 0 1 2.336 180 2 -0.176 0 3
torsion 443 420 415 413 0.0 0 1 0.0 180 2 0.108 0 3
SOLUTE 437 2.9059 2.602 2.5726
SOLUTE 440 2.574 2.758 2.9054
SOLUTE 443 2.574 2.758 2.9054
SOLUTE 448 2.574 2.758 2.9054
SOLUTE 446 2.574 2.758 2.9054
SOLUTE 450 2.574 2.758 2.9054
SOLUTE 447 2.574 2.758 2.9054
SOLUTE 449 2.574 2.758 2.9054
SOLUTE 445 2.574 2.758 2.9054
SOLUTE 438 2.574 2.758 2.9054
SOLUTE 441 2.574 2.758 2.9054
SOLUTE 444 2.574 2.758 2.9054
SOLUTE 442 2.574 2.758 2.9054
SOLUTE 439 2.574 2.758 2.9054
SOLUTE 432 3.0556 2.996 4.032
SOLUTE 434 2.6533 3.392 2.9044
SOLUTE 435 3.6491 3.404 2.9894

SOLUTE 431 3.9912 4.144 3.8925
SOLUTE 433 3.143 3.374 4.144
SOLUTE 409 3.8286 3.893 4.5084
SOLUTE 419 3.8286 3.893 4.5084
SOLUTE 418 3.8585 3.766 3.8448
SOLUTE 408 3.8286 3.893 4.5084
SOLUTE 413 3.8286 3.893 4.5084
SOLUTE 415 3.8286 3.893 4.5084
SOLUTE 427 3.8585 3.766 3.8448
SOLUTE 423 3.8286 3.893 4.5084
SOLUTE 426 3.8286 3.893 4.5084
SOLUTE 411 3.8286 3.893 4.5084
SOLUTE 416 3.8286 3.893 4.5084
SOLUTE 412 3.8286 3.893 4.5084
SOLUTE 407 2.9301 4.506 4.2118
SOLUTE 428 3.8286 3.893 4.5084
SOLUTE 424 3.8286 3.893 4.5084
SOLUTE 421 3.8286 3.893 4.5084
SOLUTE 417 3.8286 3.893 4.5084
SOLUTE 420 2.9301 4.506 4.2118
SOLUTE 422 2.9301 4.506 4.2118
SOLUTE 425 2.9301 4.506 4.2118
SOLUTE 410 2.9301 4.506 4.2118
SOLUTE 403 3.5062 3.309 4.536
SOLUTE 404 3.5062 3.309 4.536
SOLUTE 401 3.3025 2.854 4.9016
SOLUTE 402 3.3025 2.854 4.9016
SOLUTE 406 3.4243 3.491 4.2676
SOLUTE 414 3.4243 3.491 4.2676
SOLUTE 405 2.8836 2.99 3.218
SOLUTE 436 2.9835 3.356 2.9616
SOLUTE 429 3.1684 3.134 3.0999
SOLUTE 430 4.4346 4.194 4.5431
polarize 403 1.6196 0.3900 433
polarize 409 2.0645 0.3900 413 406 437
polarize 413 2.0645 0.3900 409 415 440
polarize 415 2.0645 0.3900 413 411
polarize 420 1.4150 0.3900 443
polarize 422 2.0645 0.3900 448
polarize 425 1.4150 0.3900 446
polarize 419 2.0645 0.3900 414 430
polarize 427 2.0645 0.3900 423 418 430
polarize 423 2.0645 0.3900 427 428 450
polarize 428 2.0645 0.3900 423 424 447

polarize 424 2.0645 0.3900 428 426 449
polarize 426 2.0645 0.3900 424 418 445
polarize 418 2.0645 0.3900 427 426 414
polarize 410 1.4150 0.3900 438
polarize 411 2.0645 0.3900 415 416 408
polarize 416 2.0645 0.3900 411 421 441
polarize 421 2.0645 0.3900 416 417 444
polarize 417 2.0645 0.3900 421 412 442
polarize 412 2.0645 0.3900 417 408 439
polarize 408 2.0645 0.3900 411 412 406
polarize 406 1.7018 0.3900 409 408
polarize 414 1.7018 0.3900 419 418
polarize 430 3.2059 0.3900 419 427
polarize 433 0.4803 0.3900 403
polarize 437 0.4318 0.3900 409
polarize 440 0.4318 0.3900 413
polarize 443 0.4318 0.3900 420
polarize 448 0.4318 0.3900 422
polarize 446 0.4803 0.3900 425
polarize 450 0.4318 0.3900 423
polarize 447 0.4318 0.3900 428
polarize 449 0.4318 0.3900 424
polarize 445 0.4318 0.3900 426
polarize 438 0.4803 0.3900 410
polarize 441 0.4318 0.3900 416
polarize 444 0.4318 0.3900 421
polarize 442 0.4318 0.3900 417
polarize 439 0.4318 0.3900 412
polarize 407 2.0645 0.3900 436 405
polarize 436 0.9138 0.3900 407
polarize 405 1.2433 0.3900 407 435
polarize 435 0.4573 0.3900 405
polarize 401 1.6196 0.3900 432
polarize 432 0.4803 0.3900 401
polarize 402 1.6196 0.3900 431 429
polarize 431 0.4803 0.3900 402
polarize 429 0.8122 0.3900 402 404
polarize 404 1.6196 0.3900 429 434
polarize 434 0.4803 0.3900 404
multipole 403 406 408 -0.07427
-0.04534 0.00000 0.43601
-0.47165
0.00000 -0.43942
-0.07611 0.00000 0.91107

multipole	409	437	406	0.24552		
				0.21240	0.00000	-0.18674
				0.01602		
				0.00000	-0.53054	
				-0.14121	0.00000	0.51452
multipole	413	440	415	-0.09886		
				0.00874	0.00000	0.26456
				0.26031		
				0.00000	-0.05638	
				0.15106	0.00000	-0.20393
multipole	415	411	420	0.12227		
				0.31099	0.00000	-0.06269
				0.28419		
				0.00000	0.31541	
				0.43536	0.00000	-0.59960
multipole	420	443	422	-0.10008		
				-0.11973	0.00000	0.17683
				-0.22853		
				0.00000	0.18618	
				-0.11052	0.00000	0.04235
multipole	422	448	425	-0.12184		
				0.14300	0.00000	-0.03078
				-0.04906		
				0.00000	0.15625	
				0.09063	0.00000	-0.10719
multipole	425	446	419	0.07128		
				-0.00760	0.00000	-0.16482
				0.01113		
				0.00000	-0.13075	
				-0.15159	0.00000	0.11962
multipole	419	430	414	-0.33573		
				0.05734	0.00000	-0.15963
				0.41520		
				0.00000	-0.11749	
				-0.36797	0.00000	-0.29771
multipole	427	430	418	-0.19537		
				-0.00958	0.00000	-0.15805
				0.01733		
				0.00000	-0.02430	
				-0.18997	0.00000	0.00697
multipole	423	450	428	-0.08001		
				-0.22464	0.00000	0.18685
				0.01803		
				0.00000	0.07204	

				-0.05903	0.00000	-0.09007
multipole	428	447	424	-0.00857		
				0.01491	0.00000	0.22064
				0.26540		
				0.00000	-0.03842	
				-0.00073	0.00000	-0.22698
multipole	424	449	426	0.28987		
				-0.05892	0.00000	-0.49393
				-0.93825		
				0.00000	-0.83585	
				0.14921	0.00000	1.77410
multipole	426	445	418	-0.09442		
				0.09308	0.00000	0.14249
				0.04240		
				0.00000	0.10295	
				-0.01357	0.00000	-0.14535
multipole	418	414	426	-0.03625		
				-0.05872	0.00000	0.24976
				-0.31397		
				0.00000	0.00966	
				0.09209	0.00000	0.30431
multipole	410	407	438	-0.03108		
				-0.21460	0.00000	-0.03980
				-0.18766		
				0.00000	0.14409	
				0.57826	0.00000	0.04357
multipole	411	408	416	-0.04598		
				0.16201	0.00000	0.03346
				-0.52308		
				0.00000	0.61501	
				-0.07957	0.00000	-0.09193
multipole	416	441	421	-0.16937		
				0.03187	0.00000	0.21548
				-0.02480		
				0.00000	0.11593	
				-0.22898	0.00000	-0.09113
multipole	421	444	417	0.21376		
				0.04445	0.00000	-0.21321
				-0.51415		
				0.00000	-0.48991	
				-0.06162	0.00000	1.00406
multipole	417	442	412	0.00021		
				-0.08919	0.00000	0.18633
				0.24288		

0.00000 -0.02997
0.00945 0.00000 -0.21291
multipole 412 439 408 -0.10153
0.22323 0.00000 0.10499
0.05821
0.00000 0.02258
-0.16896 0.00000 -0.08079
multipole 408 406 412 0.25705
0.02471 0.00000 0.11832
-0.37522
0.00000 -0.55282
-0.59208 0.00000 0.92804
multipole 406 408 409 0.03353
0.00242 0.00000 -0.01137
1.15091
0.00000 -0.85046
0.73926 0.00000 -0.30045
multipole 414 410 418 0.26268
-0.27474 0.00000 0.08473
0.20265
0.00000 -0.44091
0.21667 0.00000 0.23826
multipole 430 427 419 0.52446
0.63453 0.00000 0.46423
1.11399
0.00000 -2.03975
-0.03780 0.00000 0.92576
multipole 433 403 406 0.10269
0.02702 0.00000 -0.02602
0.02000
0.00000 -0.03372
0.02108 0.00000 0.01372
multipole 437 409 406 0.07790
0.01612 0.00000 -0.07536
0.05382
0.00000 -0.06315
0.00217 0.00000 0.00933
multipole 440 413 415 0.08144
0.00621 0.00000 -0.12117
0.04693
0.00000 -0.02665
0.00010 0.00000 -0.02028
multipole 443 420 422 0.03959
0.08796 0.00000 -0.17056

				0.26868		
				0.00000	-0.10024	
				-0.01683	0.00000	-0.16844
multipole	448	422	425	0.10890		
				0.01340	0.00000	-0.07678
				0.03094		
				0.00000	-0.03092	
				0.04475	0.00000	-0.00002
multipole	446	425	419	0.14882		
				0.07358	0.00000	-0.09689
				0.10991		
				0.00000	-0.08313	
				0.03743	0.00000	-0.02678
multipole	450	423	428	0.04281		
				0.02419	0.00000	-0.14897
				0.09347		
				0.00000	-0.03538	
				0.00093	0.00000	-0.05809
multipole	447	428	424	0.05488		
				-0.02454	0.00000	-0.10892
				0.04192		
				0.00000	-0.03072	
				-0.02521	0.00000	-0.01120
multipole	449	424	426	0.05260		
				-0.00230	0.00000	0.03004
				-0.02274		
				0.00000	-0.09940	
				-0.00952	0.00000	0.12214
multipole	445	426	418	0.08142		
				-0.03458	0.00000	-0.15042
				0.14665		
				0.00000	0.03488	
				-0.05653	0.00000	-0.18153
multipole	438	410	407	0.09344		
				-0.02141	0.00000	-0.13402
				0.18996		
				0.00000	-0.02634	
				0.08185	0.00000	-0.16362
multipole	441	416	421	0.03410		
				0.07648	0.00000	-0.17264
				0.01582		
				0.00000	0.02528	
				-0.00071	0.00000	-0.04110
multipole	444	421	417	0.05126		

-0.04429 0.00000 -0.03261
0.01535
0.00000 -0.08229
-0.04372 0.00000 0.06694
multipole 442 417 412 0.05131
0.01592 0.00000 -0.12772
0.03687
0.00000 -0.01513
0.00734 0.00000 -0.02174
multipole 439 412 408 0.07467
0.00798 0.00000 -0.18807
0.18894
0.00000 -0.05680
-0.06533 0.00000 -0.13214
multipole 407 405 436 0.68553
0.11323 0.00000 0.09403
0.17489
0.00000 -0.19552
0.00870 0.00000 0.02063
multipole 436 407 405 -0.58634
-0.05726 0.00000 -0.02422
-0.47142
0.00000 0.19851
-0.04488 0.00000 0.27291
multipole 405 401 435 -0.20801
0.13417 0.00000 0.51555
-0.04042
0.00000 -0.90617
-0.20582 0.00000 0.94659
multipole 435 405 401 0.11335
-0.02492 0.00000 -0.11035
-0.03373
0.00000 -0.08429
0.07023 0.00000 0.11802
multipole 401 402 405 -0.08318
0.38559 0.00000 0.22141
0.33519
0.00000 -0.65728
-0.41855 0.00000 0.32209
multipole 432 401 402 0.09029
0.01265 0.00000 0.02521
-0.01361
0.00000 -0.00365
0.04162 0.00000 0.01726

multipole	402 429 401		0.18260		
			0.35324	0.00000	0.06951
			-0.06113		
			0.00000	-0.31230	
			-0.16991	0.00000	0.37343
multipole	431 402 429		0.00261		
			-0.01926	0.00000	-0.07179
			0.06983		
			0.00000	0.00216	
			-0.03453	0.00000	-0.07199
multipole	429 404 402		-0.31988		
			0.29821	0.00000	0.22281
			0.14167		
			0.00000	-0.93403	
			-0.69844	0.00000	0.79236
multipole	404 -434 -434 -434		0.07601		
			0.00270	0.00000	-0.26969
			-0.44750		
			0.00000	-0.44027	
			-0.02878	0.00000	0.88777
multipole	434 404 429		0.04188		
			0.05369	0.00000	-0.04570
			-0.01856		
			0.00000	-0.01890	
			0.03864	0.00000	0.03746
